# Synergy from gene expression and network mining (SynGeNet) method predicts synergistic drug combinations for diverse melanoma genomic subtypes

**DOI:** 10.1038/s41540-019-0085-4

**Published:** 2019-02-26

**Authors:** Kelly E. Regan-Fendt, Jielin Xu, Mallory DiVincenzo, Megan C. Duggan, Reena Shakya, Ryejung Na, William E. Carson, Philip R. O. Payne, Fuhai Li

**Affiliations:** 1 Department of Biomedical Informatics, The Ohio State University, Columbus, OH USA; 2 Comprehensive Cancer Center, The Ohio State University, Columbus, OH USA; 3 Target Validation Shared Resource, The Ohio State University, Columbus, OH USA; 4 Institute for Informatics, Washington University in St. Louis, St. Louis, MO USA; 5 Department of Pediatrics, Washington University in St. Louis, St. Louis, MO USA

## Abstract

Systems biology perspectives are crucial for understanding the pathophysiology of complex diseases, and therefore hold great promise for the discovery of novel treatment strategies. Drug combinations have been shown to improve durability and reduce resistance to available first-line therapies in a variety of cancers; however, traditional drug discovery approaches are prohibitively cost and labor-intensive to evaluate large-scale matrices of potential drug combinations. Computational methods are needed to efficiently model complex interactions of drug target pathways and identify mechanisms underlying drug combination synergy. In this study, we employ a computational approach, SynGeNet (Synergy from Gene expression and Network mining), which integrates transcriptomics-based connectivity mapping and network centrality analysis to analyze disease networks and predict drug combinations. As an exemplar of a disease in which combination therapies demonstrate efficacy in genomic-specific contexts, we investigate malignant melanoma. We employed SynGeNet to generate drug combination predictions for each of the four major genomic subtypes of melanoma (BRAF, NRAS, NF1, and triple wild type) using publicly available gene expression and mutation data. We validated synergistic drug combinations predicted by our method across all genomic subtypes using results from a high-throughput drug screening study across. Finally, we prospectively validated the drug combination for *BRAF*-mutant melanoma that was top ranked by our approach, vemurafenib (BRAF inhibitor) + tretinoin (retinoic acid receptor agonist), using both in vitro and in vivo models of *BRAF*-mutant melanoma and RNA-sequencing analysis of drug-treated melanoma cells to validate the predicted mechanisms. Our approach is applicable to a wide range of disease domains, and, importantly, can model disease-relevant protein subnetworks in precision medicine contexts.

## Systems Biology: Computational method predicts drug combinations for melanoma

Existing drugs have the potential to learn “new tricks” to fight cancer when working together in combinations. Researchers from The Ohio State University and Washington University in St. Louis developed a computational approach to predict synergistic drug combinations among thousands of potential pairs based upon how cancer cells alter certain genes and connections between genes. They found that drug predictions were more accurate when this method was applied to specific molecular subtypes of melanoma. The top drug combination predicted for melanoma with *BRAF* gene mutations included a known melanoma drug, vemurafenib, in combination with another agent not currently used for melanoma, tretinoin. This combination could kill melanoma cells and reduce tumor size in mice. Future applications include other examples of “precision medicine” to deliver the right drugs to the right patients at the right time.

## Introduction

Systems medicine approaches and related computational methods have provided new and powerful means to aid in various aspects of the drug discovery process via modeling complex, multi-dimensional phenotypes that seek to overcome reductionist approaches to discovery science.^1,2^ Numerous studies have demonstrated their utility in identifying novel drug targets, predicting on- and off-target mechanisms, and accelerating the translation of drug repurposing efforts.^3,4^ Discovering novel uses for existing drugs through drug repurposing or drug repositioning has also been an important goal in efforts to advance understanding of systems-level effects of large repertoires of chemical and pharmacological agents, and in doing so, potentially reduce the financial and labor costs associated with the drug discovery process. Finally, drug combinations represent the current treatment strategy for many cancers. The cross-talk, redundancy, and feedback loops of signaling pathways regulating these complex diseases can drive resistance to single-target therapies.^5^ The design of multi-target agents and rationale drug combinations are aimed at increasing overall efficacy, improve initiation for first-line therapies, reduce and/or prevent drug resistance, and reduce drug toxicities. However, it is infeasible, with limited resources, to experimentally screen pairwise drug combinations derived from thousands of currently available therapies for synergistic effects across diverse cell lines and human-derived models.

One systems-based approach employed in this study is the widely used connectivity mapping method to facilitate systematic comparison of gene expression profiles characterizing responses to drugs and biological states of interest using pattern-matching algorithms.^6^ The accumulation of ubiquitous drug-induced gene expression profiles in publicly available datasets has permitted widespread connectivity mapping analysis, including the original Connectivity Map (CMap) database and the NIH Library of Integrated Network-based Cellular Signatures (LINCS) database containing 473,647 gene expression signatures from 42,080 perturbagens tested across 3–77 cell lines.^7^ Connectivity mapping studies via CMap and LINCS quantify the “closeness” between two biological states (e.g., disease and drug), where drugs that are determined to be “negatively connected” to a gene expression signature characterizing a disease state, and the predicted drugs are hypothesized to oppose or reverse the disease state.^8^ Connectivity mapping studies have been used in diverse applications and have been validated in in vitro and in vivo models and pursued in clinical trials in several instances.^9–11^ The main advantage of these approaches is that they offer an unbiased, global view of drug features. Similarity matrices constructed on these gene expression profiles can be exploited to cluster drugs to categorize potential mechanisms of action and generate drug combination hypotheses based on the joint dissimilarity of gene expression patterns of drug pairs as compared to those associated with disease states.^12–15^

The second approach we employed to identify drug candidates is network analysis of interactions comprising disease-relevant protein subnetworks and interactions between drugs and targets.^2,16^ One main advantage of network analysis is that drug targets can be identified in the context of pathways or interaction partners in a defined subnetwork, thus permitting a more precise understanding of molecular mechanisms.^17,18^ Computational methods can also facilitate large-scale, in silico perturbation simulations within network structures, which can also be used to identify novel drug targets.^19,20^ Others have utilized network-based methods to generate hypotheses for off-target effects of drugs and identify drug candidates for repositioning.^21–23^ Network-based analyses have also shown promise in identifying synergistic chemotherapeutic agents.^24^

Our work is also motivated by several limitations facing current applications of computational and systems-based methods in drug repurposing and drug combination discovery. For instance, a common limitation of many computational methods described in a recent review is that most models are too “target focused,” in that they rely heavily on modeling interactions between individual disease genes or and drug targets.^25^ While large-scale whole-exome sequencing (WES) and whole-genome sequencing (WGS) studies characterizing unique genomic subtypes of a variety of cancers have identified personalized therapies based on individual gene mutations, this reductionist approach may limit the understanding of biological effects of driver genes and other less frequently observed genomic aberrations. Another generic issue plaguing computational drug repurposing and drug combination prediction methods is the lack of prospective experimental validation.^25^ A recent effort to address this issue has been the systematic collection of real-world evidence of drug repurposing hypothesis, including gold standards and failed drugs for a variety of disease contexts and drug candidates.^26^ While these systematic databases and more focused experiments have validated several hypotheses generated by computational drug repurposing methods across different diseases, there has been comparably less evidence validating the underlying bioinformatics theory, as well as the potential mechanisms underlying drugs’ efficacy, synergy, and/or antagonism.^3^

Melanoma also serves as an exemplar disease for systems-based approaches to enable drug repurposing and drug combination discovery. Recently, The Cancer Genome Atlas (TCGA) consortium conducted the largest WES study of melanoma tumors to date. The TCGA study employed an integrative, multi-platform approach to analyze DNA, RNA, and protein expression of 333 primary and metastatic melanoma tumors and established a novel framework to classify melanoma tumors based on the following significant, mutually exclusive mutation patterns: *BRAF*, *NRAS*, *NF1*, and triple wild type (TWT).^27^ It is also important to note that these driver events are not sufficient alone to explain the transformation and maintenance of tumorigenesis of melanoma tumors.^28,29^ Studies integrating multi-omics data from melanoma tumors have been used to identify novel driver genes and have been shown to improve prognosis predictions over non-integrated models.^30–32^ Drug combinations are increasingly utilized to address some of these clinical challenges in melanoma. A multitude of drug combinations are being investigated in pre-clinical settings and clinical trials to improve the effectiveness of first-line targeted and immune therapies for melanoma patients.^33^ Despite these improvements, drug resistance to targeted combination therapy remains a challenge for the majority of melanoma patients. Therefore, systems approaches that can integrate and interpret heterogeneous molecular alterations are crucial to enhance our understanding of melanoma tumorigenesis, drug resistance, and discovery precision therapeutics for distinct genomic subtypes of melanoma.

To overcome some of the limitations of these systems-based approaches, we developed an integrative computational method, SynGeNet (Synergy from Gene expression and Network mining). In our previous work, we tested SynGeNet in a limited setting exclusively in *BRAF*-mutant melanoma and showed that it could outperform several other tools that use disease- and drug-associated gene expression data to generate drug combination predictions.^34^ In this study, we systematically expand and evaluate SynGeNet to predict synergistic drug combinations for all four genomic subtypes of melanoma, as well as interpret mechanisms of SynGeNet predictions. While large-scale WES and WGS studies characterizing unique genomic subtypes of melanoma have proposed personalized therapies based on individual gene mutations, this reductionist approach may limit the understanding of biological effects of driver genes and other less frequently observed genomic aberrations. Here the concept of a “target” is extended by modeling disease protein subnetworks via the integration of diverse molecular profiles to overcome the “one-target-one-drug” paradigm limitation. Specifically, we used subtype-specific genomics and transcriptomics data from melanoma patient tumors to synthesize coherent network models that optimize flow from known driver “root” genes and candidate co-mutated driver genes propagated through PPIs weighted by biological evidence and gene expression levels. The resultant protein subnetworks are then analyzed to predict drug combinations that optimize the reversal of gene expression and targeting of topologically central network nodes. To validate genomic subtype-specific drug combination predictions in this study, we utilized results from a previously published high-throughput drug screen testing drug combinations across diverse genetic backgrounds of melanoma, including all four major subtypes.^35^ Additionally, we assessed several other assumptions underlying our approach, including examining the robustness of the integrated network to individual genes, the effects of re-wiring of the connections among genes within the network, comparing genotype-specific melanoma subnetworks to generalized and randomized subnetworks, as well as evaluating the use of root genes and differentially expressed genes alone as compared to the integrated network models. Finally, we presented prospective validation of the drug combination of vemurafenib (BRAF inhibitor) and tretinoin (all-*trans* retinoic acid (ATRA)) predicted by our method for *BRAF*-mutant melanoma. Importantly, we also investigated the molecular mechanisms underlying the synergistic effects of this drug combination, as predicted by SynGeNet, including reversal of gene expression at the *BRAF* melanoma network level and at an individual gene level for the most “central” (i.e., topologically important) genes within the subnetwork.

**Fig. 1:**
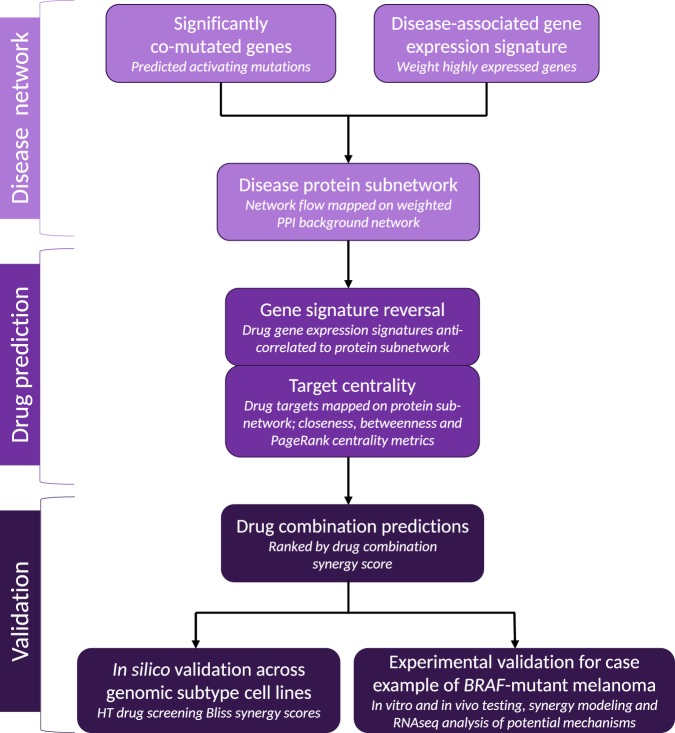
Overview of SynGeNet drug combination prediction study design. The first step of our method involves generating melanoma genotype-specific protein subnetworks from a source of disease-associated root genes (i.e., significantly co-mutated) from which network flow is propagated across a background network of protein–protein interactions (PPI) using up-regulated gene expression data (e.g., tumor vs. normal samples) via the belief propagation algorithm. Next, drug combinations are predicted using the resulting networks, where drug synergy scores are calculated based on the degree of drug-induced gene signature reversal (i.e., negative gene set enrichment analysis connectivity scores) and the weighted sum of centrality metrics calculated for the combined set drug targets in the network for each drug pair. Finally, predicted drug combinations are ranked according to a final synergy score. Drug predictions were validated in this study in two settings: (i) retrospectively, using Bliss synergy score results from a high-throughput drug screening across melanoma cell lines with different genomic backgrounds, and (ii) prospectively, where a top-ranked drug combination predicted for *BRAF*-mutant melanoma was selected as a case study for prospective validation using in vitro and in vivo models of *BRAF*-mutant melanoma, and the mechanistic basis for this drug combination prediction was investigated via RNA-seq gene expression analysis and the subnetwork level and for individual genes determined to be highly central

Due to the heterogeneous genomic landscape of melanoma, we sought to apply a systems biology framework to integrate gene variant and transcriptomic data using network analysis to characterize protein subnetworks of melanoma tumors driven by distinct driver mutations: *BRAF*, *NRAS*, and *NF1*, as well as *BRAF*/*NRAS*/*NF1* TWT. Using the resulting protein subnetworks, we applied a multi-step approach to define drug combinations that together we refer to as SynGeNet. First, we identified potential drug combinations based on (i) drug-induced gene expression signatures that maximally oppose gene signatures defined by each melanoma subnetwork and (ii) the combined set of topologically important target genes within the subnetwork determined by three centrality metrics. The overall study design workflow is presented in Fig. 1.

## Results

### Distinct protein subnetworks revealed for genomic subtypes of melanoma

**Fig. 2:**
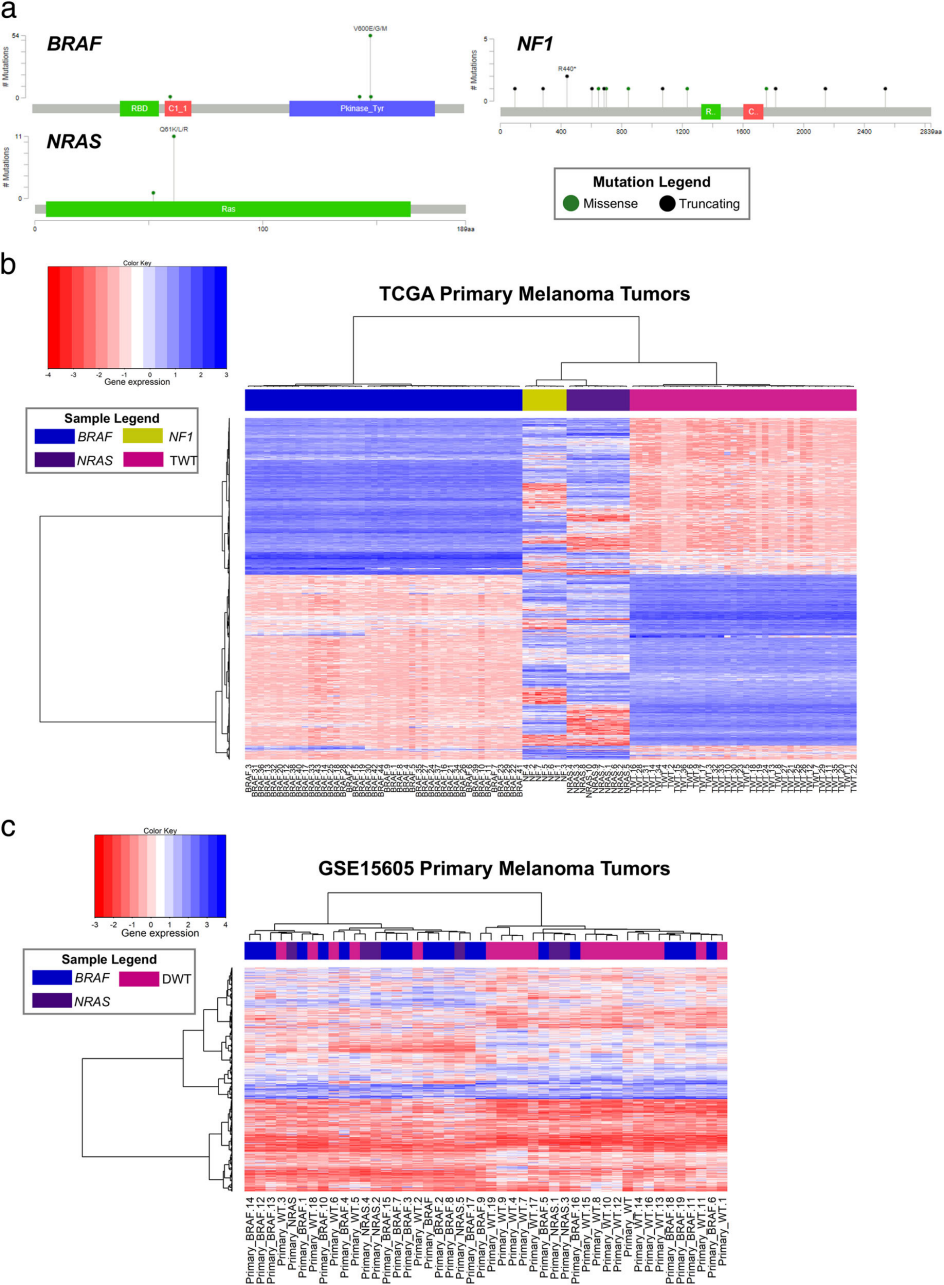
Spectrum of gene mutations and associated gene expression profiles across melanoma genomic subtypes in the The Cancer Genome Atlas Skin Cutaneous Melanoma (TCGA SKCM) dataset. **a** Gene mutation plots including location and frequency of mutations in the *BRAF*, *NRAS*, and *NF1* genes are shown for primary melanoma tumor samples in the TCGA SKCM dataset. Mutation marker height corresponds to the number of mutations and color corresponds to mutation type: missense (green) and truncating, including nonsense, nonstop, frameshift deletion, frameshift insertion, and splice site (black). Somatic mutation frequency for each gene in this cohort is as follows: *BRAF* (42.3%), NRAS (9.6%), and *NF1* (9.6%). Protein families visualized for each gene include *BRAF*: protein tyrosine kinase (457–714), C1 domain (235– 282), and Raf-like Ras-binding domain (156–225); *NRAS*: Ras family (5–165); *NF1*: GTPase-activator protein for Ras-like GTPase (1324– 1451), and CRAL/TRIO domain (1602-1736). Hierarchical clustering (Euclidean distance) of primary melanoma tumors samples from the TCGA SKCM (**b**) and GEO GSE15605 (**c**) datasets. For the TCGA SKCM dataset, sample labels are color coded according to genomic subtype: *BRAF* (blue), NRAS (purple), *NF1* (yellow), and triple wild-type (magenta). For the GSE15605 dataset, samples are color coded according to genomic subtype: *BRAF* (blue), *NRAS* (purple), and double wild-type (DWT)

To generate genomic subtype-specific protein subnetworks, we obtained gene mutation data from primary melanoma patient tumors from the TCGA database. We defined the following genomic subtype groups for patients with tumors containing mutations in the following genes: *BRAF* (*n* = 44 patients); *NRAS* (*n* = 10 patients); *NF1* (*n* = 10 patients); TWT (*n* = 36 patients). The majority of melanoma patients harboring *BRAF* and *NRAS* mutations exhibited the well-known hotspot driver mutations at the V600 (42/44 samples) and Q61 (10/10 samples) loci, respectively. Additionally, three less frequently observed mutations in *BRAF* (K601E, L245F, and N581H) and one in *NRAS* (L52W) were present in this cohort. Interestingly, mutations in *NF1* were observed at 14 different loci, with primarily truncating effects, which is consistent with the knowledge that *NF1* serves as a tumor suppressor in melanoma. The frequency and location of the mutations affecting these melanoma driver genes are visualized in Fig. 2a.

The first step in constructing protein subnetworks is to define a set of “root” genes from which network flow originates in the background protein–protein interaction (PPI) network. In addition to each mutated driver gene (*BRAF*, *NRA*, and *NF1*), we determined significantly co-mutated genes (*P* ≤ 0.05, Fisher's exact test) within each cohort, *BRAF* (*n* = 12 genes), *NRAS* (*n* = 72 genes), *NF1* (*n* = 200 genes), and TWT (*n* = 13 genes), to define a set of network root genes for each genomic subtype. We then utilized gene expression data for genes differentially expressed in melanoma tumors as compared to normal skin samples for each genomic subtype in order to generate subnetworks propagating from root genes within the background PPI network using the belief propagation algorithm, as described in the Methods section.^36^ To establish that gene expression profiles reflected differences in genomic subtype of melanoma tumors, we performed hierarchical clustering analysis using the Euclidean distance on gene expression data obtained from primary melanoma tumors in the Gene Expression Omnibus (GEO) GSE15605 and TCGA Skin Cutaneous Melanoma (SKCM) datasets for each major genomic sub-group. The corresponding heatmap and dendrogram for the TCGA SKCM and GEO GSE15605 datasets are shown in Fig. 2b, c, respectively. Remarkably, the gene expression signatures in the TCGA dataset grouped each of the four genomic subtypes into four distinct clusters (Fig. 2b). Differences in global gene expression patterns between *BRAF* and TWT samples in the TCGA dataset exhibited the most striking difference between all four groups. While the GEO dataset did not contain *NF1* mutant or TWT samples to distinguish among the *BRAF*/*NRAS* wild-type tumors, two major clusters separated the majority of BRAF-mutant primary tumors from the wild-type tumors similarly to the TCGA dataset (Fig. 2c). These results suggest that transcriptomic signatures observed in melanoma tumors may reflect biological differences in oncogenic driver gene status.

The resulting protein subnetworks for each melanoma genomic subtype are visualized in Figure S1A, and gene network node interactions for each network are provided in Table S1. As is shown in Figure S1A, the vast majority of subnetwork genes mapped by the belief propagation algorithm were highly up-regulated (green nodes). Two possible explanations accounting for the few down-regulated genes observed in each network include: (i) the genes exhibited high degree of evidence of PPIs that link multiple up-regulated genes, and/or (ii) they represented or interact with highly topologically “central” genes (larger size nodes) that are influential to the overall network structure. Importantly, because we weighted up-regulated genes (positive fold-change) to model protein subnetwork activation in the undirected PPI network, we did not include any co-mutated gene that resulted in a loss-of-function (LOF) mutation as a root gene due to the presumed loss of interaction from non-functioning genes. Interestingly, although we tested this approach including *NF1* as a root gene for the group of patients harboring predominantly truncating (i.e., LOF) mutations in this gene, the algorithm did not map *NF1* to other interacting proteins in the network, consistent with our assumption that LOF mutations would not be effectively modeled using this protein subnetwork construction approach. Furthermore, the results from our analyses demonstrated that gene expression signals could distinguish the *NF1* mutant tumors and connect other non-LOF root genes co-mutated with *NF1* through PPIs in this patient cohort, suggesting that the constructed subnetwork may reflect biological processes influenced by *NF1* mutation status.

### SynGeNet method predicts drug combinations for melanoma genomic subtypes

In this study, we sought to conduct comprehensive melanoma network analyses to identify drug predictions for diverse genomic contexts. We generated drug predictions for each subtype-specific protein subnetwork from the following steps as described in the Methods: (1) drugs were selected that induced gene expression profiles that were anti-correlated to the subnetwork via calculating connectivity scores; (2) drugs were further filtered to those with target genes mapping onto the subnetworks; (3) centrality scores were calculated for each drug target gene in the subnetwork; (4) drug synergy scores were calculated by combining the weighted connectivity score for each drug pair and the weighted sum of the combined centrality metrics for the gene targets of each drug pair; (5) drugs were clustered into communities based on the similarity of their drug-induced gene expression profiles using the affinity propagation algorithm in order to select drug pairs from distinct communities. The full lists of drug combinations predicted for each genomic subtype-specific melanoma subnetwork are shown in Table S2. As is shown in Figure S1B, the majority of predicted drug combinations are unique to each melanoma genomic subtype for *BRAF*-, *NRAS*-, and *NF1*-mutant melanoma using data from the TCGA SKCM dataset. Notably, network genes and drug predictions for TWT exhibited a higher degree of overlap with the other subtype-specific networks. We also observed similar patterns of overlap using data from the GEO GSE15605 dataset (Figure S2). Additionally, to determine whether certain drug classes were more represented in each genomic subtype, we mapped drug combinations to entities in the KEGG drug database and visualized drug class relationships in Figure S3. The major drug classes mapped across all genomic subtypes included antineoplastic and cardiovascular agents. Interestingly, *BRAF* and TWT drug combination pairs were predominated by several major classes (antineoplastic, neuropsychiatric, and cardiovascular), while *NRAS* and *NF1* drug combinations exhibited more diverse drug class pairings.

**Fig. 3:**
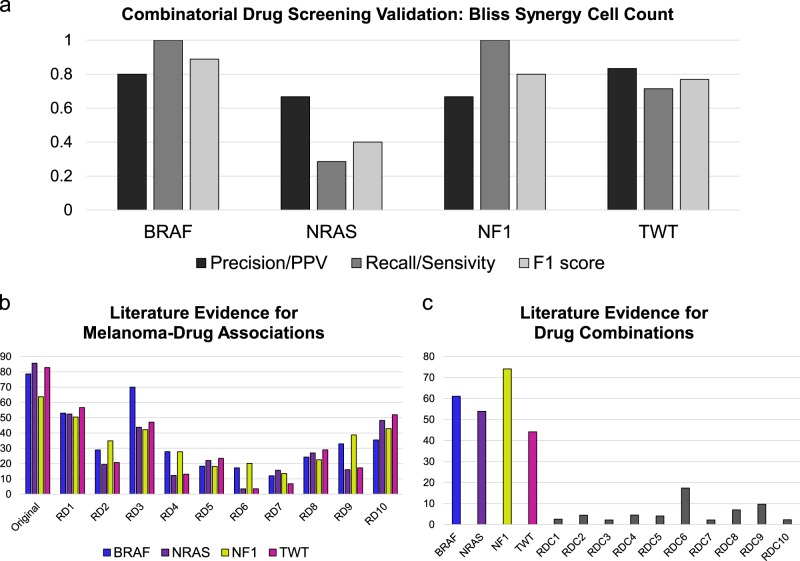
Validation of drug combination predictions across melanoma genomic subtypes using high-throughput drug screening data and literature evidence. **a** Bliss synergy scores obtained from drug combinations from a high-throughput drug screening study evaluating 5778 drug combinations among 108 drugs in *BRAF*-mutant (A375), *NRAS*-mutant (IPC-298), *NF1*- mutant (MeWo), and TWT (COLO792) cell lines were used to assess precision and recall of drug combination predictions. The geometric mean of precision and recall (F1 score) is also reported for each set of genomic subtype-specific drug combination predictions. **b** The mean number of PubMed abstracts for melanoma–drug associations for single drugs constituent of drug combinations for the original predictions and random samplings of the Food and Drug Administration (FDA)-approved drug dataset of equal size for each of the four genomic subtypes. **c** The mean number of PubMed abstracts for drug–drug associations for the top 50 drug combinations for the original drug combination predictions in each genomic subtype (color-coded bars) as well as random samplings of drug pairs (*n* = 50 pairs) from the FDA-approved drug dataset (gray bars)

To validate our drug combination predictions for each of these melanoma genomic subtypes, we utilized a large-scale high-throughput drug combination drug screening dataset that tested 5778 pairwise combinations of 108 drugs that are Food and Drug Administration (FDA) approved or in late clinical trials in all four melanoma genomic subtypes.^35^ Specifically, a matrix of Bliss synergy scores calculated by the authors for drug combinations were utilized to determine true positives (positive Bliss scores) and false positives (negative Bliss scores) for our predictions. Interestingly, drug combinations demonstrating synergistic effects in this screening study were predominantly cellular context specific. The authors noted that even drug combinations exhibiting profound synergy in a subset of cell lines were not synergistic or effective in other cell lines, including those that were expected to be broadly synergistic or even in the same genomic background. This screening study tested drug combinations in each of the four genomic subtypes of melanoma, including one *NF1*-mutant melanoma cell line (MeWo) and one TWT melanoma cell line (COLO792). We also selected the A375 (*BRAF* mutant) and IPC-298 (*NRAS* mutant) to validate drug combination predictions for these melanoma subtypes, as they represented cell lines where the full matrix of drug combinations was tested. We determined the precision and recall for genomic subtype-specific drug combinations predicted by our method applied to melanoma protein subnetworks generated from genomic and transcriptomic data for each of the four genomic subtypes from the TCGA SKCM dataset. We observed a high precision for drug combination predictions across each genomic subtype: *BRAF* (0.80), *NRAS* (0.67), *NF1* (0.67), and TWT (0.83) (Fig. 3a). While we also observed a high recall for *BRAF*, *NF1*, and TWT drug combination predictions, we found a lower recall (0.29) specifically for the IPC-298 *NRAS*-mutant melanoma cell line (Fig. 3a). We also evaluated several model assumptions on the effect of drug combination predictions and present the results in Supplemental Results (Figures S4–S9).

Lastly, to determine the broader relevance of the drug predictions in melanoma, we performed a literature analysis for melanoma–drug associations as well as drug–drug combination associations quantifying co-occurring terms from abstracts in the PubMed database. For each genomic subtype, we determined the mean number of literature associations involving drugs predicted in combination with melanoma and compared the results to literature associations generated from 10 random samplings of equal size to the original predictions for each genomic subtype from the same pool of FDA-approved drugs used to generate the drug combination predictions (Fig. 3b). Overall, we observed a higher degree of drug–melanoma literature associations for each of the four sets of drug combination predictions as compared to the sets of random drug–melanoma pairs. We also sought to determine the level of literature evidence for drug combinations predicted for each melanoma genomic subtype, as compared to random drug pairs (Fig. 3c). We first selected the top 50 drug combinations ranked for each melanoma genomic subtype and generated 10 random permutations of 50 drug pairs to search in the PubMed database. We consistently found a higher number of literature-reported drug–drug pairs for drug combination predictions across all melanoma genomic subtypes: *BRAF* (61), *NRAS* (54), *NF1* (74), and TWT (44), as compared to the average found for random drug pairs permutations (6). Taken together, these results show that our approach identified drugs associated with melanoma and drug combinations with a high degree of literature evidence compared to random sampling. Thus, these findings may suggest that our approach predicts drug combinations on the basis of well-studied biological mechanisms of melanoma and drug interactions.

### Experimental validation of drug combination prediction in *BRAF*-mutant melanoma

**Fig. 4:**
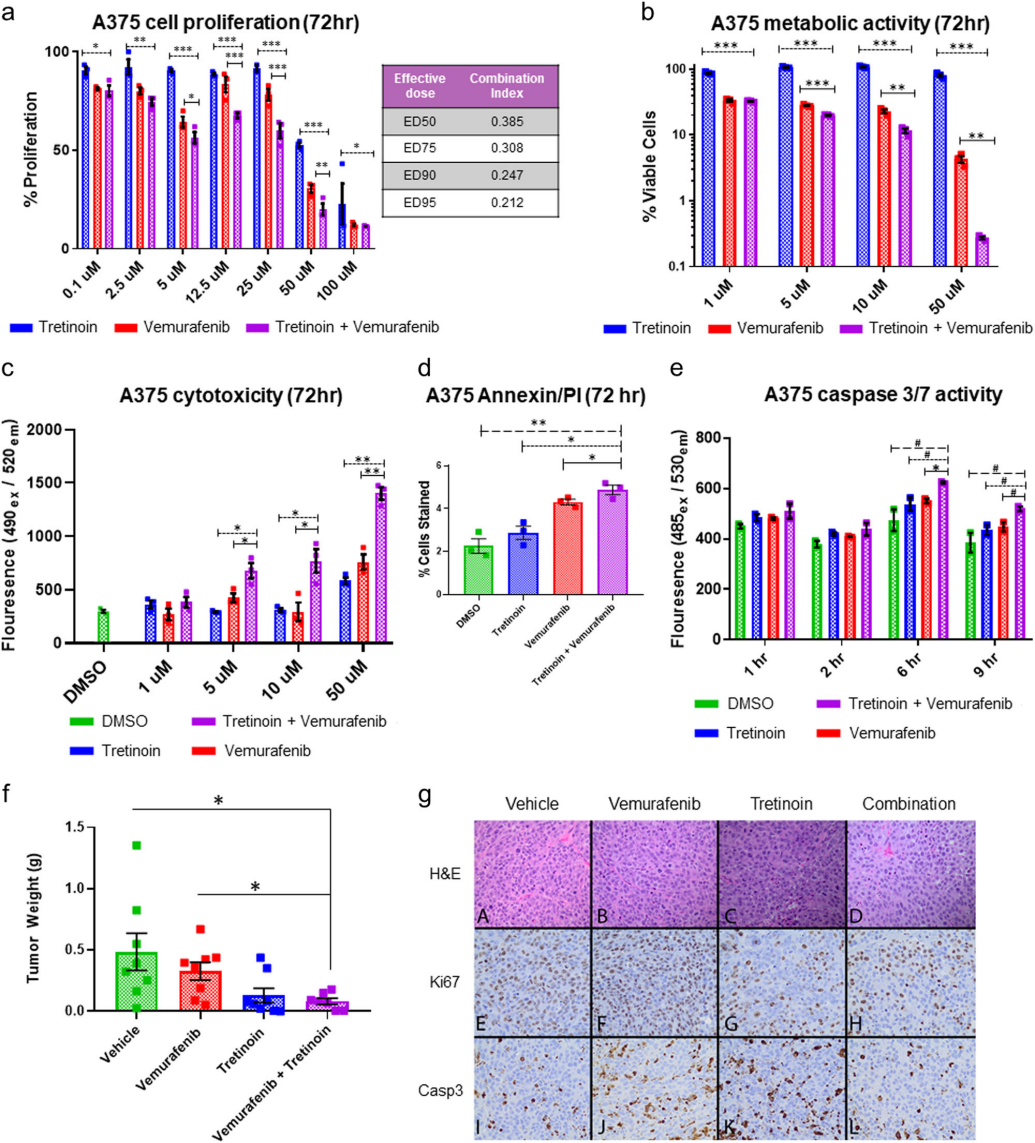
In vitro and in vivo validation of vemurafenib + tretinoin combination in *BRAF*-mutant melanoma models. **a** Percent proliferation of A375 cells following 72 h of treatment with tretinoin (blue), vemurafenib (red), and tretinoin + vemurafenib combination (purple) relative to dimethyl sulfoxide (DMSO) vehicle treatment as determined by MTS assay. Combination index (CI) values calculated by the Chou–Talalay method for drug combination synergy are reported for effective doses ED50, ED75, ED90, and ED95 values. **b** Percent viable A375 cells following 72 h of treatment with tretinoin (blue), vemurafenib (red), and tretinoin + vemurafenib combination (purple) relative to DMSO vehicle treatment quantified by ATP luminescence. **c** Cytotoxicity was measured in A375 cells via fluorescent cyanine dye bound to DNA released following cell death at 72 h following treatment with vehicle control (DMSO 1 μM), tretinoin (blue), vemurafenib (red), and tretinoin + vemurafenib combination (purple). **d** A375 cells were treated with 5 μM of DMSO (green), tretinoin (blue), vemurafenib (red), or tretinoin + vemurafenib combination (purple) for 72 h and stained for Annexin V and propidium iodide (PI). Cell populations were analyzed for apoptosis via flow cytometry and quantified with FlowJo software and shown as the mean for double-positive Annexin V/PI-stained cells. **e** Apoptosis was measured by caspase-3/7 enzymatic activity via a fluorescence based assay at 1, 2, 6, and 9 h time intervals following treatment (1 μM) with DMSO vehicle control (green), tretinoin (blue), vemurafenib (red), and tretinoin + vemurafenib combination (purple). **f** A375 cells were injected subcutaneously (1 × 10^6^ cells) into 8-week old athymic nude mice. After 10 days of tumor growth, mice were randomized to the following treatment groups (8 mice/group): daily oral gavage (6 days/week) with vemurafenib (50 mg/kg daily), tretinoin (10 mg/kg), combination or vehicle (20% PEG-400 (v/v) + 5% TPGS (v/v) + 75% ddH_2_O). Treatment concluded after 15 days, and tumors were harvested and weighed. **g** Representative images are shown for hematoxylin (H&E) (top; **a**–**d**) and immunohistochemical staining for Ki67 (middle; **e**–**h**) and caspase-3 (bottom; **i**–**l**) from formalin-fixed tumors resected from mice on day 15 of treatment. Error bars represent SEM. Significance was determined using unpaired *t* tests: ^#^*P* < 0.10, *P < 0.05, **P < 0.005, and ***P < 0.0005

Currently MAPK pathway inhibitors, including BRAF inhibitors (vemurafenib, dabrafenib) and MEK inhibitors (trametinib, cobimetinib), are the only mutation-specific, targeted therapies currently approved for melanoma patients. However, patients become rapidly resistant to these drugs, and more durable drug combinations are needed to combat resistance. Therefore, we were interested in pursuing drugs predicted in combination with BRAF inhibitors for prospective validation for *BRAF*-mutant melanoma. The top 10 drug combinations associated with BRAF inhibitors predicted for *BRAF*-mutant melanoma tumors are shown in Table 1. Interestingly, the top drug prediction involving a BRAF inhibitor ranked by our method for both the TCGA and GEO *BRAF* networks was the combination of vemurafenib and tretinoin (ATRA). This combination was not predicted for any other melanoma genomic subtype, as vemurafenib and dabrafenib were only returned for the *BRAF* networks. Therefore, we selected this drug combination for prospective validation in the A375 *BRAF*-mutant melanoma cell line. Notably, A375 cells treated with the combination of vemurafenib and tretinoin exhibited significantly lower proliferation following 72 h of vemurafenib treatment compared with either drug alone or vehicle control (Fig. 4a). To assess if these observations indicated a synergistic effect for the combination of vemurafenib + tretinoin, we employed the Chou–Talalay method to model synergy. Calculated combination index (CI) values were as follows for the following effective doses (ED): ED50 (0.385), ED75 (0.308), ED90 (0.247), and ED95 (0.212), and median CI value for all tested doses (0.369), where a CI value <1 indicates a synergistic interaction. To confirm these findings, we quantified the amount of ATP present in A375 cells following drug treatment at 72 h, indicating the number of metabolically active viable cells present. We observed that the combination of vemurafenib and tretinoin significantly decreased cell viability as compared either drug alone (Fig. 4b).

**Table 1:** Top 10 drug combination predictions for *BRAF*-mutant melanoma

*BRAF* primary melanoma (GSE15604)			*BRAF* primary melanoma (TCGA SKCM)		
Rank	Drug 1	Drug 2	Rank	Drug 1	Drug 2
1	Vemurafenib	Tretinoin	1	Vemurafenib	Tretinoin
2	Vemurafenib	Etoposide	2	Vemurafenib	Estradiol
3	Vemurafenib	Dinoprostone	3	Vemurafenib	Etoposide
4	Vemurafenib	Calcitriol	4	Vemurafenib	Bosutinib
5	Vemurafenib	Doxorubicin	5	Vemurafenib	Calcitriol
6	Vemurafenib	Amitriptyline	6	Vemurafenib	Capsaicin
7	Vemurafenib	Fluticasone	7	Vemurafenib	Decitabine
8	Vemurafenib	Dasatinib	8	Vemurafenib	Diazoxide
9	Vemurafenib	Bosutinib	9	Vemurafenib	Fludarabine
10	Vemurafenib	Celecoxib	10	Vemurafenib	Olopatadine

Drug combinations were generated using signaling networks from BRAF co-mutated root genes and gene expression data from two sources of *BRAF*-mutant melanoma (GSE15605 and TCGA SKCM)

*TCGA* The Cancer Genome Atlas, *SKCM* skin cutaneous melanoma

Additionally, we sought to determine if this drug combination could induce cell death in addition to arresting cell growth. We observed that combination treatment significantly increased A375 cell cytotoxicity as compared to either drug alone and vehicle control (Fig. 4c). Next, A375 cells were evaluated for the presence of apoptotic cells via annexin V-propidium iodide flow cytometry after 72 h of treatment with each drug alone, in combination and vehicle control. We observed that treatment of the A375 cells with the combination of vemurafenib and tretinoin led to a significant increase in these markers of cell death, as indicated by the double-positive annexin V/PI cell staining (Fig. 4d). Finally, we assessed the level of caspase-3/7 activity at different time intervals applicable with the assay protocol employed. We observed peak enzymatic activity at 6 h following treatment. We observed that the combination treatment marginally increased caspase-3/7 activity at 6 and 9 h post-treatment as compared to either drug alone or vehicle control (Fig. 4e).

To confirm the efficacy of the combination treatment in vivo, we pursued testing in melanoma mouse xenograft models. Of note, we did not achieve a stable solubility of tretinoin or vemurafenib in phosphate-buffered saline (PBS), and administered drugs via oral gavage in a vehicle solution of 20% PEG-400 (v/v) + 5% TPGS (v/v) + 75% ddH_2_O, as this resulted in an improved solubility and represents the clinically relevant route. Although previous in vivo studies of tretinoin have tested doses from 10 to 20 mg/kg in mice,^37–39^ we observed side effects of weight loss and dehydration at 10 mg/kg after one week of treatment. Therefore, we established 10 mg/kg to be the maximum tolerated dose of tretinoin. We also chose the maximum tolerated dose of 50 mg/kg for vemurafenib for a treatment period of 2 weeks that we have shown to be efficacious in A375 mouse xenograft models previously.^40^ To create melanoma mouse models, 11-week old athymic nude mice were inoculated with the A375 melanoma cell line (1.0 × 10^6^ cells suspended in PBS) via subcutaneous injection, and tumors were grown to 1000 mm^3^. Mice were randomized to drug treatment groups (*n* = 8 mice per group). After tumors were grown to sufficient size 10 days following injection, mice were treated via oral gavage daily (6 days/week) for 15 days with vemurafenib alone (50 mg/kg), tretinoin alone (10 mg/kg), vemurafenib + tretinoin combination, or vehicle control. As is shown in Fig. 4f, mice treated with the combination treatment showed a significant reduction in tumor weight compared to vemurafenib alone (*P* = 0.010, unpaired *t* test) and vehicle control (*P* = 0.029, unpaired *t* test). We also found that treatment with tretinoin alone led to a significant decrease in tumor weight as compared to vehicle control (*P* = 0.046, unpaired *t* test), while vemurafenib treatment alone did not lead to a significant reduction in tumor weight (*P* = 0.360, unpaired *t* test). As we found that mice treated with tretinoin exhibited known adverse effects of dehydration, these results should prompt future studies to test lower doses of tretinoin for models of melanoma in vivo. Histological analysis of tumors showed no observable difference across treatment groups regarding the degree of fibrosis, vascularity, or inflammation (Fig. 4g). Decrease in proliferation marker Ki67 was observed in tumors resected from mice treated with combination and tretinoin, as compared to vemurafenib or vehicle-treated mice. Interestingly, in contrast to the early peak in capsase-3 activity observed at 6 h in combination-treated cells in vitro, we observed a decrease in caspase-3 staining at day 15 in the combination-treated mouse tumors compared to either drug alone. Recent studies have shown that melanoma cells induce caspase-3 to promote cell survival and growth when exposed to cytotoxic therapy,^41^ as well as nonapoptotic roles of basal caspase-3 to promote migration and invasion of melanoma cells.^42^

### Mechanism of action predictions for vemurafenib + tretinoin combination in *BRAF*-mutant melanoma

**Fig. 5:**
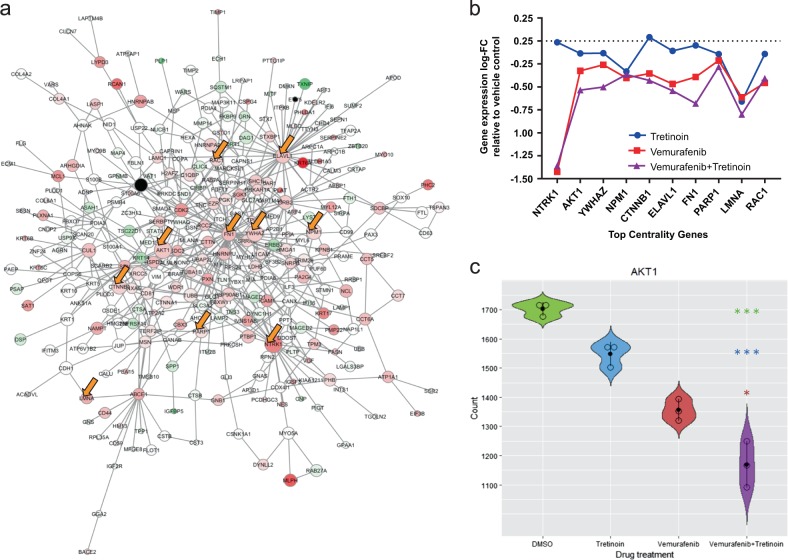
Vemurafenib + tretinoin combination decreases gene expression in the *BRAF*-melanoma network. **a** Network visualization of original *BRAF* network with gene expression log-fold change (false discovery rate (FDR) <0.05) from RNA-sequencing (RNA-seq) analysis of combination-treated A375 cells relative to vehicle control-treated A375 cells superimposed to scale color-coding of gene nodes (green: positive fold change; red: negative fold change; white: weakly changed; black: not significantly differentially expressed). Yellow arrows denote top 10 centrality genes differentially expressed following combination treatment. **b** Top centrality genes ordered by highest centrality score (left) to lowest centrality score (right) with corresponding log-fold change for differential expression status of each gene following combination treatment relative to vehicle control. **c** Normalized RNA-seq gene expression count plot is shown for *AKT1* (V-Akt murine thymoma viral oncogene homolog 1), a top centrality genes in the network, in response to each drug treatment condition. The differential expression status of the combination treatment group relative to other treatment groups are color-coded as follows: green = DMSO vehicle control; tretinoin = blue; vemurafenib = red; *adj. *P* < 0.05 and ***adj. *P* < 0.0005

To investigate the potential mechanisms of action of the combination of vemurafenib and tretinoin in the context of *BRAF*-mutant melanoma, we performed RNA-sequencing (RNA-seq) analysis of A375 melanoma cells following treatment with vemurafenib alone, tretinoin alone, vemurafenib + tretinoin combination, and vehicle control in triplicate. We observed that global gene expression patterns clustered samples according to treatment group using three complementary dimensionality reduction techniques hierarchical clustering with Euclidean distance, principal components analysis, and multi-dimensional scaling, as shown in Figure S10. Next, we performed differential gene expression analysis for several comparisons, including each drug treatment group relative to vehicle control, as well as the drug combination group relative to each single drug alone. For each comparison, we calculated log 2 fold changes and defined differential expression significance as adjusted *P* < 0.05 (Wald test; see Supplemental Methods for details). We next sought to determine if the gene expression patterns produced by the vemurafenib + tretinoin combination treatment could (i) reverse those of the overall network structure in the *BRAF* melanoma subnetwork (*n* = 306 genes) and (ii) specifically reduce the highly central (i.e., topologically important) genes. To do so, we mapped differentially expressed genes in the combination treatment A375 cells relative to vehicle control onto the original *BRAF* melanoma network and observed that the majority (65%) of network genes were down-regulated (Fig. 5a).

Next, we sought to determine if the genes exhibiting the highest centrality were preferentially altered by the drug combination treatment compared to either drug alone or vehicle control. Table S3 lists the top genes ranked by overall centrality score within the network as well as the top 10 genes connected to either vemurafenib or tretinoin ranked by centrality score. All top centrality genes significantly differentially expressed (adj. *P* < 0.05) in the combination treatment group were down-regulated. We then compared the differential expression status of these genes ranked by centrality score in A375 cells treated with tretinoin alone, vemurafenib alone, and combination (Fig. 5b). Overall, the combination treatment showed a trend of decreased expression of the top centrality genes relative to either drug alone. As an example, we visualized a gene count plot for *AKT1* (V-Akt murine thymoma viral oncogene homolog 1), as this gene overlapped between both sets of genes ranked by centrality and has a known role in melanoma tumorigenesis (Fig. 5c). Interestingly, *AKT1* is the top-ranked gene showing the highest centrality of genes connected to and exhibited the second highest centrality score in the overall network. We found that the combination treatment significantly reduced the expression of *AKT1* relative to vehicle control and either vemurafenib or tretinoin alone. We also found that high messenger RNA (mRNA) expression of *AKT1* was associated with poor survival in melanoma patients in several studies (Figure S11).

To determine whether the decrease in gene expression is a specific effect of the drug treatment on the network genes, rather than due to a global decrease in gene expression, we visualized the distribution of positive and negative fold change in MA (mean difference) and histogram plots for each drug treatment comparison, as well as the total number of mapped reads (gene counts) across each sample (Figure S12). We observed a uniform number of gene counts in each sample across treatment groups and even distribution of fold-change values indicating a balanced number of up- and down-regulated genes. In fact, a slightly greater number of genes were significantly increased in each drug treatment condition, suggesting that the decreased gene expression observed in the *BRAF* melanoma network genes is likely due to biological causes rather than an artifact of widespread suppression of gene expression.

Finally, we examined differences in gene expression of the direct targets of vemurafenib and tretinoin following drug treatment. Although the *BRAF* gene was included in the *BRAF* melanoma network, no significant difference in gene expression was observed relative to vehicle control for either vemurafenib, tretinoin, or combination (Figure S13). This finding was unsurprising, as the known mechanism of vemurafenib involves the selective binding, and therefore blocking, of the active state of the BRAF kinase domain responsible for its constituent activation only when mutated at the V600 position, rather than exerting regulatory effects on endogenous mRNA expression levels. However, we observed that the drug combination treatment significantly altered the expression of each of the known isoforms of the retinoic acid receptor (RAR) (*n* = 3) and retinoid X receptor (RXR) (*n* = 3) genes. It is known that increased expression of these receptors results in increased responsiveness of cancer cells to the growth arresting and differentiating effects of tretinoin, and that tretinoin treatment can further increase expression of these receptors. Interestingly, we found that vemurafenib and the combination of vemurafenib + tretinoin could increase the expression of RAR-β, RAR-γ, RXR-α, and RXR-β, while the expression of RAR-α and RXR-γ were significantly reduced by vemurafenib and vemurafenib + tretinoin combination treatment. Notably, the effects of tretinoin are thought to be predominantly mediated via the RAR-β2 isoform. Remarkably, the RAR-β gene was the only RAR/RXR gene observed to be significantly increased by the combination treatment as compared to vemurafenib alone, tretinoin alone, and vehicle control (Figure S13), suggesting that synergistic effects of vemurafenib + tretinoin we observed may be due to, in part, a favorable increase in RAR-β gene expression.

## Discussion

In this study we applied SynGeNet, a computational drug combination prediction method, to four subtypes of melanoma based on genomic classification of major driver events, including mutations in *BRAF*, *NRAS*, *NF1*, and TWT tumors. We employed this systems-based approach to interpret the effects of genetic aberrations and drug treatments on genome-wide expression profiles and PPIs through integrative network models. Through the analysis of these networks, we further identified potential synergistic drug combinations based on a synergy model prioritizing drug combination candidates that maximally alter melanoma networks via reversal of gene expression and targeting topologically important network nodes. Finally, we validated our drug combination predictions through a combination of in silico and experimental approaches, including a drug repurposing candidate involving tretinoin in combination with vemurafenib for *BRAF*-mutant melanoma. Importantly, we prospectively validated in vitro the predicted mechanisms underlying the SynGeNet methodology.

We demonstrated that frequently co-mutated genes, transcriptomic profiles, and resulting networks distinguished the four melanoma genomic subtypes, and these differences were also reflected in the pattern of predicted drug combinations. Notably, our SynGeNet method achieved a high precision in predicting drug combinations tested in melanoma cell lines representing each of the genomic subtype and performed better than selecting random drug pairs from the same pool of FDA-approved drugs. This is an important aspect of rank-based prediction methods in biomedical applications, where a high density of true-positive predictions are prioritized toward the top of the list can be selected for subsequent validation due to time and cost restraints. We also established a broader relevance of drug combinations predictions identified across all subtypes, as we observed a high degree of literature associations for melanoma–drug and drug–drug pairs for subtype-specific predictions. Furthermore, we conducted several internal evaluations of our method’s assumptions and found: (1) drug combinations predicted for genomic subtype-specific networks in each context performed better than two examples of generalized melanoma networks, (2) the integrative network models combining genomic and transcriptomic data outperformed employing either data type alone, and (3) randomly re-wiring the interaction partners within the network reduced the true-positive and increased false-positive predictions, suggesting the network structure underlying the connections among proteins and interactions among potential drug combinations is an important aspect to our method’s performance. Interestingly, we observed the constructed subnetworks to be highly stable with respect to the removal of individual genes, including major driver genes (e.g., *BRAF*, *NRAS*, etc.).

For our prospective experimental validation, we focused on drug combinations involving BRAF inhibitors for *BRAF*-mutant melanoma, as this represents the standard of care for these melanoma patients. Among our top-ranked drug combination pairs involving BRAF inhibitors, several drug combinations were previously validated in preclinical and clinical studies, including celecoxib (COX-2 nonsteroidal anti-inflammatory),^43^ dasatinib (Src family kinase inhibitor),^44^ and decitabine (cytotoxic chemotherapy).^45^ In this study, we demonstrated experimental evidence validating the drug combination of vemurafenib and tretinoin (ATRA), which was the top-ranked prediction involving BRAF inhibitors for *BRAF*-mutant melanoma by our method. We demonstrated synergy in suppressing cell proliferation and cell viability, as well as increasing cytotoxicity and cell death in vitro across a range of equal ratios of drug doses. Surprisingly, we observed in vivo that tretinoin alone and in combination with vemurafenib could significantly reduce tumors, despite its relatively weaker effects in vitro compared to vemurafenib alone or in combination. Importantly, we also experimentally validated the reversal of overall gene expression of subnetwork nodes as well as those produced by the highly central and most influential genes in the *BRAF* network following drug combination.

Tretinoin (ATRA) is the most biologically active metabolite of vitamin A (retinoid), and functions in the regulation of cell development, differentiation, and proliferation. ATRA has been used as a tumor differentiation therapy, which aims to reprogram cancer cells to inhibit proliferation, trigger cell cycle arrest, inducing apoptosis, and restore normal cell characteristics. ATRA is the first-line therapy for acute promyelocytic leukemia (APL), and can induce complete remission in these patients.^46^ Retinoids have been used as chemotherapeutics and in the adjuvant setting in a variety of cancers; however, ATRA is less effective in treating solid tumors, which may be due to its reduced aqueous solubility limiting sufficient quantities delivered to the tumor sites.^47^ Reports on the effectiveness of ATRA therapy in melanoma have also been conflicting. ATRA has been shown to inhibit growth of normal human melanocytes, while its effectiveness in melanoma cell lines was shown to be minimal.^48^ In a recent in vivo study, topical tretinoin inhibited B16F10 melanoma growth via promoting the maturation and cytotoxic capabilities of anti-tumor CD8+ T cells in mice.^49^ A large-scale retrospective analysis of 69,635 patients enrolled in the VITAL study revealed that baseline use of retinol supplements, as well as intake of high-dose retinol supplementation (>1200 μg/day), was associated with significantly reduced risk of melanoma.^50^ Another group applied a network-guided approach to predict sensitivity to ATRA based on gene expression profiles from a wide array of tumor types contained in the TCGA database. Interestingly, they found that uveal melanoma was the neoplasia with the highest predicted sensitivity.^51^ Additionally, a phase II clinical trial is currently investigating ATRA in combination with ipilimumab as a treatment for stage IV melanoma (NCT02403778).

Effects of ATRA are mediated by nuclear receptors, including RARs (RAR-α, RAR-β, RAR-γ) and RXRs (RXR-α, RXR-β, RXR-γ), which are also expressed as different mRNA isoforms. While the precise cause of resistance to ATRA in melanoma is not completely understood, one mechanism may be low expression of RAR-β2. In fact, RAR-β2 expression is repressed primarily by DNA methylation in a variety of cancers, and it has been reported that its promoter is methylated in a 30–70% of melanoma cell lines and clinical samples.^52^ Interestingly, ectopic expression or induced expression of endogenous RAR-β2 restores sensitivity to retinoic acid. One study also demonstrated that basal levels of RAR-β2 in melanoma cell lines were correlated with the ability of ATRA to reduce cell proliferation, and that ATRA treatment could further increase RAR-β2 expression and sensitivity to ATRA. The authors did not observe correlations between other RA or RX receptor gene expression and responsiveness to ATRA in this study, although protein expression was not examined. We also observed variable effects of tretinoin in this study. When given alone, tretinoin was minimally effective in vitro, but had dramatic effects in reducing tumor volume alone when given to nude mice, which also have compromised adaptive immune systems. An interesting finding in our study was that treatment with vemurafenib and the combination of vemurafenib + tretinoin significantly increased expression of RAR-β, RAR-γ, RXR-α, and RXR-β and reduced expression of RAR-α and RXR-γ, which may promote responsiveness to tretinoin in these melanoma cells.

Our RNA-seq analysis of single drug- and combination-treated melanoma cells revealed several interesting findings. We found that the vemurafenib + tretinoin combination treatment suppressed gene expression of the majority of *BRAF* melanoma network genes that were highly up-regulated in patient tumors. We also observed that seven out of ten of the top most central genes in the network were significantly down-regulated following combination treatment. Interestingly, we found that *AKT1*, the second highest central gene overall and top most central gene of those connected to either drug, was significantly down-regulated following combination treatment as compared to vehicle or either drug alone. Remarkably, another recent network-based study of melanoma also reported that *AKT1* was the highest ranking “hub” gene by PageRank centrality and showed the highest degree of differential gene expression in patient tumors.^53^ The activation of the PI3K-AKT pathway has an established role in melanoma. In fact, it has been shown that increased expression of AKT1 and activation of AKT1 via phosphorylation promotes melanoma proliferation and metastasis,^54,55^ is associated with reduced melanoma patient survival,^56,57^ and mediates resistance to BRAF inhibitor therapy.^58,59^ The observed reduction of AKT1 expression via treatment with vemurafenib + tretinoin combination may partially explain the drug combination’s synergistic action in reducing cell proliferation in *BRAF*-mutant A375 cells in this study.

Another interesting finding in our analyses was the potential role of *FN1* (fibronectin 1) in melanoma and a candidate mechanism by which the combination of vemurafenib and tretinoin may mediate its effects. *FN1* was the only other gene in addition to *AKT1* that was ranked among both the top 10 most highly central genes in the network and top centrality genes interacting with vemurafenib or tretinoin. High expression of FN1 has been associated with tumorigenesis and metastasis in a variety of solid cancers, and suppression of *FN1* has been linked to reduced cancer cell proliferation and increased apoptosis.^60–65^ Several studies have also demonstrated that down-regulation of *FN1* expression could be mediated by microRNAs, which led to tumor suppression in different cancers.^66–68^ The role of *FN1* in melanoma has not been well established, although two studies reported that *FN1* was linked to the hypoxic microenvironment of melanoma in promoting an invasive tumor phenotype.^69^

## Limitations and future directions

Our study was limited in several ways, and future studies should seek to overcome these limitations. While we employed genomic and transcriptomic data from the largest WES cutaneous melanoma cohort study to date from the TCGA, we nevertheless are subject to cohort bias in the primary melanoma tumor samples analyzed from the TCGA (*n* = 100) in addition to transcriptomics data from a smaller-scale GEO dataset (*n* = 16). For instance, a subsequent WGS melanoma study that included additional non-cutaneous melanoma tumors recently identified several other significantly mutated potential driver genes missed by the TCGA study.^70^ In another WGS study of desmoplastic melanomas, *NF1* was found to be highly mutated and lacked the most common hotspot mutations in *BRAF* or *NRAS* were discovered in this cohort.^71^ Furthermore, the mutation rate of known melanoma driver mutations is also influenced by body site location of tumors and ultraviolet exposure, leading to intra- and inter-tumor differences.^72^ We also restricted our analysis of genomic aberrations to gene mutations in coding regions. This may have biased our results considering that the TWT melanomas exhibited the highest proportion of structural rearrangements, including large DNA segment amplifications and deletions.^27^ While the use of genomic and transcriptomic from primary melanoma tumors permitted a more focused analysis on oncogenic pathways, other factors beyond the cancer cell, including microenvironment and metastatic tumors should be considered.

To construct protein subnetworks, we relied upon a comprehensive curated database of PPIs from the BioGRID database (*n* = 1,168,521 current non-redundant interactions). However, validated directionality information for these interactions was not readily available on a large scale, and thus our protein subnetworks were constructed as undirected graphs integrating gene expression information and PPIs. Due to this limitation, we excluded LOF gene mutations from our analysis, as network flow was mapped to include “positive” interactions with higher weights applied to PPIs with a high degree of evidence and up-regulated gene expression fold-change values. Nevertheless, future work should model the impact of LOF mutations and down-regulation of signaling pathways using well-validated, directional PPI information. Biological and drug interaction databases are not complete and contain biases to the most well-studied genes. Therefore, highly studied genes may lead to an overestimation of connectivity within a network and an inflated number of drug and disease associations. Although our network analyses were repeated with different datasets and in random permutations, further iterations could confirm the reproducibility of the results. Utilizing networks with higher resolution information, including directed edges, isoform-specific interactions, and variations of protein structures impacting PPI and protein–drug interactions could improve results limited to undirected networks in this study. Furthermore, conditional dependencies and feedback loops characterizing gene regulatory relationships could be explicitly modeled, such as the relationship between transcription factors and the expression patterns of genes under their influence. Incorporating data from melanoma patient tumors that can be implicated in the upstream or downstream regulation of gene expression, including methylation, microRNA, and protein expression profiles, may also improve the accuracy of these models. Additional causal genetic data could be integrated with existing drug information in future studies. For example, one recent study integrated synthetic lethality screens and gene set enrichment analysis to identify synergistic drug combinations for colorectal cancer, one of which was validated in vitro and in vivo PDX models^73^ and is being pursued in clinical trials.^74^ Additionally, while we evaluated several clustering algorithms to distinguish drug communities based on similarities of gene expression profiles, other sources of drug-related data types could be compared to determine optimal features that classify drugs on mechanistic differences. Alternative statistical methods for connectivity mapping could also be evaluated in the context of this drug combination prediction approach.^75,76^ Future work could also investigate hypotheses formulated on opposing principles outlined in this study to define antagonism, for example, enhancing gene expression, weakly affecting or promoting the function of highly central genes in disease networks. However, a recent study comparing different computational drug combination prediction methods showed that predicting synergy did not correlate with the ability to predict antagonism.^77^ Thus, future work should continue with careful consideration to specific goals in predicting drug combinations.

One significant clinical challenge with the use of ATRA therapy is differentiation syndrome, which causes severe adverse effects due to endothelial activation, cytokine release, and other vascular factors mediating tissue damage. In this study, we observed several adverse dermatological effects, including skin dehydration and flakiness in mice treated with 10 mg/kg of tretinoin daily via oral gavage. We also observed dose-dependent weight loss in mice treated with tretinoin at daily doses of 10–20 mg/kg. One recent study tested a formulation of a liposome encapsulated ATRA in comparison to free ATRA in a mouse model of lung metastasis using tail vein-injected mouse melanoma cell line B16F10.^47^ Compared to free ATRA, the authors observed that encapsulated ATRA achieved an increased lifespan, and reduced lung tumor nodules and tumor markers at a low dose (0.60 mg/kg per day), while also reducing several unwanted side effects of ATRA therapy, including reducing oxidative stress, lipid profiles, increasing T-helper type 1 (Th1) cytokines, and decreasing Th2 cytokines. Given these findings, the results of this study also warrant further testing of tretinoin in melanoma at lower doses through other delivery systems to reduced unwanted side effects.

Important avenues to explore in future studies including modeling drug toxicities and adverse interactions to balance with synergy predictions, as unexpected side effects are a common cause for failure in clinical trials of drug combinations. For example, one group recently proposed a signed drug-target network to jointly model synergy and adverse effects based on the proportion of on- and off-target effects, respectively.^78^ Another group showed how the similarity of toxicity profiles could be used to model drug–drug interactions.^79^ Furthermore, a publicly available as part of the NIH LINCS platform has recently been made available for researchers to predict side effects of drugs using the L1000 transcriptomics data.^80^ As recent advances in immune-based check point inhibitors have shown improved clinical benefits to melanoma patients and in other cancers, developing methods to systematically predict drug combinations to improve the efficacy of existing immunotherapies represents an exciting opportunity for future research applications. For instance, a recent study systematically mapped gene expression signatures characterizing drug treatments and immune cell types to model pharmacological interactions with immune system components.^81^ Specific to tretinoin (ATRA) as a drug repurposing candidate in melanoma, it is known that ATRA can also mediate its effects by modulating different components of the immune system and free radical oxidizing species,^47,49,82^ and these mechanisms and corresponding biomarkers should be investigated in future in vivo and clinical studies. Finally, our method highlights the potential to personalize drug combination predictions for melanomas classified into four major genomic subtypes, and it will be important to evaluate this precision medicine paradigm in pan-cancer analyses along similar genomic-based groups as well as other diverse molecular classifications.

## Conclusions

Overall, the results of this study add to the growing body of evidence supporting the use of systems-based medicine frameworks for drug discovery applications. Given the high financial and labor costs to screen large sets of pairwise drug combinations, these approaches will be particularly beneficial as a preclinical hypothesis generation system to reduce the search space of drug candidates and provide insights into potential mechanisms via extensive model simulations under different conditions. We also highlight the potential to better understand the pathophysiology of complex disease via global analysis of networks and molecular profiles. We present experimental evidence for the top drug combination predicted by our method for *BRAF*-mutant melanoma and validate gene expression at the network level and for highly ranked centrality genes. From a translational and clinical standpoint, our work highlights the potential to personalize drug combination predictions for diseases classified according to specific molecular contexts.

## Methods

### Data sources

Gene expression data of primary melanoma tumor samples was obtained from GEO dataset GSE15605 (*n* = 46) and of primary melanoma tumor samples from TCGA SKCM dataset (*n* = 100). Melanoma-associated genomic variant data was obtained from the DisGeNET (DGN) database (v5.0)^83^ and TCGA SKCM dataset from the cBioPortal database.^84,85^ PPI data was obtained from the BioGRID database.^86^ Gene expression profiles (*Z*-scores) of 633 FDA-approved drugs tested in vitro in the A375 melanoma cell line was obtained from LINCS L1000 database.^87^ Drug target interaction data was obtained from DrugBank (v5.0)^88^ and high confidence interactions (score >700) from STITCH (v4.0).^89^ Drug classes were mapped using the KEGG DRUG database.^90^ Literature abstracts containing melanoma–drug associations and drug–drug associations were obtained from the PubMed database.

### Genomic variant data analysis

The cBioPortal web tool (http://www.cbioportal.org/) used to define melanoma patient cohorts were defined according to *BRAF*, *NRAS*, and *NF1* mutation status and generate gene mutation plots from the TCGA SKCM dataset. For each genomic sub-group defined for primary melanoma tumors in the TCGA SKCM dataset (*BRAF*, *NRAS*, *NF1*, and TWT), we selected co-occurring mutated genes that exhibited a log odds ratio >0 with a corresponding Fisher's exact test *P* ≤ 0.05. Significantly mutated genes and LOF mutations were determined using the InVex method, where we used *P* ≤ 0.05 as a significance threshold.^91^ Gene–disease association scores for genes associated with melanoma were obtained from the DGN database, which were ranked according to the number and type of evidence as previously described. For the GSE15605 dataset, *BRAF* and *NRAS* mutation status of each tumor were quantified by reverse transcription polymerase chain reaction.

### Analysis of publicly available gene expression data

Microarray gene expression data from primary melanoma tumor from GEO dataset GSE15605 and RNA-seq gene expression data from primary melanoma tumors from the TCGA SKCM dataset were selected for analysis. For Affymetrix Human Genome U133 Plus 2.0 microarray data from dataset GSE15605, RMA- and quantile-normalized gene expression data was log 2 transformed, and gene expression values of probesets mapping to the same gene were averaged. Of note, snap-frozen melanoma tumors from this study were evaluated by a dermatopathologist who identified areas with >70% tumor cellularity, as described previously.^92^ Differential expression analysis was performed using the *limma* R package for tumor vs. normal samples in each of the available genomic sub-groups (*BRAF*+, *NRAS*+, and *BRAF*/*NRAS* double wild type). Log-fold-change values were calculated, and statistical significance of differential expression was set as false discovery rate <0.05 (Benjamini and Hochberg adjustment). RNA-seq data from TCGA SKCM (2016_01_28 version) was downloaded via Firebrowse (http://firebrowse.org/), and RSEM-normalized gene expression values from each of the genomic sub-groups (*BRAF*, *NRAS*, *NF1*, and TWT) were mapped to differentially expressed genes as defined above. Hierarchical clustering of normalized gene expression data was performed in the R statistical environment using the heatmap.3 function with the Euclidean distance of genes. The top 10% of genes with the largest variance were selected, and heatmap plots were generated using the *gplots* and *GMD* R packages.

### Generation of genotype-specific protein subnetworks

Protein subnetworks were generated for each unique combination of gene expression data from each dataset and from significantly co-mutated genes observed in the TCGA SKCM dataset for each of the four genomic sub-groups of primary melanoma tumors. In addition, two general melanoma networks were constructed using melanoma-associated genes from the DGN and from significantly mutated genes observed across all melanoma tumors (SMG). The belief propagation algorithm was used to construct the protein subnetworks by determining network flow originating from co-mutated “root” genes connected through PPIs in a background network from the BioGRID database.^36^ Network flow was mapped via the belief propagation algorithm by favoring connected genes with up-regulated fold-change values and disfavoring protein–protein connections with low experimental evidence to maximize targetable molecular entities. Of note, mutated genes resulting in LOF were not included as root node genes in any network, as connected interactions in the undirected network were predicated on the presence functional proteins. Constructing each network can be described as a subnetwork inference problem mathematically as follows: given the BioGRID background network, *G* = (*V*, *E*), the subnetwork, *G*′ = (*V*′, *E*′), is constructed to minimize the cost function (Eq. 1):

$$\mathop {{\min }}\limits_{E^\prime \subseteq E,V^\prime \subseteq V} \mathop {\sum }\limits_{e \in E^\prime } c_e - \lambda \mathop {\sum }\limits_{i \in V^\prime } b_i,$$

where *c*_*e*_ represents the cost of an edge and *b*_*i*_ represents the gene expression fold change for network nodes. The *λ* parameter regulates the tradeoff between the *c*_*e*_ and *b*_*i*_ parameters and thus the overall size of the subnetwork. Here we set *λ* = 0.025 based on empirical evaluation. Network visualizations were created using Cytoscape software (v 3.6.0).^93^

### Computational drug combination prediction

We computed drug combination predictions using networks generated for each set of root genes and gene expression data in a multi-step method referred to as SynGeNet.^34^

The Kolmogorov–Smirnov statistic was used to calculate connectivity scores between gene expression profiles (at the level of *Z*-scores) of 633 FDA-approved drugs tested melanoma cells from the LINCS L1000 transcriptomics database and gene expression profiles representing the up-regulated genes in the melanoma disease networks. Individual drugs were ranked by negative connectivity scores, that is, those drugs corresponding to a “reversal” of the melanoma disease network gene signature.^8^ Connectivity scores were normalized to a range of [−1, 1], and we selected those drugs with normalized connectivity scores ≤−0.50. The selected drugs were empirically prioritized using weights as follows (Eq. 2):

$$w_i = \left( {1.0 + \left( {1.0 - r_i/n_{\mathrm{d}}} \right)} \right),$$

where *w*_*i*_ and *r*_*i*_ are the weight and rank of the *i*th selected drug and *n*_*d*_ is the number of selected drugs. Using drug target information from the DrugBank and STITCH databases, we further filtered drugs to include those with targets in the network. Next, drug target genes were mapped on the constructed melanoma disease networks, and the centrality of each drug target gene within the overall disease network was calculated using the betweenness, closeness, and page-rank centrality metrics from the *igraph* R package. The closeness of a network node calculates the average length of the shortest path between the node and all other nodes in the graph. Betweenness determines the number of times a node acts as a bridge along the shortest path between two other nodes. The page-rank metric quantifies the number of connections (edges) for a given node and weights the connecting edges by the degree of the originating nodes. Drug synergy scores were calculated for drug pairs (*d*_*i*_ and *d*_*j*_) using the weighted connectivity scores for each drug and the weighted sum of the network centrality parameters for the combined set of drug target (*cs*_*t*_) in the network as follows (Eq. 3):

$$s_{ij} = w_i \times w_j \times \mathop {\sum }\limits_t cs_t.$$

Drug combinations were ranked by synergy score in decreasing order.

### Drug community clustering

Drug community clustering analyses were performed on the Pearson's correlation matrix of drug-induced gene expression profiles. The following R packages were used to employ the corresponding clustering methods: *apcluster* package apcluster function (affinity propagation); *fpc* package, pamk function using *k* = 10 clusters based on an optimum average silhouette width tested on a range of *k* values 1:100 (partitioning around medoids); *hclust* package hclust function (hierarchical clustering); *dbscan* package dbscan function an epsilon neighborhood size = 3 and minPts = 5, per method recommendations (density-based clustering of applications with noise). Clustering algorithms were compared using the cluster.stats function from the *fpc* R package. We restricted final drug combination candidates to drug pairs from different drug communities. Drug pair classes were visualized using circos plots generated from the *circlize* R package.

### Combinatorial drug screening validation dataset

We obtained results from a recently published high-throughput drug combination screening study evaluating 5778 drug combinations among 108 drugs.^35^ We utilized Bliss synergy scores for drug pairs tested four cell lines representing each of the major genomic subtypes of melanoma: *BRAF* mutant (A375), *NRAS* mutant (IPC-298), *NF1* mutant (MeWo), and TWT (COLO792). Synergistic drug combinations corresponded to those with positive Bliss scores, as defined by the authors of this study. We evaluated all genotype-specific drug combination predictions overlapping with those evaluated in the corresponding representative cell line. True positives and false positives were determined as predicted drug predictions with positive and negative Bliss scores, respectively. False negatives corresponded to drug combinations with positive Bliss scores identified in the screening study that were missed by our method despite both single drugs present in our original pool. We then calculated the precision, recall, and corresponding F1 scores for each set of genomic subtype-specific drug combination predictions.

## Experimental methods

Methods for in vitro and in vivo experiments, including RNA-seq analysis and statistics sections, may be found in Supplemental Methods.

### Ethics statement

Access to melanoma patient data via TCGA and GEO repositories for research purposes were permitted via IRB approval from corresponding study sites. Mouse studies were conducted according to the policies and protocols set by the ULAR at The Ohio State University.

### Code availability

The R code for SynGeNet method and corresponding datasets have been made available as Supplemental material, as well as at the following link: https://figshare.com/articles/SynGeNet_Synergy_from_gene_expression_and_network_mining/7551296 (10.6084/m9.figshare.7551296). All code dependencies, instructions for download, and Apache 2.0 license details are included in the SynGeNet R package

## Supplementary information

Supplemental Material

Dataset 1

## Data Availability

All data are (i) made available in additional files or (ii) are obtained from publicly available databases and are cited accordingly. Raw and processed RNA-seq data generated in this study is available in the Gene Expression Omnibus: accession GSE109731.

## Supplementary information

**Supplementary information** accompanies the paper on the *npj Systems Biology and Applications* website (10.1038/s41540-019-0085-4).
