# Supplementary material for: Synergy from gene expression and network mining (SynGeNet) method predicts synergistic drug combinations for diverse melanoma genomic subtypes
Source: NPJ Syst Biol Appl. 2019 Feb 26;5:6. doi: 10.1038/s41540-019-0085-4 (PMC6391384; doi:10.1038/s41540-019-0085-4)
Supplement: Supplementary file 1 — Supplemental Material [file 41540_2019_85_MOESM1_ESM.pdf]

Supplemental Figures

Supplemental Figure S1. Signaling networks and respective drug combination predictions for four genomic subtypes of melanoma. A) Using RNAseq gene expression data from the TCGA SKCM database and protein-protein interactions from the BIOGRID database, signaling networks were generated using the belief propagation using co-mutated genes as root genes for each respective genomic subtype: *BRAF* mutant, *NRAS* mutant, *NF1* mutant and Triple wild type (TWT) melanoma. The size of the network nodes are weighted to the average centrality metrics and the node color is scaled to gene expression (green: up-regulated, red: down-regulated). B) Venn diagrams visualizing overlap among four genomic subtypes of melanoma for corresponding co-mutated root genes, signaling networks generated and drug combinations predicted using mutation and gene expression data from the TCGA SKCM dataset.

A

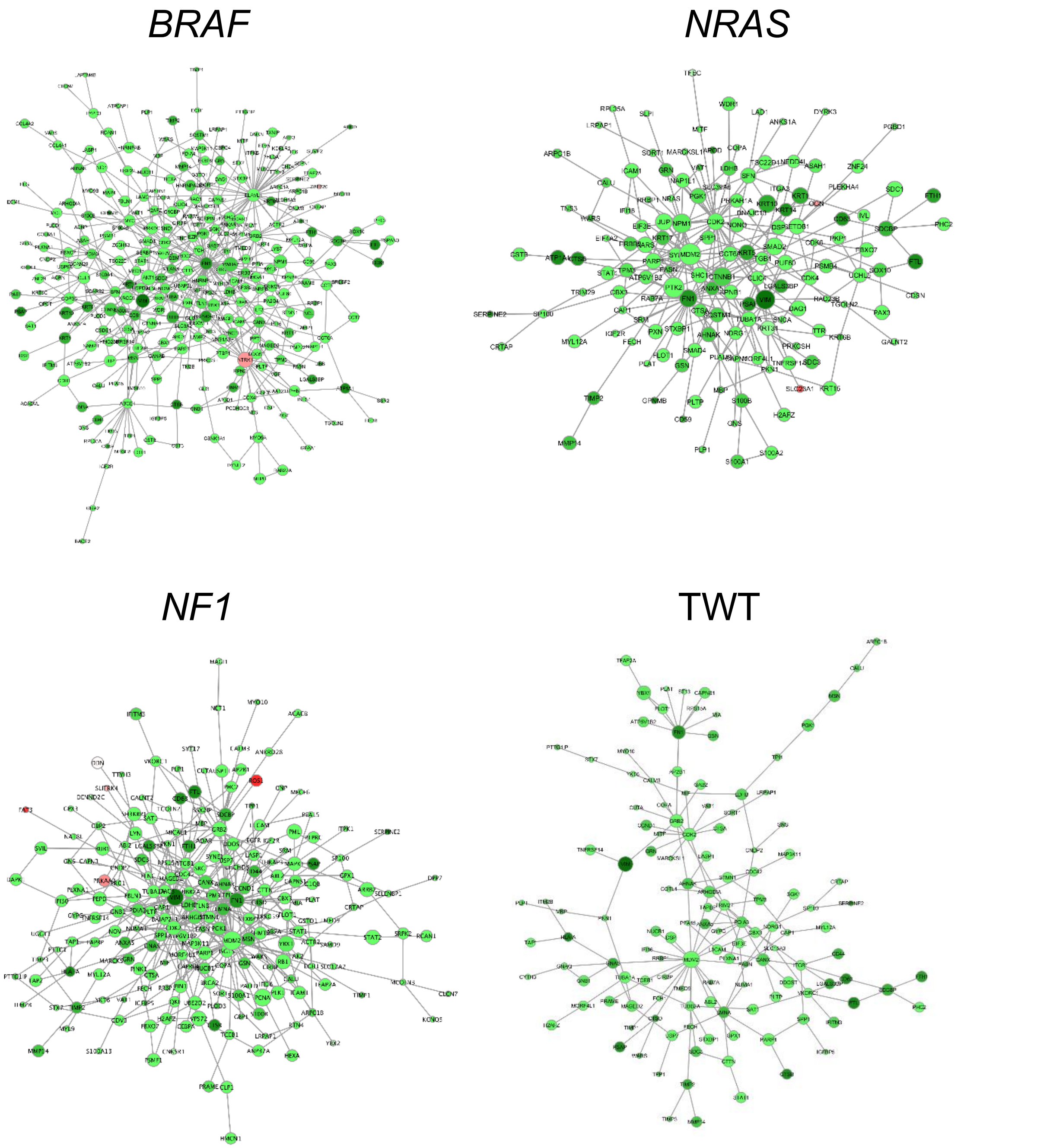

B

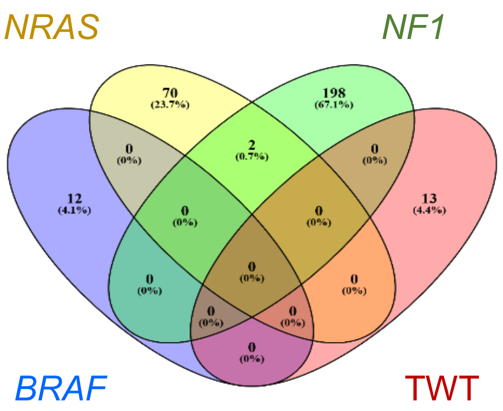

Root genes

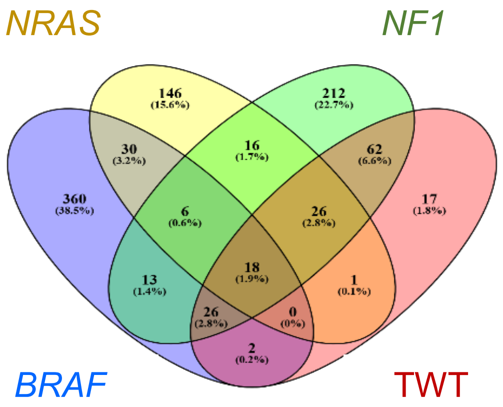

Signaling network interactions

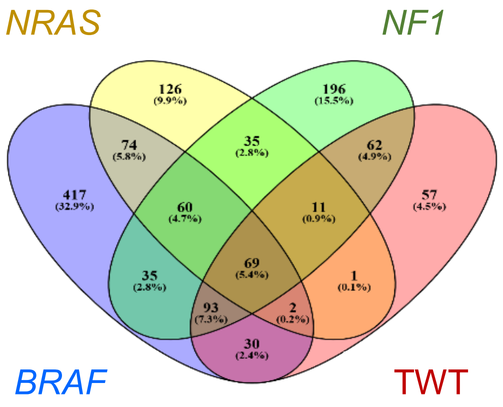

Drug combination predictions

Supplemental Figure S2. Overlap among signaling networks and drug combination predictions using melanoma primary tumor GEO dataset GSE15605. Venn diagrams visualizing overlap among four genomic subtypes of melanoma generated using the GEO GSE15605 for A) signaling network interactions and B) drug combination predictions. Venn diagrams visualizing overlap among four genomic subtypes of melanoma generated using the GEO GSE15605 for A) signaling network interactions and B) drug combination predictions.

A

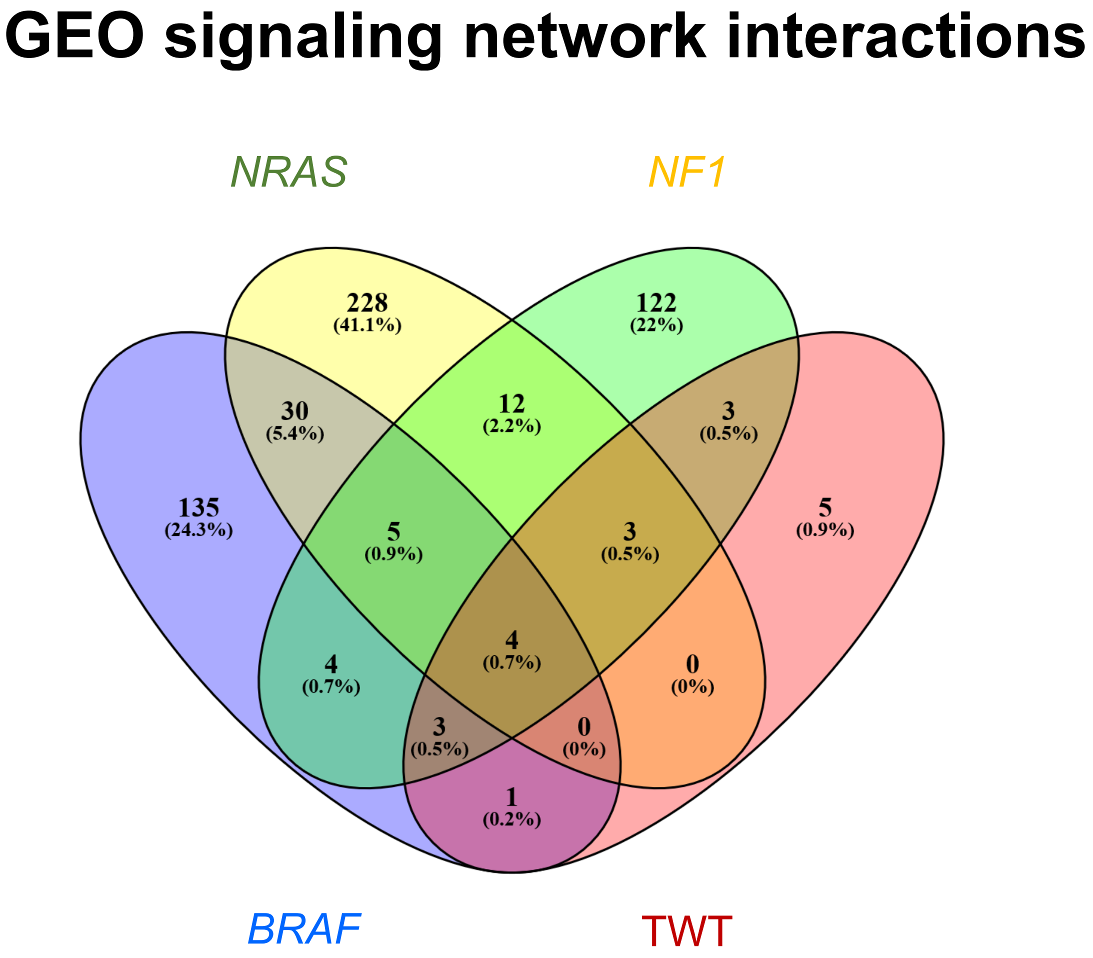

B

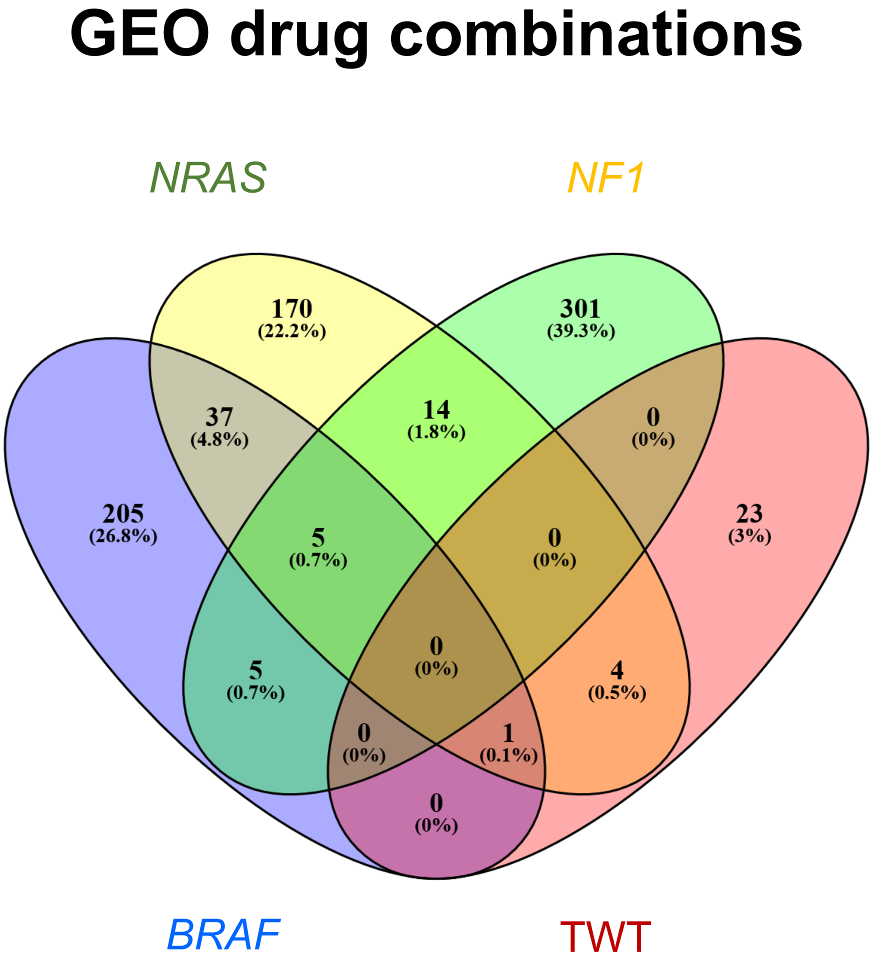
GEO drug combinations
NRAS
NF1
170
(22.2%)
301
(39.3%)
37
(4.8%)
14
(1.8%)
0
(0%)
205
(26.8%)
5
(0.7%)
0
(0%)
23
(3%)
0
(0%)
5
(0.7%)
4
(0.5%)
0
(0%)
1
(0.1%)
0
(0%)
BRAF
TWT

Supplemental Figure S3. Relationships between major drug class categories among predicted drug combinations for melanoma genomic subtypes. Constituent drugs in predicted drug combinations were mapped to drug class categories from the KEGG Drug database, and drug combination patterns among major drug classes are visualized via circos plots for each melanoma genomic subtype defined in the TCGA SKCM dataset for *BRAF* mutant (top left), *NRAS* mutant (top right), *NF1* mutant (bottom left) and Triple wild type (bottom right) melanoma.

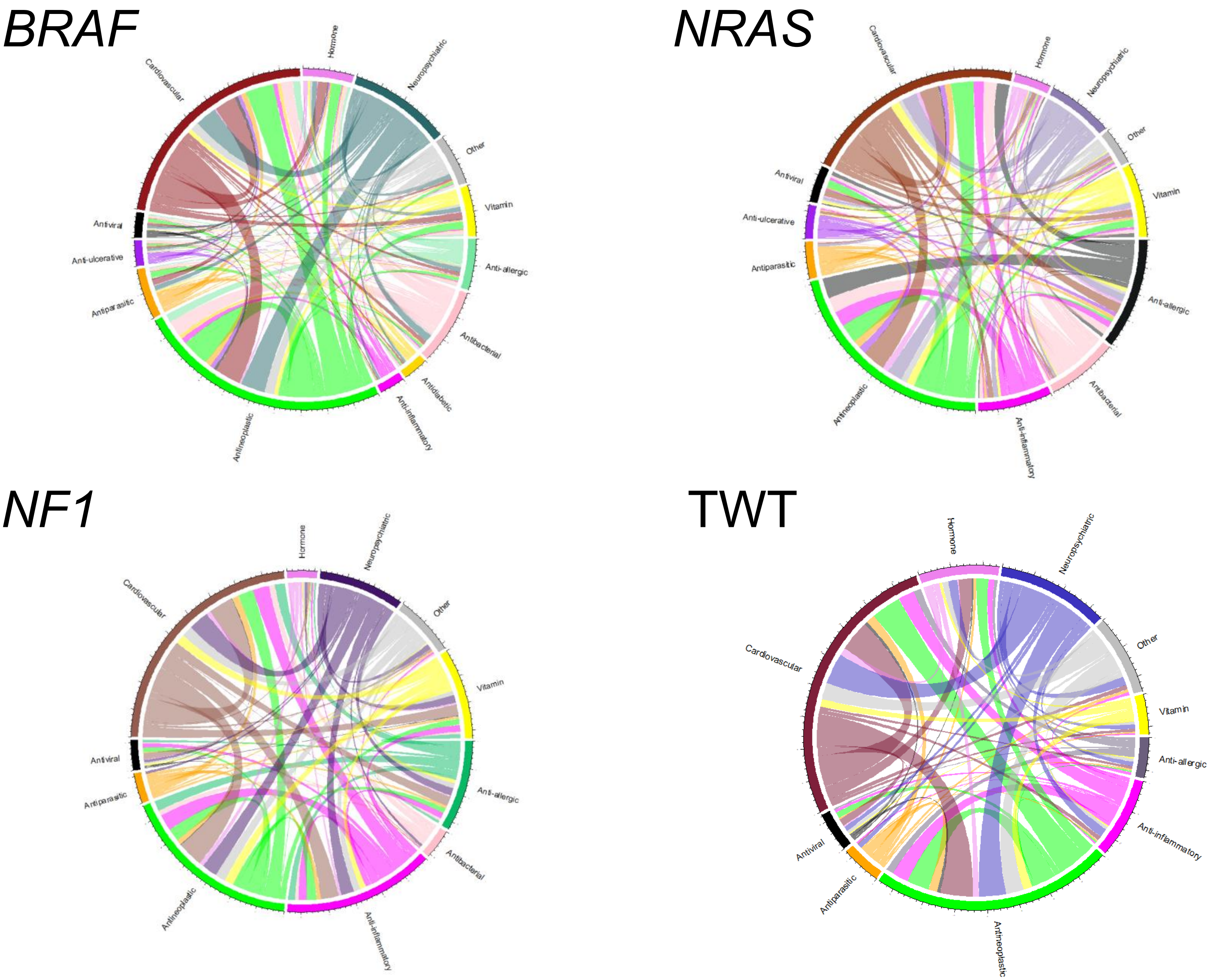

Supplemental Figure S4. Leave one out analysis to test signaling network size robustness. Signaling network sizes (number of protein-protein interactions) and percent overlap with the original network are shown for networks generated using two sources of melanoma root genes, including 28 melanoma-associated genes from the DisGeNet database (A and B, respectively) and 39 genes found to be frequently mutated in melanoma tumors from the TCGA SKCM database (C and D, respectively). Both networks used the same set of differentially expressed genes defined between all primary melanoma tumors vs. normal skin samples from GEO dataset GSE15605 as well as the same parameters for network construction using the belief propagation algorithm. The leftmost bar shows the resulting signaling network size for all root genes in both cases (Total), and each subsequent bar shows the effect of removing one root gene from either list. Of note, only two root genes with non-loss of function mutations are shared between the gene sets obtained from DisGeNet and TCGA SKCM databases, including BRAF and NRAS.

A

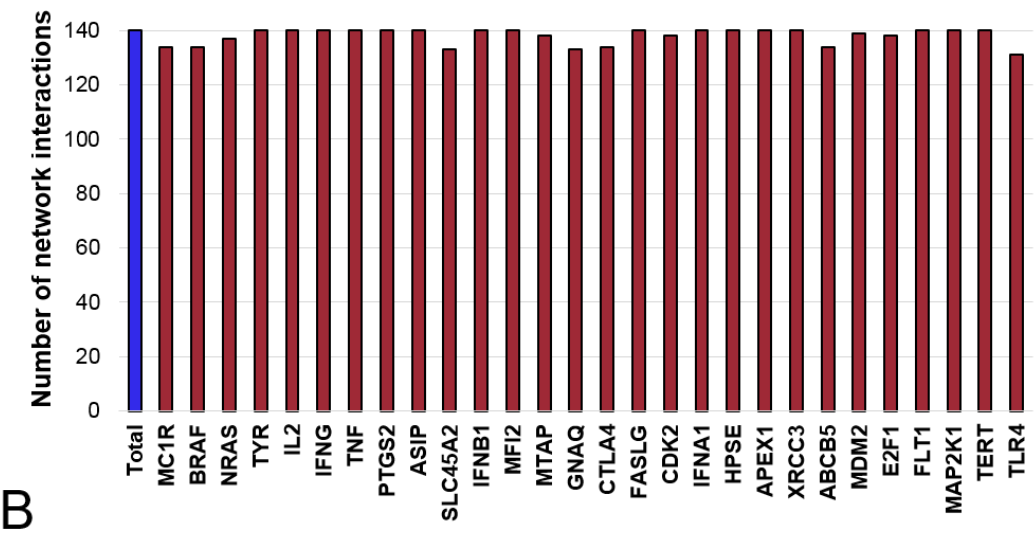

B

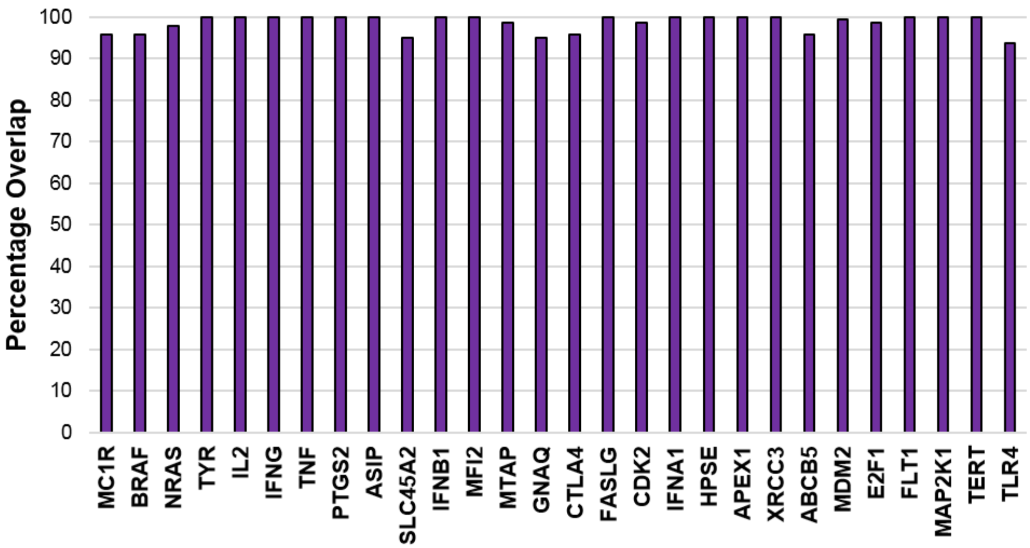

C

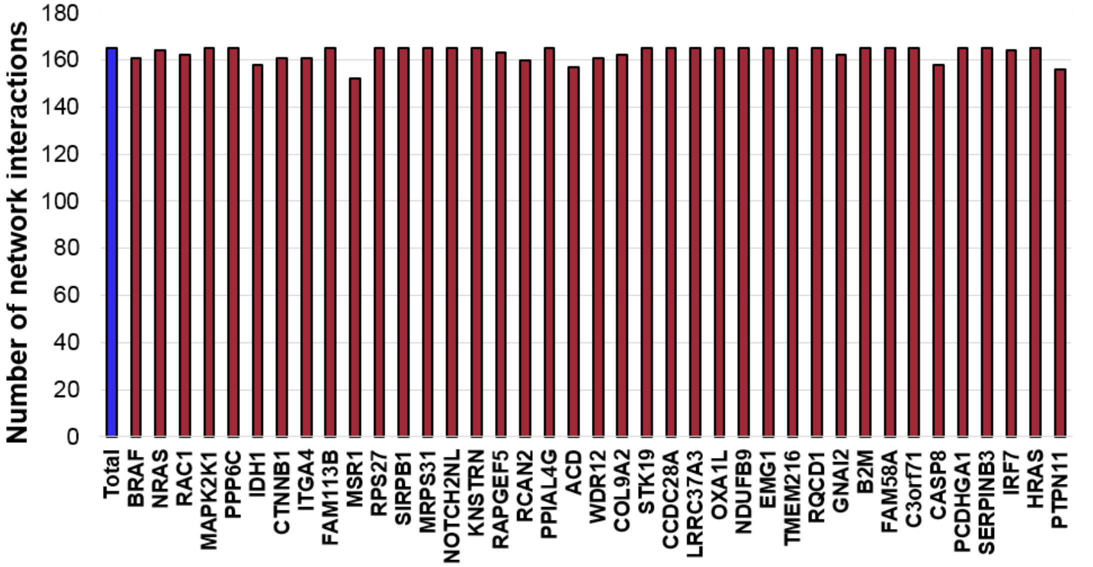
Number of network interactions
0
20
40
60
80
100
120
140
160
180
Total
BRAF
NRAS
RAC1
MAPK2K1
PPP6C
IDH1
CTNNB1
ITGA4
FAM113B
MSR1
RPS27
SIRPB1
MRPS31
NOTCH2NL
KNSTRN
RAPGEF5
RCAN2
PPIAL4G
ACD
WDR12
COL9A2
STK19
CCDC28A
LRRC37A3
OXA1L
NDUFB9
EMG1
TMEM216
RQCD1
GNAI2
B2M
FAM58A
C3orf71
CASP8
PCDHGA1
SERPINB3
IRF7
HRAS
PTPN11

D

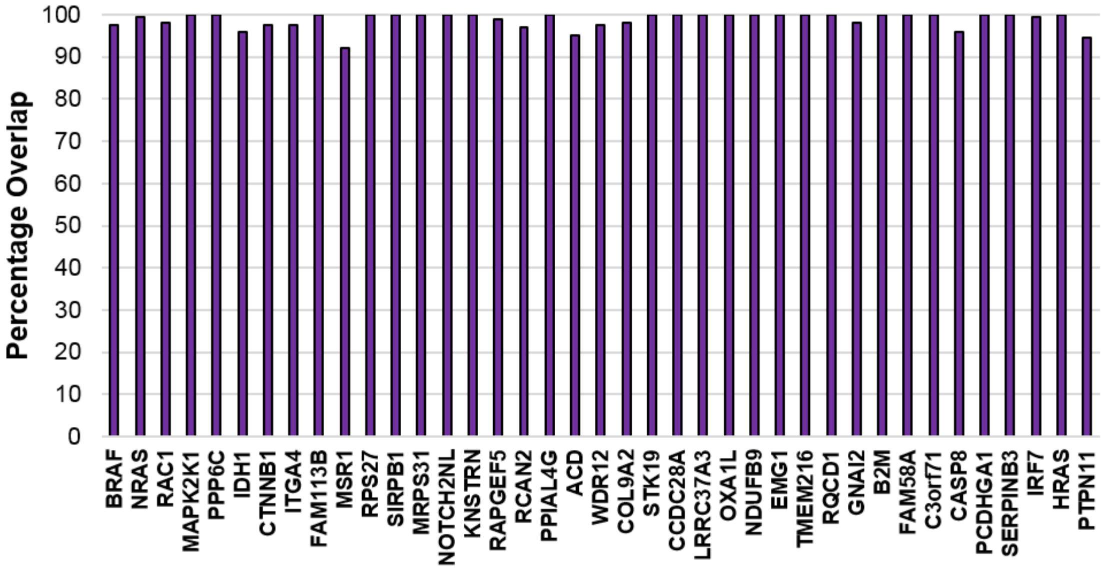
Percentage Overlap
0
10
20
30
40
50
60
70
80
90
100
BRAF
NRAS
RAC1
MAPK2K1
PPP6C
IDH1
CTNNB1
ITGA4
FAM113B
MSR1
RPS27
SIRPB1
MRPS31
NOTCH2NL
KNSTRN
RAPGEF5
RCAN2
PPIAL4G
ACD
WDR12
COL9A2
STK19
CCDC28A
LRRC37A3
OXA1L
NDUFB9
EMG1
TMEM216
RQCD1
GNAI2
B2M
FAM58A
C3orf71
CASP8
PCDHGA1
SERPINB3
IRF7
HRAS
PTPN11

Supplemental Figure S5. Comparison of drug community clustering algorithms. Each clustering algorithm was applied to a Pearson correlation matrix of drug-induced gene expression profiles for 633 FDA approved drugs in the LINCS database, including: affinity propagation (AP, n=37 clusters), partitioning around medoids (PAMK, n=10), hierarchical clustering (HC, n=26) and density based clustering of applications with noise (DBSCAN, n=4). Four internal measures were used to assess the performance of the clustering algorithms, three of which should be maximized (green): Average between cluster distance/within cluster distance (A); Dunn 2 index, which describes the minimum average dissimilarity between two clusters / maximum average within cluster similarity (B); Average silhouette width, estimates the average distance between clusters; and one that is sought to be minimized (orange): within cluster sum of squares (D).

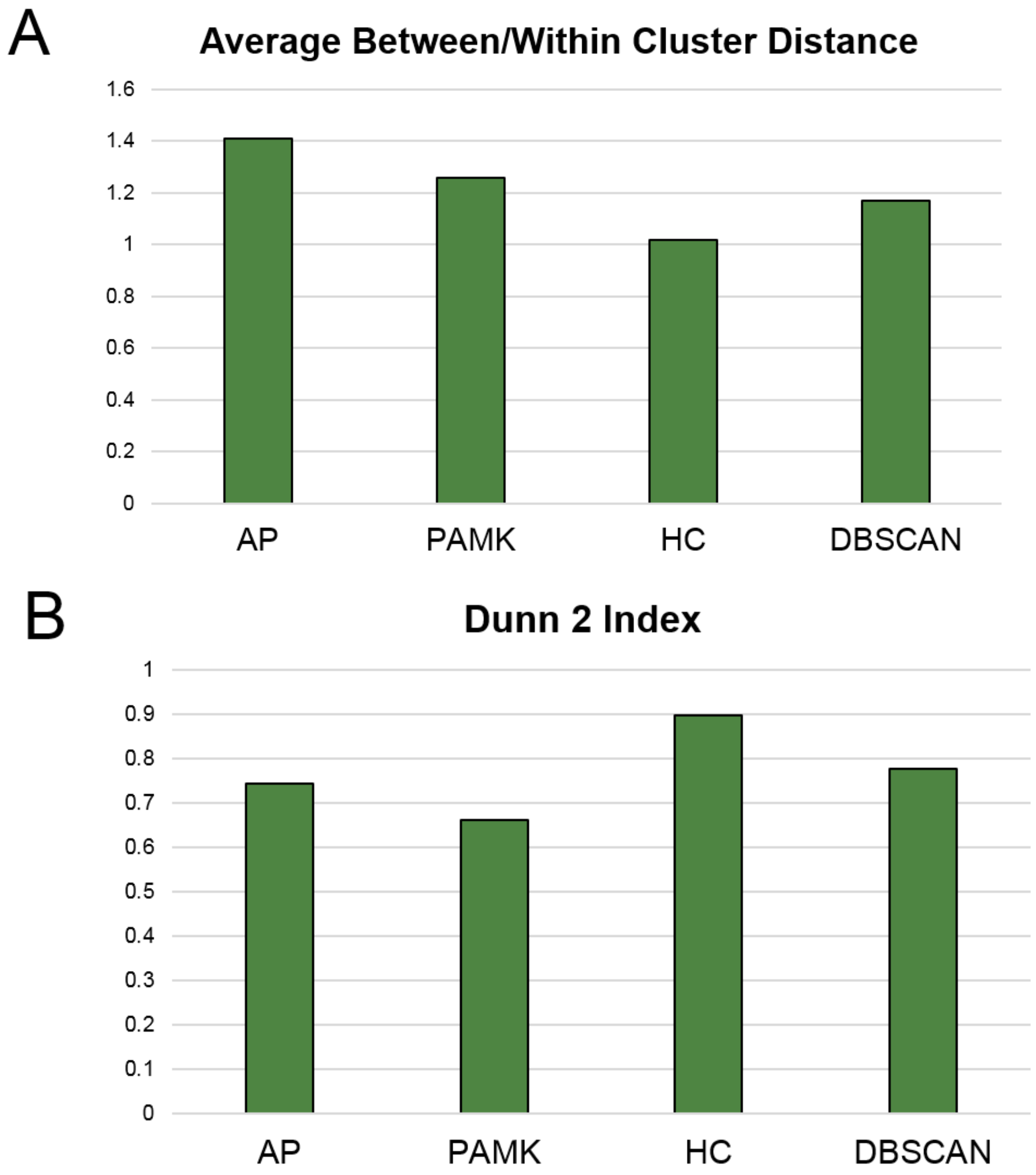

C

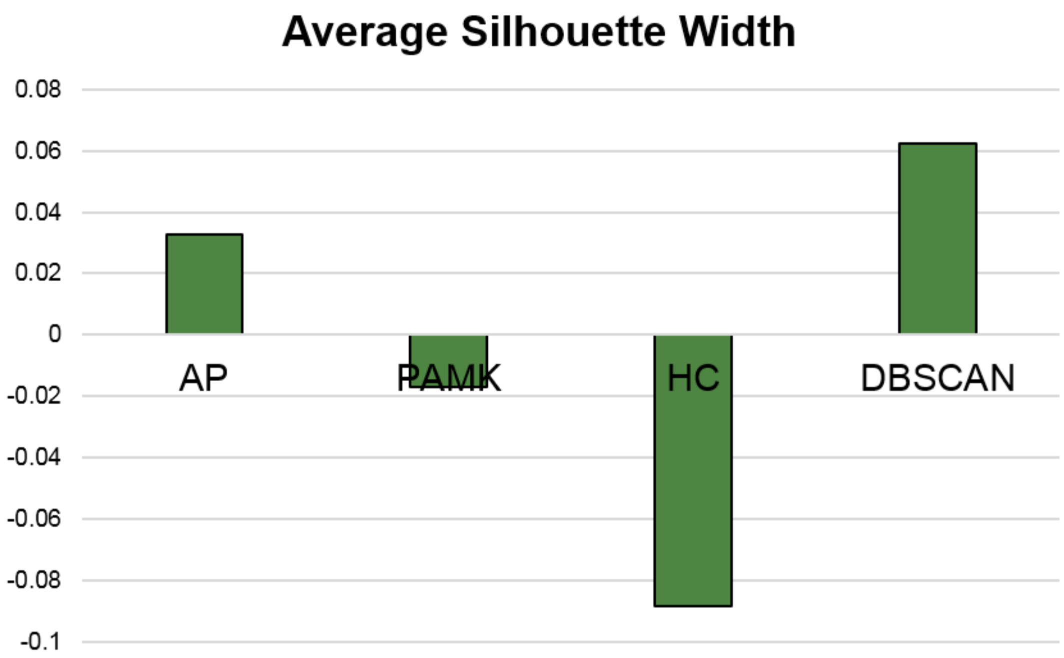
Average Silhouette Width
0.08
0.06
0.04
0.02
0
-0.02
-0.04
-0.06
-0.08
-0.1
AP
PAMK
HC
DBSCAN

D

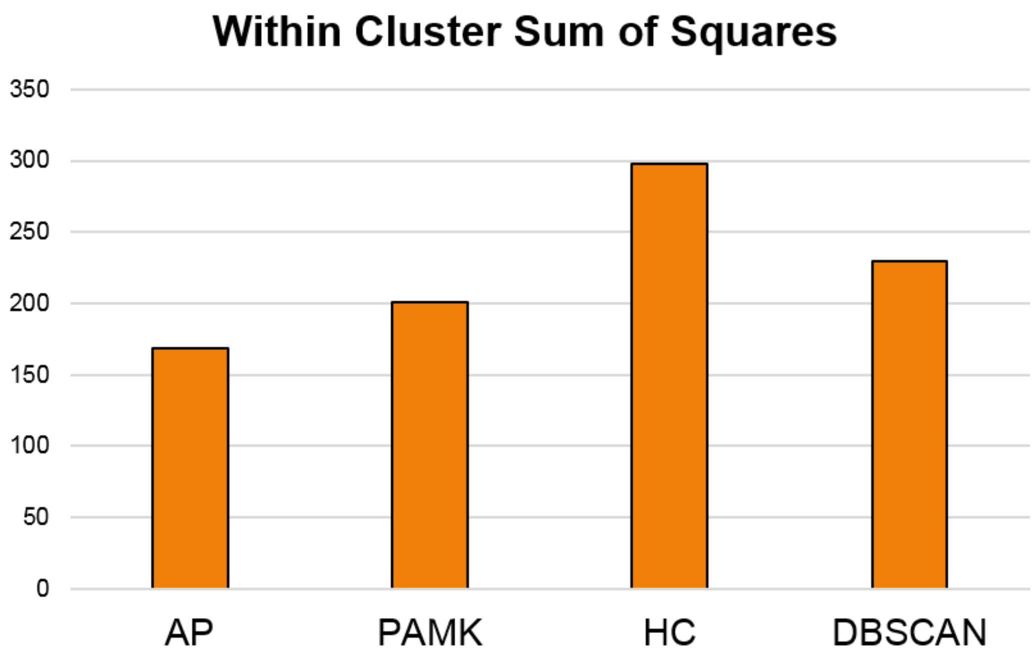
Within Cluster Sum of Squares
350
300
250
200
150
100
50
0
AP
PAMK
HC
DBSCAN

Supplemental Figure S6. Drug communities determined by AP clustering. Drug communities structures generated by the affinity propagation algorithm for two generalized melanoma networks using the DisGeNet dataset genes (A) and significantly mutated genes in the TCGA SKCM dataset (B) as the root gene sets to construct melanoma disease signaling networks.

A

DisGeNet melanoma drug community network

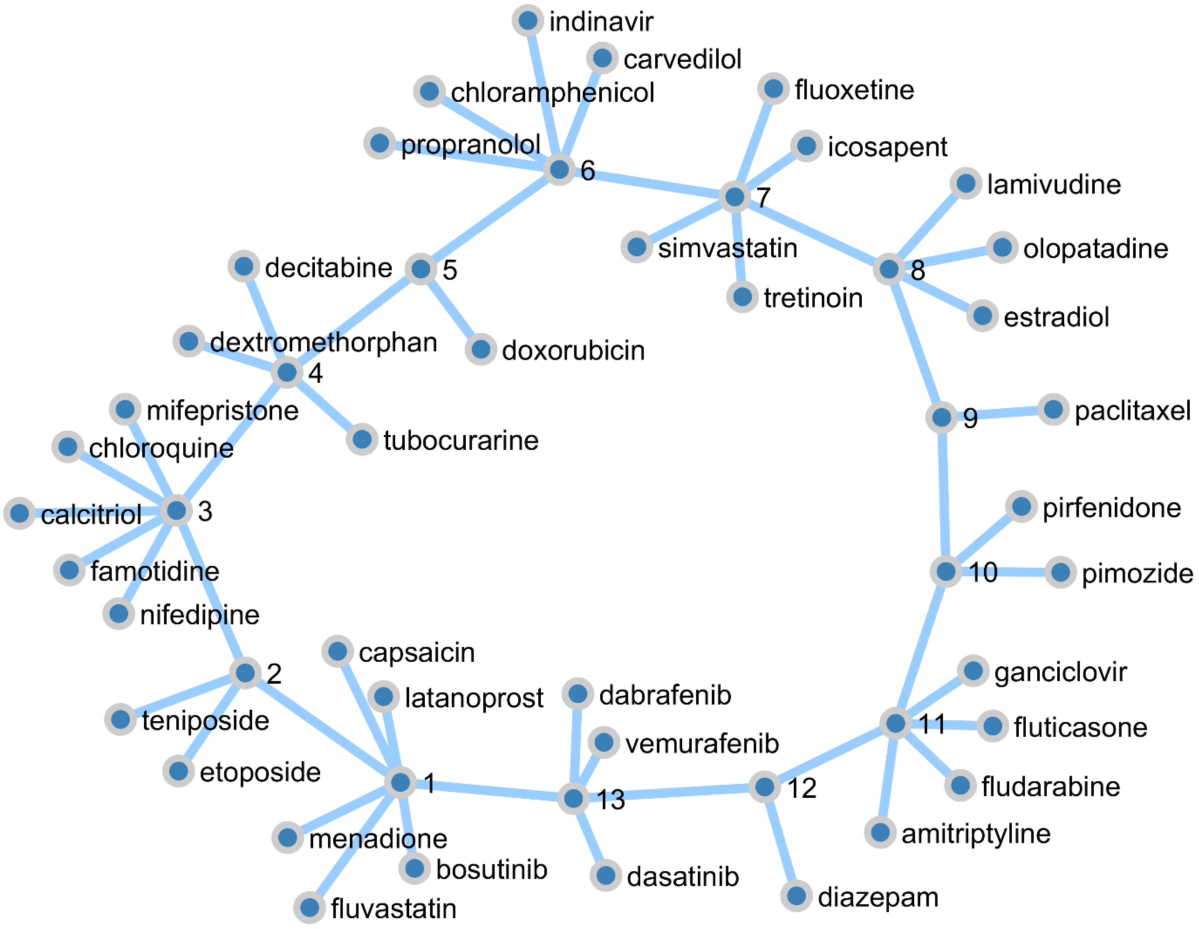

B

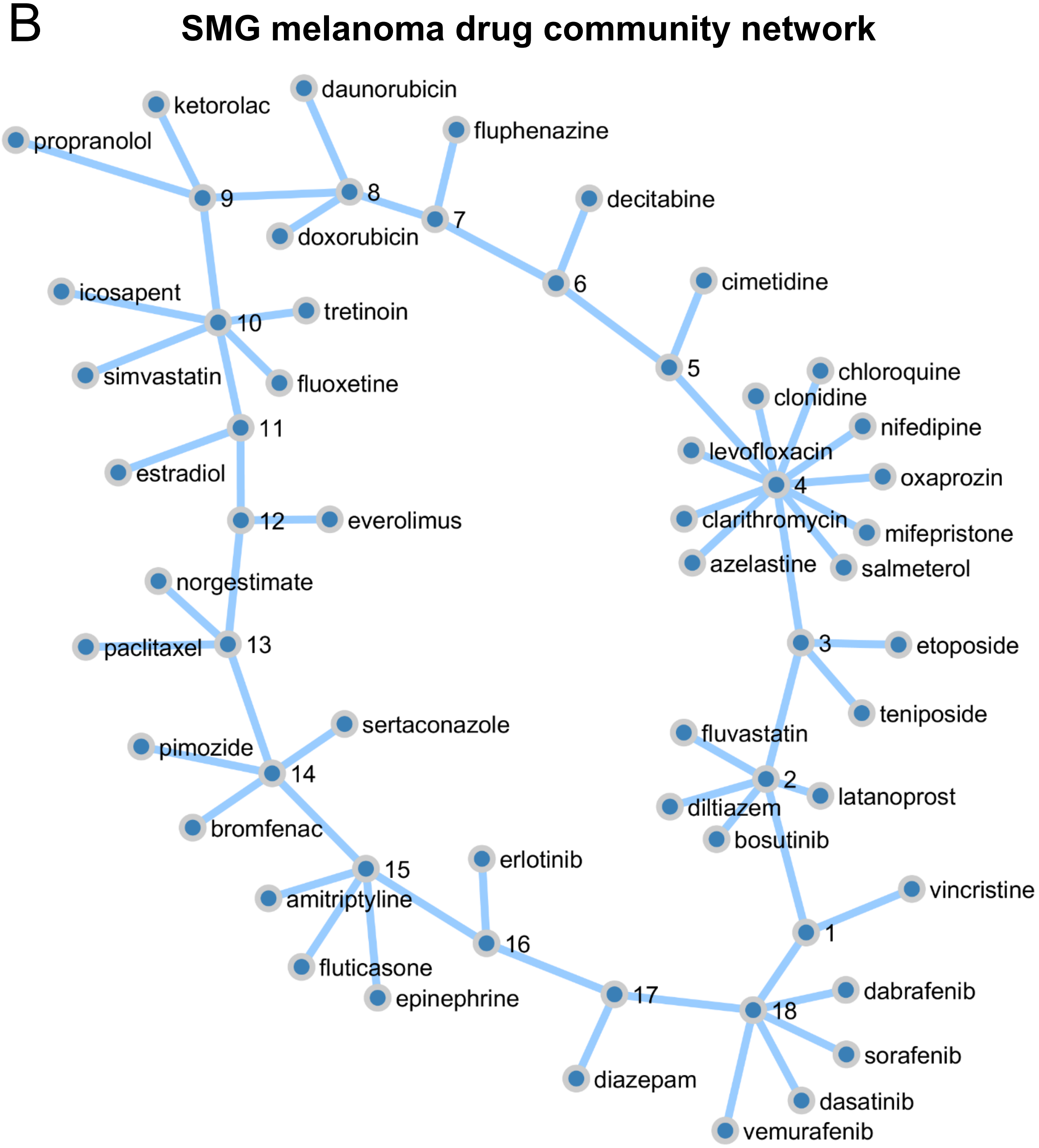

SMG melanoma drug community network
ketorolac
daunorubicin
propranolol
fluphenazine
9
8
7
doxorubicin
decitabine
6
cimetidine
icosapent
tretinoin
10
simvastatin
fluoxetine
5
chloroquine
clonidine
nifedipine
11
levofloxacin
estradiol
4
oxaprozin
12
everolimus
clarithromycin
mifepristone
azelastine
salmeterol
norgestimate
paclitaxel
13
3
etoposide
teniposide
sertaconazole
fluvastatin
pimozide
14
2
latanoprost
diltiazem
bromfenac
bosutinib
15
erlotinib
vincristine
amitriptyline
16
1
fluticasone
epinephrine
17
18
dabrafenib
sorafenib
diazepam
dasatinib
vemurafenib

Supplemental Figure S7. Drug combination predictions as a function of signaling network size. The number of predicted drug combinations is shown for increasing signaling network size via incremental increases in the lambda parameter that regulates protein-protein interactions (strength of evidence) and gene expression (increased fold change) for network nodes for each melanoma genomic subtype defined in the TCGA SKCM dataset: *BRAF* mutant (A), *NRAS* mutant (B), *NF1* mutant (C) and Triple wild type (D) melanoma.

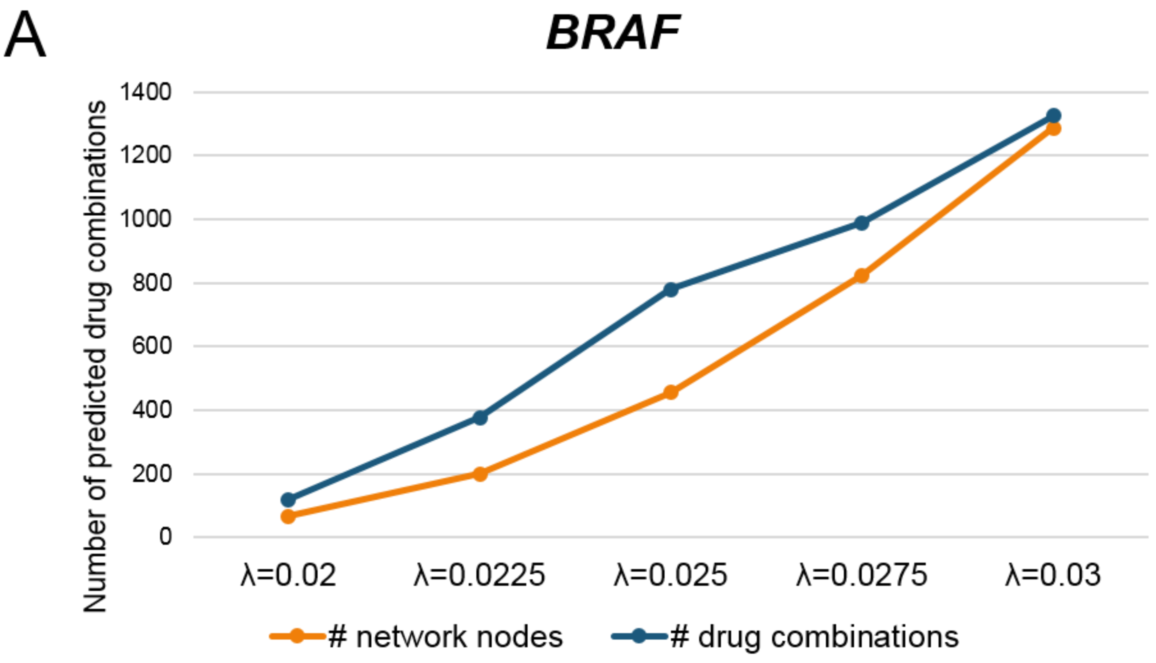

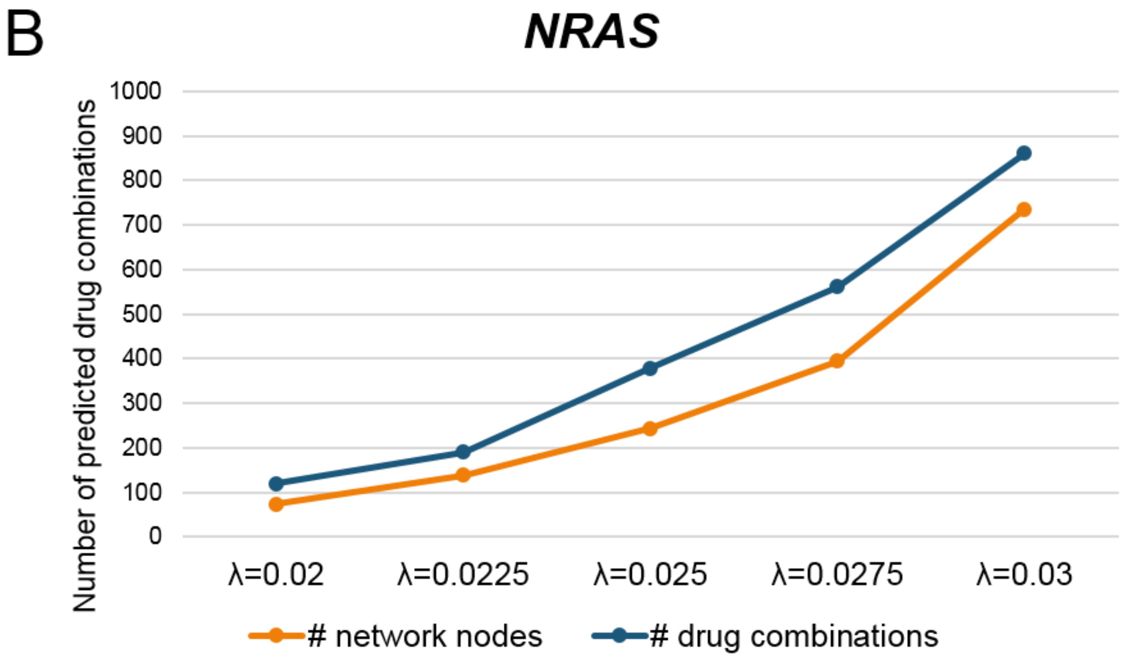

C

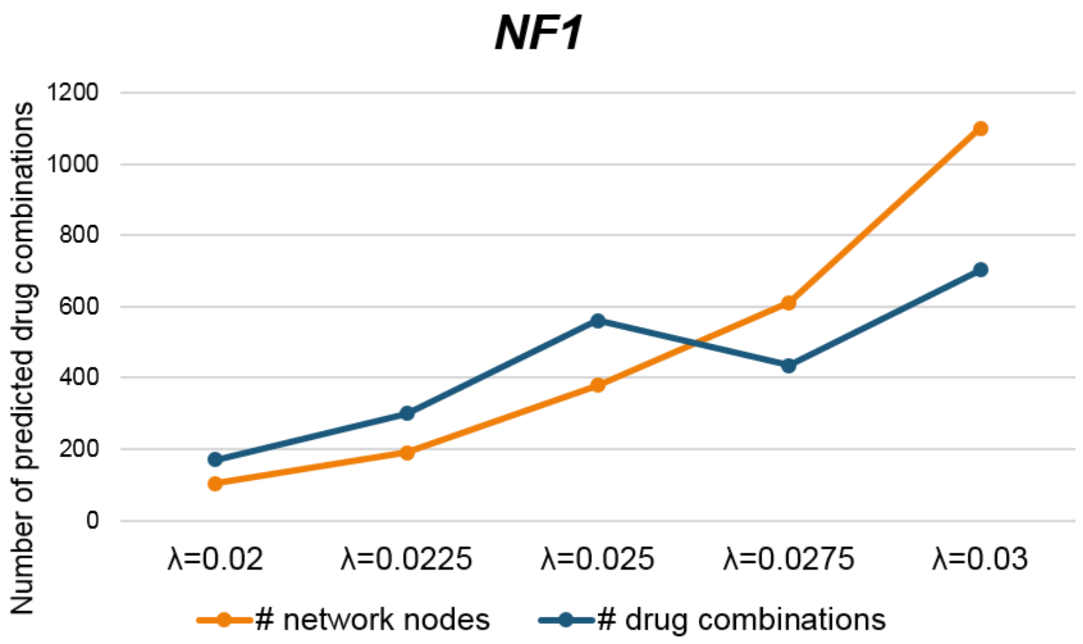
NF1
Number of predicted drug combinations
1200
1000
800
600
400
200
0
λ=0.02
λ=0.0225
λ=0.025
λ=0.0275
λ=0.03
network nodes
drug combinations

D

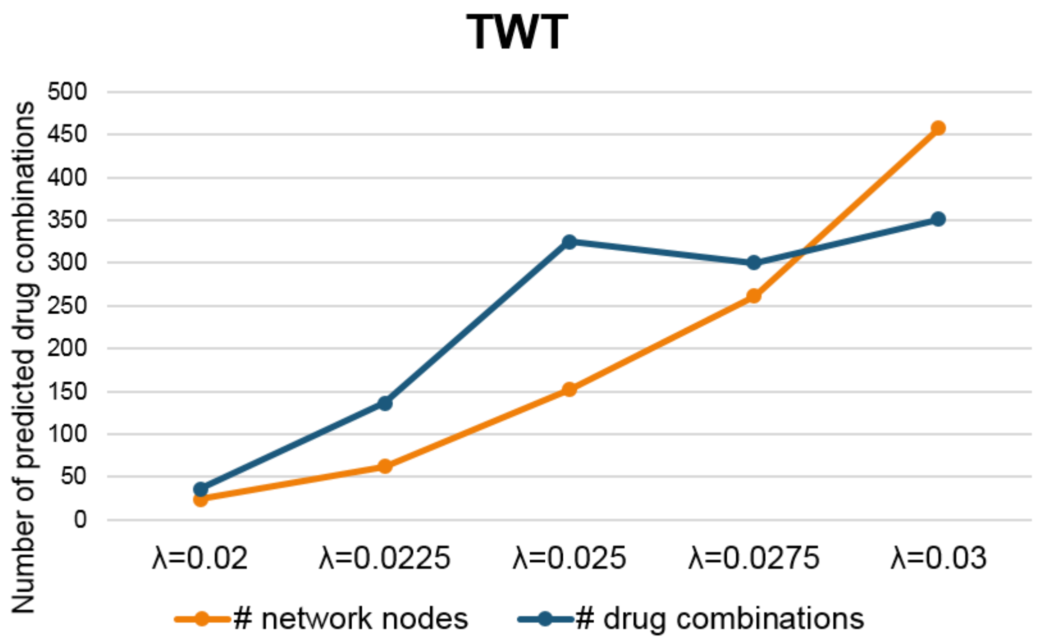
TWT
Number of predicted drug combinations
500
450
400
350
300
250
200
150
100
50
0
λ=0.02
λ=0.0225
λ=0.025
λ=0.0275
λ=0.03
network nodes
drug combinations

Supplemental Figure S8. Comparison of random drug pairs and combinations from model components. A) Using the combinatorial drug screening validation dataset results for each melanoma genomic subtype, the positive predictive values are shown for the original drug combination predictions, two general melanoma models (SMG= significantly mutated genes, DGN = DiSGeNEt genes) and random drug pairs from a set of overlapping drug combinations between the validation dataset and the set of all FDA approved therapies modeled in our method. B) The number of validated drug combinations is reported for each melanoma genomic subtype predicted by our method (original) and using only differentially expressed genes (DE) or root genes (Root) to model the signaling networks by our method.

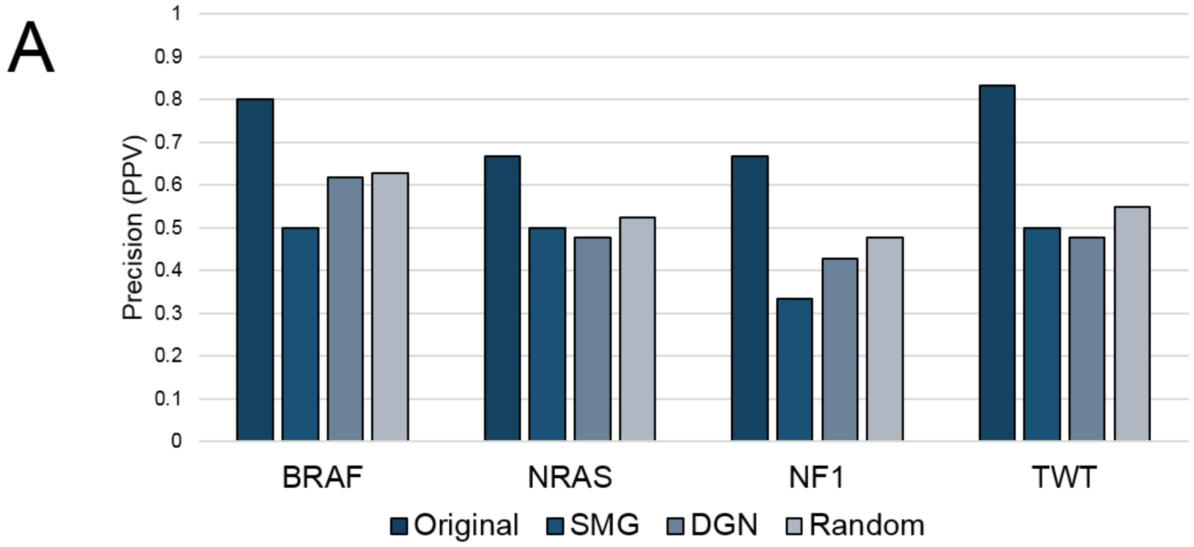

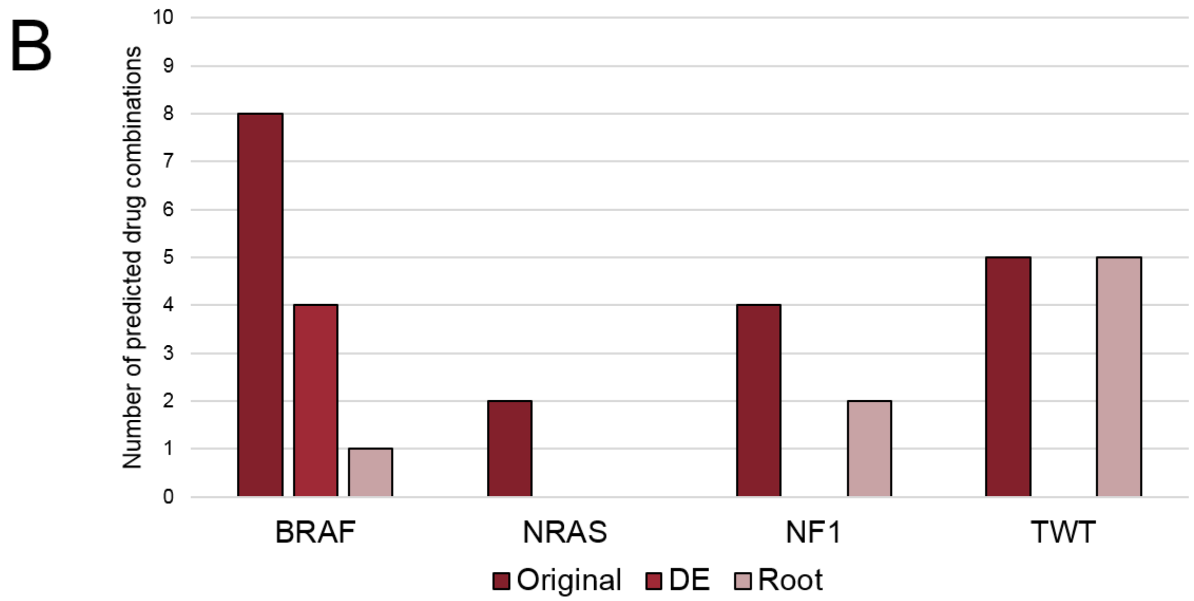

Supplemental Figure S9. Random re-wiring of signaling networks. The number of true positive and false positive drug combination predictions from the set of validated drug combinations in the BRAF melanoma signaling network is shown for the original signaling network and for 100 random permutations of protein-protein interaction pairs fixed to the size of the original signaling networks.

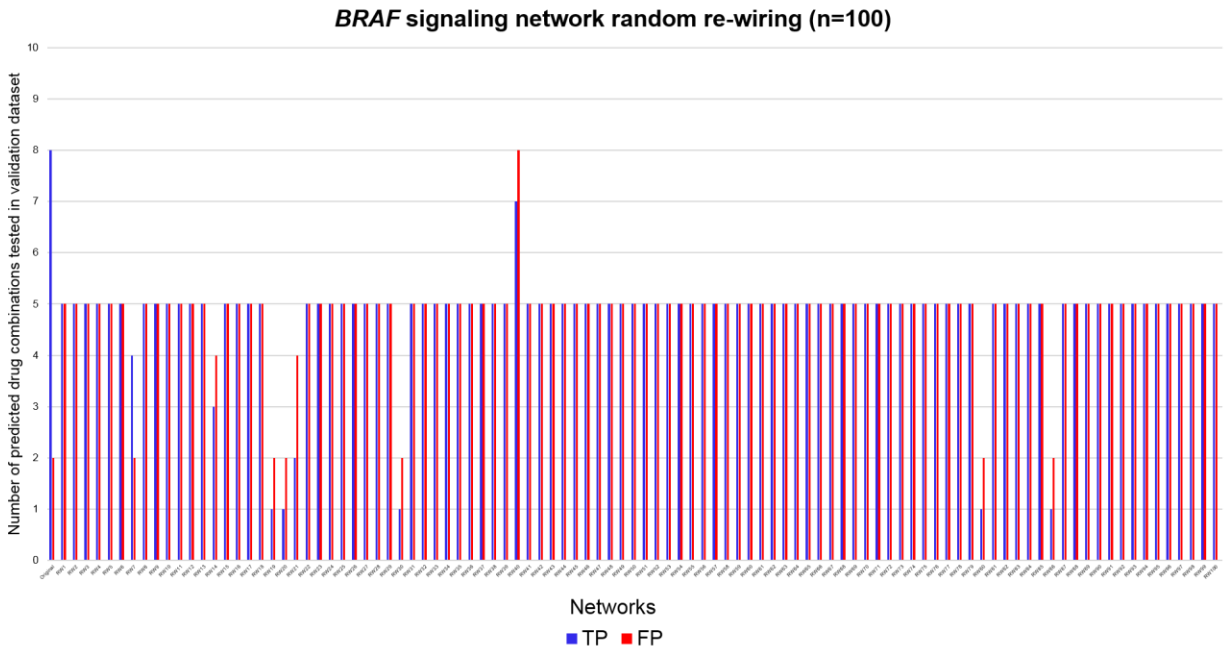

Supplemental Figure S10. Sample-to-sample distances based on A375 melanoma cell line RNA-seq gene expression data across different drug treatment groups. Sample-to-sample distances characterized by global gene expression patterns were determined by three complementary dimensionality reduction techniques using rlog-transformed RNAseq counts, including: A) hierarchical clustering with Euclidean distance, B) multi-dimensional scaling (MDS) and C) principal components analysis (PCA). DMSO = dimethyl sulfoxide vehicle control; Vem = vemurafenib; Tre = Tretinoin; VemTre = Vemurafenib+Tretinoin combination.

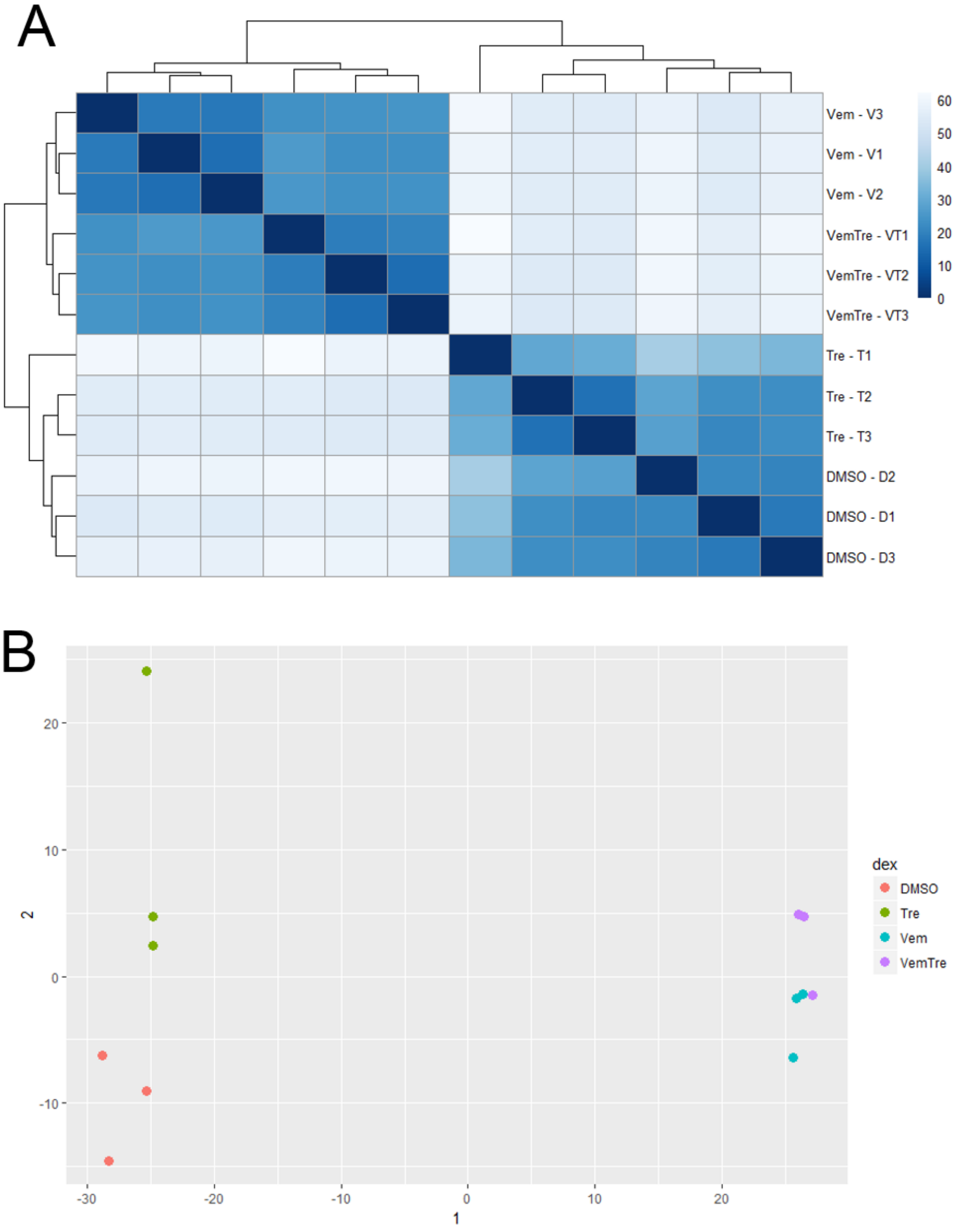

C

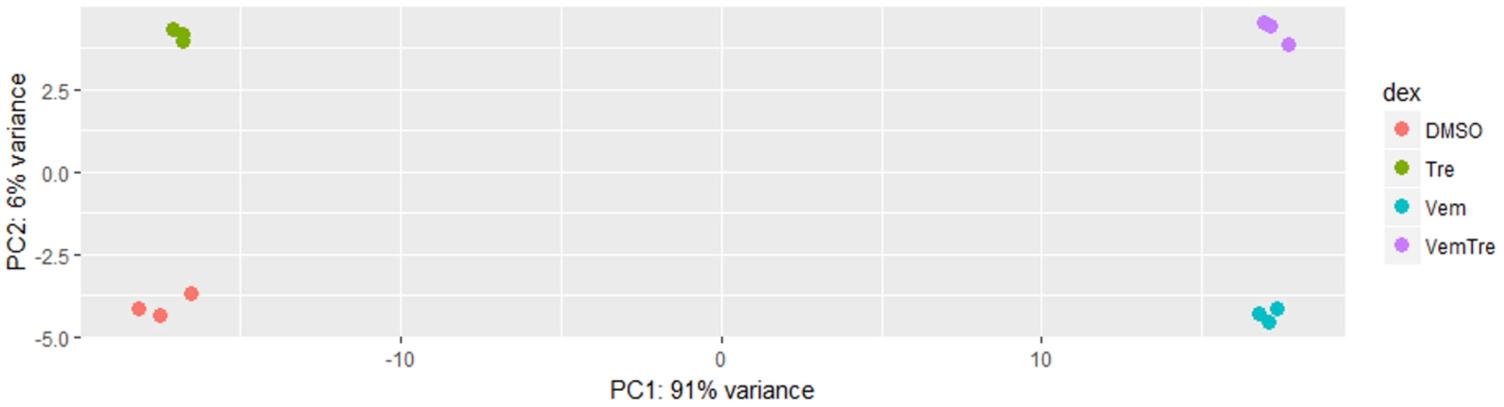

PC2: 6% variance
2.5
0.0
-2.5
-5.0
-10
0
10
PC1: 91% variance
dex
DMSO
Tre
Vem
VemTre

Supplemental Figure S11. High mRNA expression of AKT1 in melanoma tumors is associated with poor patient survival in majority of studies. A) Overall survival (left) and disease-free survival (right) shown for TCGA-SKCM melanoma patients with up-regulated AKT1 gene expression (RSEM normalized RNAseq z-score > 2.0) using data from the shown via the red line and patients without high expression in the blue line. Survival analysis for the TCGA SKCM dataset was conducted via the cBioPortal webtool. GEO gene expression datasets from melanoma tumors with corresponding survival data for melanoma patients from datasets GSE19234 (B), GSE22153 (C) and GSE53118 (D). Melanoma tumors with high (red) and low (green) expression of AKT1 were bifurcated at the median level of expression. Survival analysis for GEO datasets was conducted via the PROGgeneV2 webtool.

A

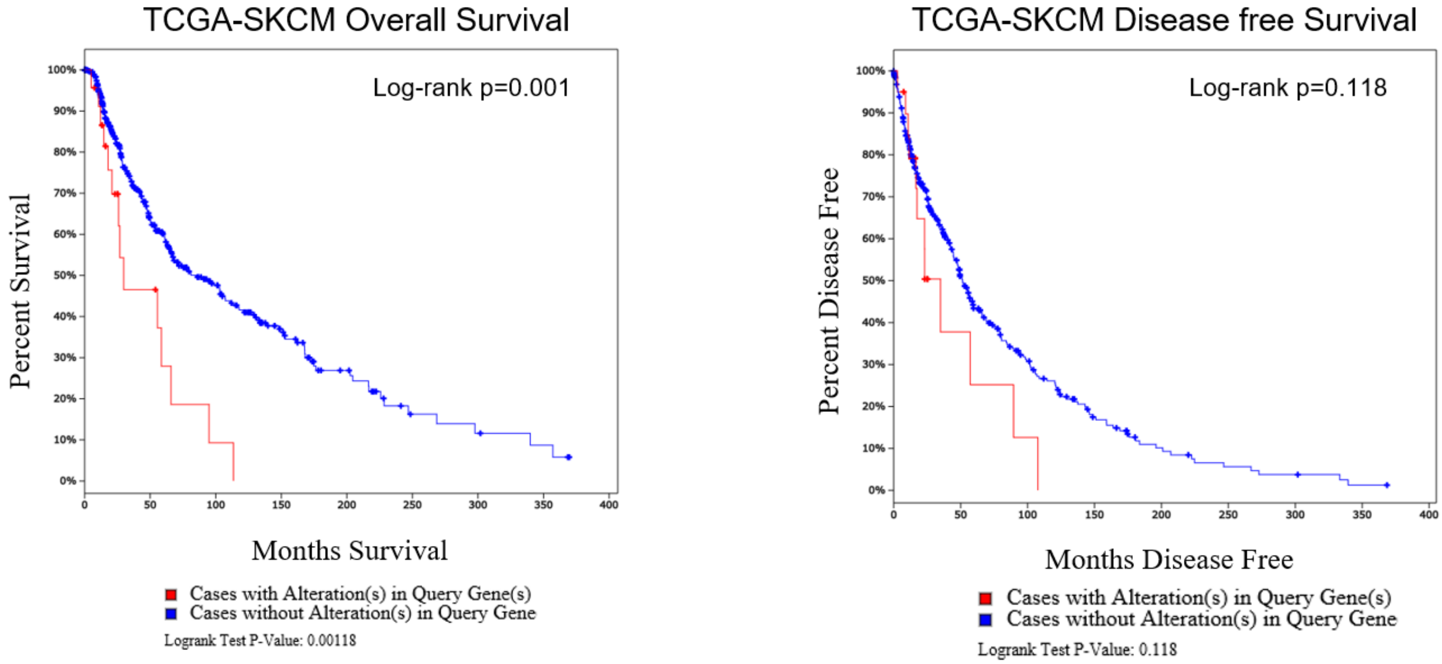

B

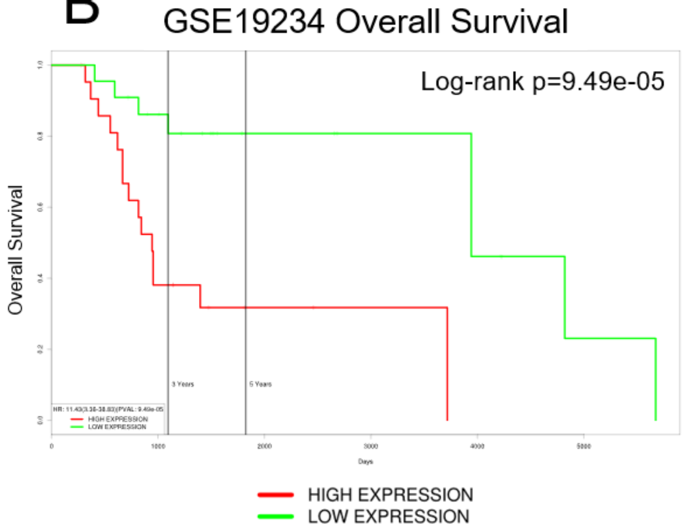
GSE19234 Overall Survival
Log-rank p=9.49e-05
Overall Survival
3 Years
5 Years
Days
HIGH EXPRESSION
LOW EXPRESSION

C

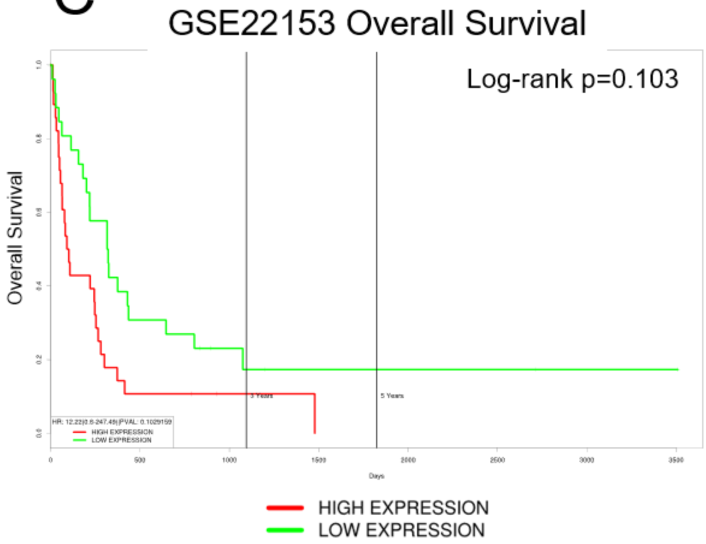
GSE22153 Overall Survival
Log-rank p=0.103
Overall Survival
3 Years
5 Years
Days
HIGH EXPRESSION
LOW EXPRESSION

D

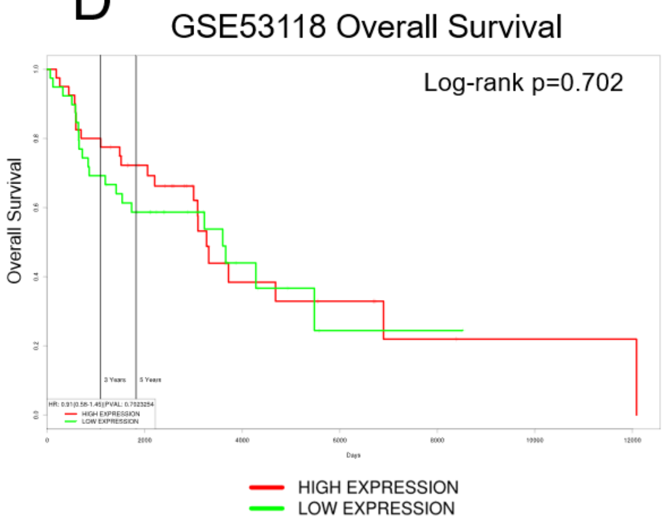
GSE53118 Overall Survival
Log-rank p=0.702
Overall Survival
3 Years
5 Years
HR: 0.91(0.58-1.45)|PVAL: 0.7023254
HIGH EXPRESSION
LOW EXPRESSION
Days
HIGH EXPRESSION
LOW EXPRESSION

Supplemental Figure S12. Distribution of RNA-seq gene count and log2-fold change values in drug-treated A375 melanoma cells. Mean-difference ("MA": minus-average) plots of are shown for each comparison between treatment of A375 cells with tretinoin (A), vemurafenib (B), vemurafenib+tretinoin combination (C) relative to DMSO vehicle control. The log2 fold change for a particular comparison is plotted on the y-axis ("M" for "minus", where subtraction of log values is equivalent to the log of the ratio) and the average ("A" for "average") of the counts normalized by size factor is shown on the x-axis. Individual genes are represented as dots color coded by statistical significance of differential expression (default threshold = adjusted P<0.1): red (significant), grey (not significant). D) Total number of mapped reads summarized as gene counts for RNA-seq data across each sample (D=DMSO, T=tretinoin, V=vemurafenib, VT=vemurafenib+tretinoin combination). Histogram plots of log2 fold changes of differential gene expression for each treatment group comparison relative to DMSO vehicle control: tretinoin (E), vemurafenib (F) and vemurafenib+tretinoin combination (G).

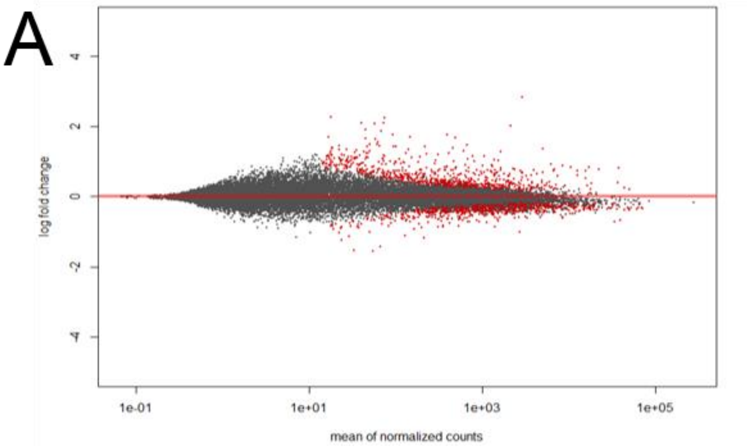

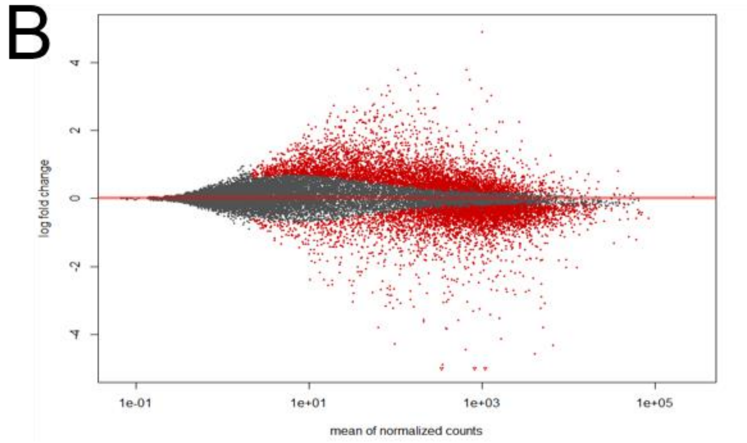

C

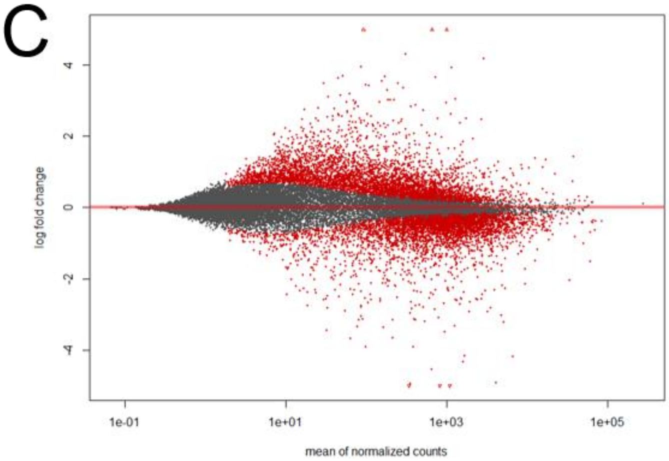

log fold change
mean of normalized counts
1e-01
1e+01
1e+03
1e+05
4
2
0
-2
-4

D

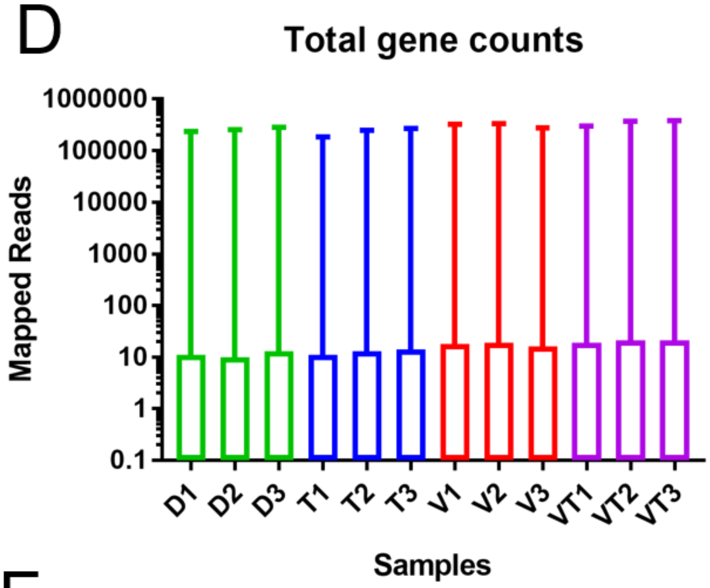

Total gene counts
Mapped Reads
Samples
1000000
100000
10000
1000
100
10
1
0.1
D1
D2
D3
T1
T2
T3
V1
V2
V3
VT1
VT2
VT3

E

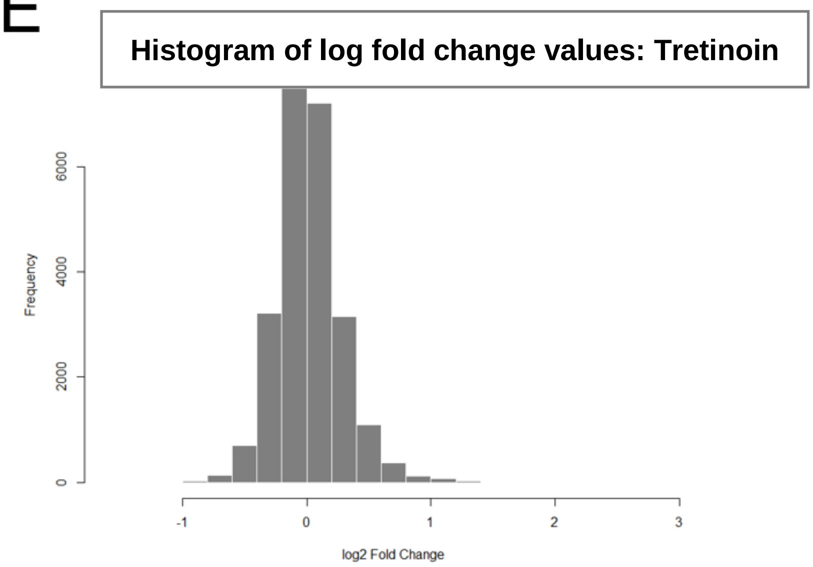

Histogram of log fold change values: Tretinoin
Frequency
log2 Fold Change
0
2000
4000
6000
-1
0
1
2
3

F

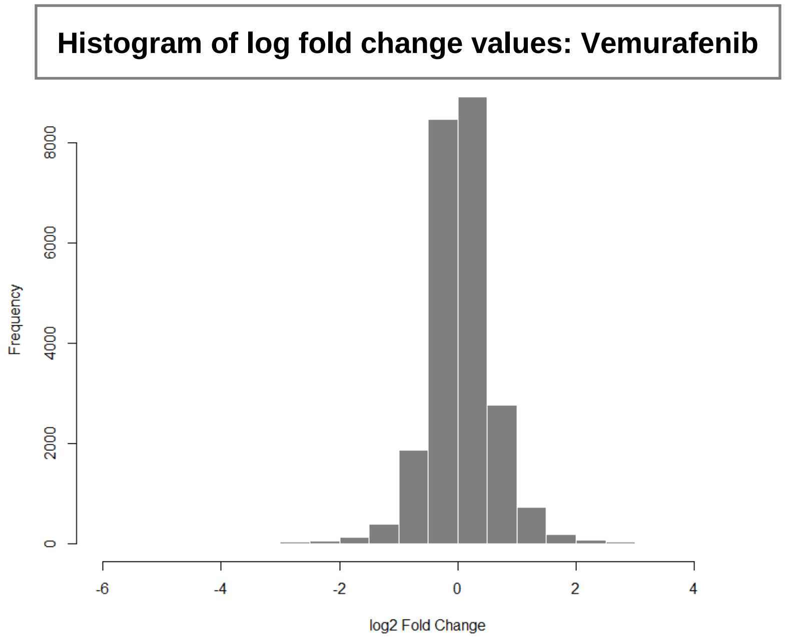

Histogram of log fold change values: Vemurafenib
Frequency
0
2000
4000
6000
8000
-6
-4
-2
0
2
4
log2 Fold Change

G

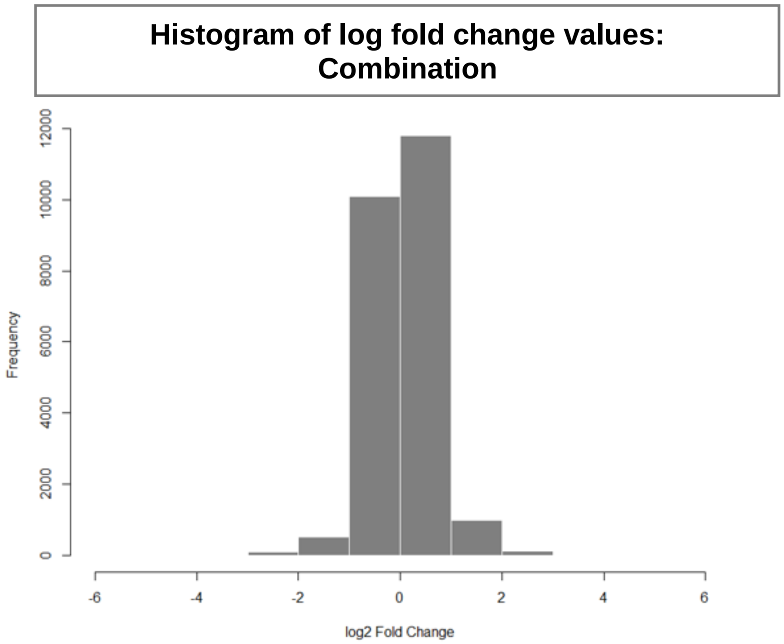

Histogram of log fold change values: Combination
Frequency
0
2000
4000
6000
8000
10000
12000
-6
-4
-2
0
2
4
6
log2 Fold Change

Supplemental Figure S13. Normalized RNA-seq gene expression count plots shown for drug target genes of tretinoin and vemurafenib for A375 melanoma cells in response to each drug treatment condition. Drug targets of tretinoin, including retinoic acid receptors (RAR) and retinoic X receptors (RXR), are shown in A-F. The drug target of vemurafenib (BRAF) is shown in G. The differential expression status of the combination treatment group relative to other treatment groups are color-coded as follows: green= DMSO vehicle control, tretinoin = blue, vemurafenib = red; where * = adj.P<0.05, ** = adj.P<0.005 and *** = adj.P <0.0005.

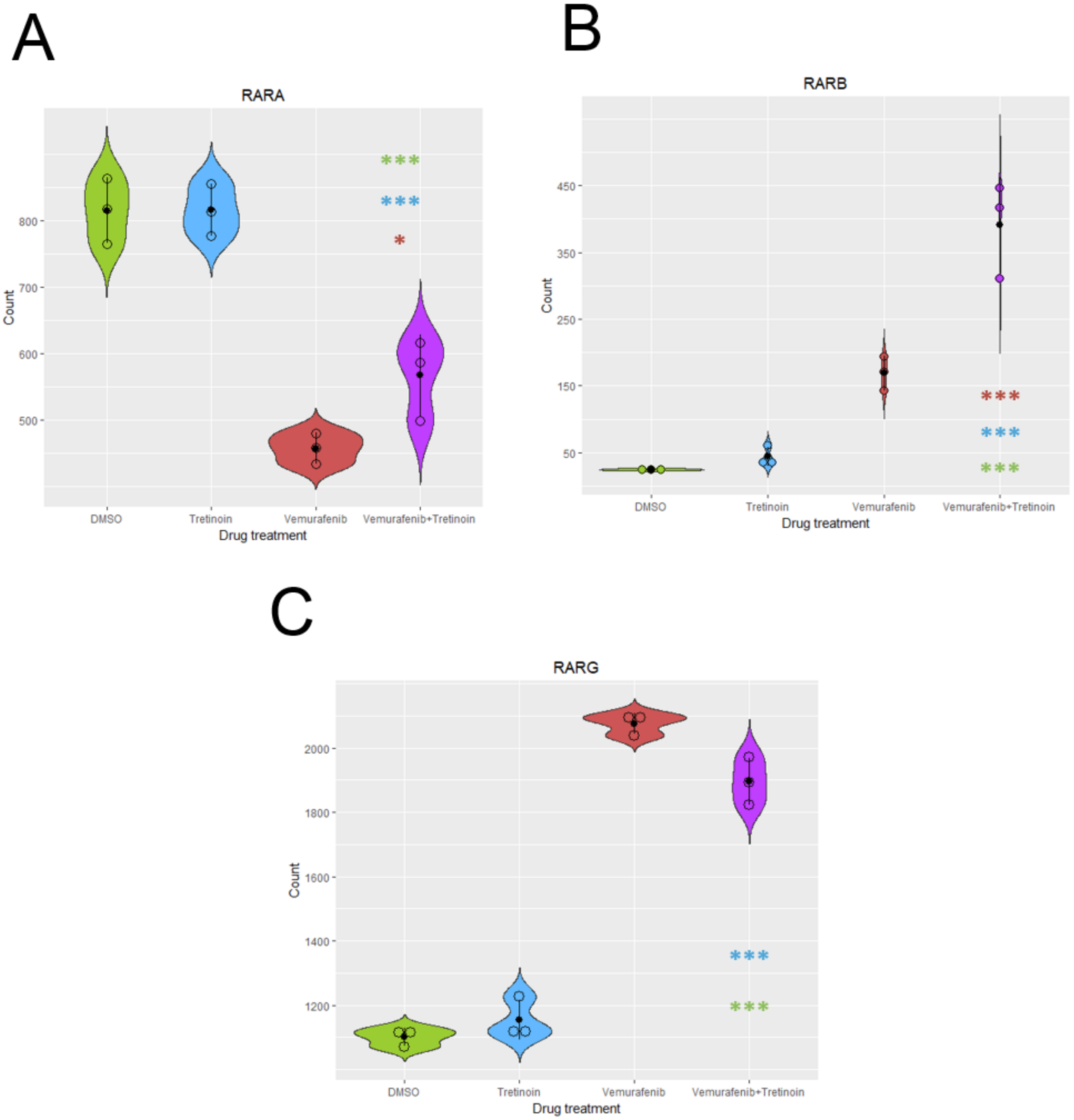

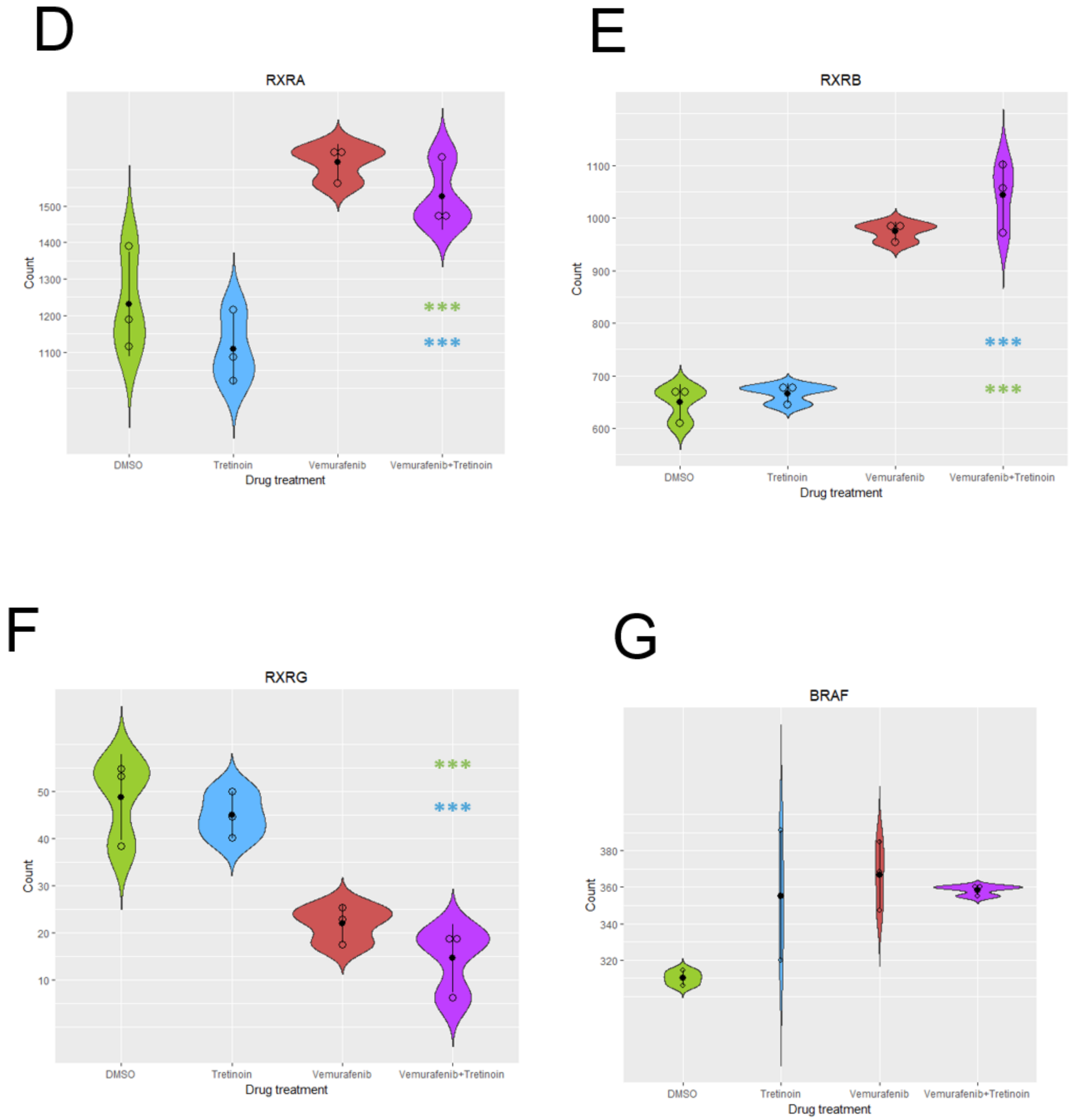
D
RXRA
Count
1500
1400
1300
1200
1100
***
***
DMSO
Tretinoin
Vemurafenib
Vemurafenib+Tretinoin
Drug treatment
E
RXRB
Count
1100
1000
900
800
700
600
***
***
DMSO
Tretinoin
Vemurafenib
Vemurafenib+Tretinoin
Drug treatment
F
RXRG
Count
50
40
30
20
10
***
***
DMSO
Tretinoin
Vemurafenib
Vemurafenib+Tretinoin
Drug treatment
G
BRAF
Count
380
360
340
320
DMSO
Tretinoin
Vemurafenib
Vemurafenib+Tretinoin
Drug treatment

Supplemental Figure S14. Flow cytometry gating strategy and representative scatterplots for double positive Annexin V/PI staining of A375 melanoma cells. A) Gating strategy for untreated/unstained A375 melanoma cells applied to all experimental conditions. The average abundance of relevant cell populations within post-sort fractions is 11,136 cells (n=48 total samples). Annexin V/PI double stained A375 cells 72 hr after drug treatment with 5 μM of: B) DMSO, C) Tretinoin, D) Vemurafenib and E) Tretinoin+Vemurafenib.

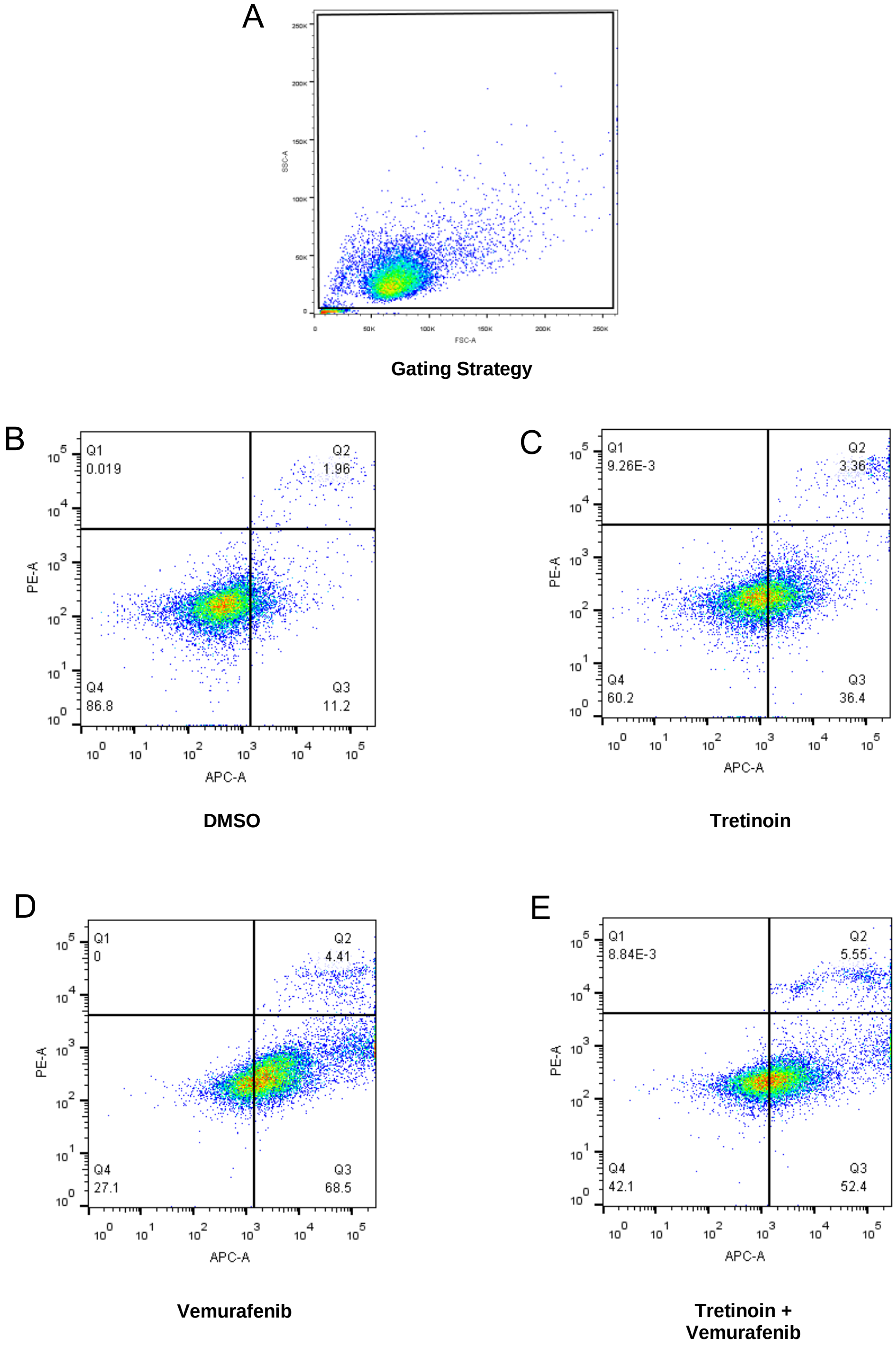

Supplemental Tables

Supplemental Table S1. Signaling networks. For each melanoma genomic subgroup-specific gene expression dataset (GSE15605 or TCGA-SKCM), interacting pairs of protein are shown for signaling networks generated using co-mutated genes observed in TCGA SKCM primary melanoma tumors associated with each major genomic subtype (BRAF, NRAS, NF1, triple wild-type).

BRAF_PrimaryMelanoma_GSE15605		NRAS_PrimaryMelanoma_GSE15605	
From	To	From	To
BRAF	YWHAZ	CTNNB1	MDM2
NEDD4L	SNCA	CTNNB1	LEF1
NEDD4L	NTRK1	NEDD4L	SNCA
CITED1	CTNNB1	CITED1	CTNNB1
APBA2	LRP2	LRP2	LRPAP1
BCL2A1	HRK	BAX	BCL2A1
CDC42	CSPG4	BIRC7	OSTM1
NTRK1	TRIM28	OSTM1	RGS20
BMPR1B	PEG10	CDC7	CDK2
AP1S2	ELAVL1	CDC7	VAC14
ELAVL1	OSTM1	CITED1	LNX1
OSTM1	RGS20	CDK2	VIM
CDC42	ELAVL1	SPRY2	UBASH3B
NTRK1	VAC14	SPRY2	TRIM51
BUB1	CHL1	BAMBI	TGFBR1
ELAVL1	SPRY2	S100A1	S100B
SPRY2	TRIM51	KHDRBS3	LNX1
BAMBI	SOX30	ASB9	VIM
BAMBI	BMPR1B	HK2	MAGEA12
S100A1	S100B	LOXL4	PRMT6
MDM2	TRIM27	AP2B1	FN1
ELAVL1	PHLDA1	ITGB1	SPP1
SPP1	TMEM30A	BCL2A1	HRK
ASB9	UBE2D1	ATM	EIF4EBP1
MAD1L1	NTRK1	CDK2	VAT1
NTRK1	PLOD3	IGF2BP3	MDM2
HK2	MAGEA12	GIT2	PXN
ELAVL1	HRK	ATM	MDM2
PNMA2	YWHAZ	MDM2	NRAS
IGF2BP3	NTRK1	ABL2	NEDD4L
E2F7	SMURF1	CDH2	CTNNB1
GAPDHS	NUSAP1	FN1	ITGB1
ASB9	TCEB1	FLOT1	FN1
MAGEA6	TRIM28	FN1	IGF2BP3
MDM2	YWHAZ	CXCL8	POMGNT1
BRAF	NEDD4L	ABL2	MDM2
MLPH	MYO5A	PAX3	POU3F2
MYO5A	NTRK1	PAX3	SOX10
MDM2	SMURF1	HK2	ITGA4
HIPK2	MDM2	FN1	PXN
CXCL1	HIPK2	ITCH	UBE2E1
NTRK1	STXBP1	DPYSL4	NUFIP1
NEDD4L	UBE2D1	TTR	VIM
PAX3	TRIM28	MPPED2	RXRG
PAX3	SOX10	FCGR2A	ITGA4
HK2	NTRK1	ITGB3	PXN

MPPED2	RXRG	FN1	PLAT
DFNA5	NTRK1	ITGA2	MMP1
DPYSL4	NTRK1	CERS2	FLOT1
SDF4	TTR	ITGA4	VIM
IRF4	PEG10	FN1	MIA
APBA2	STXBP1	PKN1	VIM
NTRK1	PKN1	PKN1	PLAUR
BMPR1B	SMURF1	CBX3	MDM2
ELAVL1	PLAUR	CBX3	SP100
MBP	PKN1	ADAM10	PXN
FN1	IGF2BP3	LAMA1	PLAT
ELAVL1	KRAS	MBP	PKN1
DLGAP1	MYO5A	MBP	PLP1
FN1	PLAT	L1CAM	NUFIP1
MMP3	NUCB1	CDK2	CDK5R1
BCAN	MMP1	LNX1	NRCAM
BCAN	MMP3	ITGA10	ITGB1
FN1	MIA	UPP1	VIM
SRA1	UBE2T	SQSTM1	TRIM63
TRIM63	UBE2D1	UBASH3B	UBE2E1
MMP10	MMP3	CDC7	CHL1
BCAN	MMP13	MDM2	TCEB1
LAMA1	PLAT	ITCH	MLANA
MBP	PLP1	HMGA2	PRMT6
BACE2	TMEM30A	LEF1	PRMT6
CDC42	CPN1	NUFIP1	SNCA
NRCAM	NTRK1	CDK5R1	NES
UBE2D1	UPP1	BIRC7	UBE2E1
ELAVL1	NEDD4L	MDM2	TUBB4A
NEDD4L	TBC1D7	NEDD4L	WNK4
MYEF2	NTRK1	MDM2	TRIM9
MAGEC2	SSX1	PEG10	TGFBR1
MDM2	TUBB4A	ITGA4	TUBB2B
ARNT2	TRIM27	POU3F2	SOX10
LGALS3BP	NTRK1	NEDD4L	TMEM55A
SDF4	SNCA	PRAME	TCEB1
NTRK1	TUBB2B	ACOT7	CDK2
POU3F2	SOX10	NRAS	VAT1
KIF14	NTRK1	INSC	LNX1
MAGEA2B	TRIM28	LNX1	SLC6A15
MAGEC2	TRIM28	CTNNB1	HERC5
ELAVL1	NUSAP1	ITGB1	LGALS3BP
ELAVL1	ZNF280B	GNG2	NUFIP1
ELAVL1	SERPINE2	SPRY2	SPRY4
ELAVL1	SNX10	MPPED2	VAC14
ELAVL1	TTYH3	EIF2B2	IGF2BP3
ELAVL1	TMEM30A	CERS2	ELOVL2
ELAVL1	NRIP3	CA14	EIF2B2
ELAVL1	GAS2L3	LNX1	POMGNT1
ELAVL1	SLC16A4	GIT2	QPRT
PRAME	TCEB1	MCOLN3	POMGNT1
NTRK1	RHOBTB3	PRMT6	TRIB2
SMURF1	TRIB2	SERPINE2	SP100
SPRY2	SPRY4	AP1S2	AP2B1
ALX1	SRA1	ATM	MAGEC2
MPPED2	VAC14	ACOT7	MAGEA6
C1QB	FN1	MAGEC2	SSX1

CA14	KIF14	LINC00518	VAC14
MDM2	NUCB1	ADAM10	TSPAN10
MOK	SDF4	FRMD5	NRCAM
KIF14	PNKD	COLGALT2	TMEM255A
LINC00518	VAC14	CHSY3	PLAUR
CTNNA2	NTRK1	HOXD13	PLAUR
FRMD5	NRCAM	EIF2B2	PNKD
C1QB	COLGALT2	TMEM255A	UBASH3B
FOXF2	SDF4	CTNNA2	CTNNB1
CHSY3	PLAUR	CDK2	PRKAR1A
HOXD13	PLAUR	ARNT2	TRAF1
CHST11	SDF4	MEOX2	PRKAB2
GAPDHS	SLC45A2	RGS20	TBC1D16
KIAA1211	NTRK1	SETDB1	VIM
KRAS	STK32A	PRKAB2	SPRY2
ABCB5	KRAS	C2orf88	PRKAR1A
KRAS	LOC285556	AP2B1	ITGA4
CTNNB1	PRKCA	MEOX2	SPP1
ATF2	CALU	CDK2	IGF2BP3
BYSL	TRIM27	IGF2BP3	MEOX2
NTRK1	PRKCA	GIT2	TRAF1
TRIM27	UBE2D1	MDM2	SETDB1
EIF4EBP1	PRKCA	ITCH	MDM2
BYSL	PNMA2	MLPH	RAB27A
MDM2	TCEB1	LRIF1	RAB27A
MDM2	UBE2D1	MLPH	MYO5A
GLI3	PRKCA	FN1	VIM
CTNNB1	SMURF1	MEOX2	PAX3
HMGA2	PRKCA	LRIF1	ZNF24
KRAS	UBE2D1	SETDB1	ZNF24
MBP	PRKCA	ASAH1	SETDB1
ELAVL1	FN1	SETDB1	TTR
GLI3	KRAS	PGBD1	ZNF24
BCAN	FBXO11	ITCH	TGFBR1
ATF2	SRA1	ITCH	STAM
ELAVL1	FBXO11	MEOX2	PGBD1
ELAVL1	PRKCA	CDK5R1	CTNNB1
CALU	NTRK1	STAM	TRIM63
GPRIN1	SDF4	ZNF24	ZSCAN9
PAX3	POU3F2	PRMT6	ZSCAN9
ATF2	GLUD2	MYEF2	TRAF1
GPRIN1	NTRK1	ARNT2	MEOX2
NTRK1	TTN	SRPK2	ZSCAN9
CTNNB1	NTRK1	CA14	NUP160
BYSL	SMURF1	MCOLN3	SRPK2
BUB1	CDC42	KCNN2	SRPK2
NTRK1	VIM	NUP160	PNKD
ASB9	VIM	TBC1D16	TRAF1
MDM2	S100B	FRMD5	PRKAR1A
NTRK1	PTPRS	ASAH1	PNKD
IGF2BP3	MDM2	ITCH	TMEM255A
SMURF1	VIM	GSK3B	SNCA
ELAVL1	HK2	NPM1	PRKAR1A
TTR	VIM	GSK3B	VIM
PKN1	PLAUR	CDK2	NPM1
MMP3	PTPRS	DNAJC13	GSK3B
TRIM63	TTN	MDM2	NPM1

UPP1	VIM	AURKA	NPM1
ELAVL1	NTRK1	FN1	NPM1
CTNNB1	ELAVL1	CDK6	SOX10
ELAVL1	IGF2BP3	CDK2	CDK6
PKM	TRIM63	AP2B1	TGFBR1
BCAN	MMP10	FN1	ITGA4
NTRK1	PKM	DNAJC13	NPM1
ELAVL1	ZBTB20	CDK5R1	CDK6
CDK2	NTRK1	HMGA2	NPM1
BYSL	RNF2	DNAJC13	OGN
ADNP	NTRK1	ARPC1B	AURKA
PEG10	RNF2	ARPC1B	SRPK2
BYSL	TRIP6	ICAM1	NPM1
CDC6	CHL1	CNPY3	COLGALT2
CDC6	CDK2	COLEC11	COLGALT2
GAPDHS	TRIP6	CNPY3	OGN
PNMA1	PNMA6A	BAX	MDM2
ADNP	RNF2	FN1	SNRPD1
ARNT2	TRIP6	CTSB	SPRY2
ADNP	ZSCAN20	IFIT3	SNRPD1
GLUD2	RNF2	PARP1	SPP1
ADNP	QPRT	CCT6A	CDK2
		CCT6A	MDM2
		MDM2	PARP1
		CTSB	PARP1
		CCT6A	FN1
		IRF4	PEG10
		HOXB7	PARP1
		CCT6A	LGALS3BP
		CCT6A	TUBB2B
		SNRPD1	SRPK2
		ICAM1	SNRPD1
		BAX	SNCA
		CTNND2	LNX1
		LNX1	TRIM23
		EIF4EBP1	PRKCA
		MDM2	TRIM23
		ITGB1	PRKCA
		LNX1	PRKCA
		TRIM23	UBE2E1
		CTNND2	TTR
		AKAP12	PRKCA
		ITGA2	ITGB1
		MBP	SQSTM1
		ASB9	TCEB1
		HMGA2	PRKCA
		ARNT2	TRIM23
		MPPED2	TRIP13
		CTNNB1	SMAD2
		LEF1	SMAD2
		APBA2	LRP2
		PIN1	TRAF1
		HEXIM2	SPRY2
		PIN1	SMAD2
		CDK2	SMAD2
		CDK2	LYN
		ITCH	SMAD2

DYRK3	NEDD4L
NUFIP1	STXBP1
NEDD4L	SMAD2
APBA2	STXBP1
SMAD2	TGFBR1
ITGA4	PIN1
PKN1	TRAF1
LYN	PLAUR
COL11A2	PIN1
HEXIM2	PIN1
LNX1	TRIP13
LNX1	SAT1
HOXB6	SAT1
SAT1	SETDB1
HOXB6	PLSCR1
PLSCR1	SPRY2
PHLDA1	PLSCR1
LOXL4	TRIP13
CDK4	SETDB1
EIF4EBP1	LRPAP1
DPYSL4	TRIP13
EVA1A	SAT1
PLEKHA4	SETDB1
CDK4	CDK6
CBX3	FN1
BIRC7	HTRA2
ARNT2	PLSCR1
PIN1	SNCA
C19orf66	TRAF1
LNX1	MTUS2
C19orf66	SPRY2
MTUS2	SLC23A1
PNMA2	SDCBP
CTNNB1	PARP1
ITGA4	SDCBP
MTUS2	SDCBP
CDK5R1	MTUS2
SNCA	SQSTM1
ARNT2	MTUS2
C19orf66	MTUS2
MITF	TFEC
LEF1	MITF
PDE4D	TRAF1
PRKACB	SPP1
CDK2	MITF
CDK2	TSC22D1
ATM	EIF3E
RGS1	TSC22D1
CCT6A	ITGA4
CDK2	EIF3E
EIF3E	TRIM63
CCT6A	UBASH3B
CDK2	PDE4D
PRKACB	PRKAR1A
EIF3E	ICAM1
MAGEA6	PDE4D
CDK2	FASN

		FASN	MDM2
		FASN	FN1
		ITGA4	SNRPD1
		IGF2BP3	SYK
		ITGB3	SYK
		ITGA4	SYK
		PRKD1	SYK
		SYK	UBASH3B

NF1_PrimaryMelanoma_GSE15605		TWT_PrimaryMelanoma_GSE15605	
**From**	**To**	**From**	**To**
BAX	BCL2A1	BAX	SNCA
ANKRD28	PPP1R9B	BAX	MDM2
CDKN2A	PPP1R9B	BAX	BCL2A1
S100A1	S100B	S100A1	S100B
CDKN2A	MDM2	MDM2	TRIM27
MDM2	UBE2D1	MDM2	UBE2D1
TRIM63	UBE2D1	TRIM63	UBE2D1
MDM2	TCEB1	MDM2	TCEB1
MDM2	MS4A1	MDM2	MS4A1
ACACB	ANKRD28	IL12RB1	TUBB4A
MDM2	TUBB4A	IL12RB1	IL12RB2
PRAME	TCEB1	MDM2	TUBB4A
BAX	MDM2	PRAME	TCEB1
MDM2	S100A1	MDM2	S100B
RNF114	UBE2D1	MDM2	PPT1
IL12RB1	TUBB4A	CCL5	CCR1
IL12RB1	IL12RB2	CCL5	CXCL8
BRCA2	PCNA	CCR1	GNAQ
MDM2	PCNA	GNAQ	PPT1
CDK2	CDK5R1		
CDK2	LMNA		
LMNA	MDM2		
IFIT3	ISG15		
ISG15	LMNA		
BUB1	MAD2L1		
BUB1	SMC2		
MDM2	PLK1		
MAD2L1	UBD		
CXCL9	CXCR3		
CXCL10	CXCR3		
CXCL9	SYNE4		
CXCL11	CXCR3		
CXCL13	CXCR3		
GZMB	LMNA		
SMC2	SYNE4		
ISG15	PLOD3		
PIN1	SNCA		
BCL2A1	NR4A1		
ISG15	TOP2A		
NR4A1	TOP2A		
NEDD4L	UBE2D1		
CNKSR1	PIN1		
PIN1	TOP2A		
NEDD4L	SNCA		
TCEB1	TOP2A		
NR4A1	TUBB4A		
CTNND2	LNX1		

MDM2	UBE2D2
LNX1	UBE2D2
TRIM63	UBE2D2
LNX1	SLC6A15
CDK2	LYN
MDM2	S100B
DENND2C	LYN
LYN	TUBB4A
LNP1	LYN
KLC1	SNCA
KLC1	USP7
MDM2	USP7
KLC1	SYNE4
TRIM63	USP7
ENTPD3	SYNE4
HERC5	ISG15
DDX39A	MDM2
DDX39A	FAT3
HELZ2	ISG15
CDK2	EIF3E
ANP32A	ATXN1
ANP32A	VPS72
CLP1	VPS72
IFIT1	IFIT3
SPP1	VPS72
ANP32A	EIF3E
ATXN1	PSPH
ATXN1	NR4A1
CLP1	HMCN1
EIF3E	TRIM63
ASB9	TCEB1
MBP	PLP1
ATXN1	KIAA1549L
ASB9	H2AFZ
H2AFZ	VPS72
ATXN1	BATF2
AP2B1	FN1
FN1	IGF2R
FN1	MIA
IGF2R	PLAUR
EXT1	SYNE4
C1QB	FN1
AP2B1	NUSAP1
GAPDHS	NUSAP1
EXT1	PLAUR
GAPDHS	SLC45A2
BAX	SNCA
PPL	USP7
KAZN	PPL
ARRB2	SRPK2
ARRB2	MDM2
SRPK2	UBD
MCOLN3	SRPK2
KCNQ5	MCOLN3
KCNN2	SRPK2
GRB2	VIM
AP2B1	GRB2

ISG15	VIM
GRB2	KRT83
ATXN1	MBP
GRB2	KIAA1549L
ASB9	VIM
LRP2	MAGI1
EGFR	MEGF6
EGFR	SNCA
ISG15	STAT1
EGFR	SMC2
EGFR	SOCS3
EGFR	STAT1
SOCS3	TCEB1
IL12RB2	SOCS3
EGFR	TUBB4A
MYBPC1	TRIM63
CDK2	USP7
LMNA	USP7
MYO3A	USP7
ISG15	PALLD
GMEB1	TRIM63
ISG15	MX1
ACACA	MDM2
ACACA	KIF5B
ACACA	PRKAA2
KIF5B	SYNE4
LCK	PTPRC
CD8A	PTPRC
LCK	MS4A1
GRB2	ROS1
MDM2	SRC
SOCS1	TCEB1
MDM2	RRBP1
SAMD9	SHMT2
MDM2	SHMT2
LYN	SLITRK4
LMNA	SYNE1
DTNBP1	KIF5B
DTNBP1	SYNE1
SSX2IP	TRIM37
TRIM37	UBE2D1
SSX2IP	SYT17
HLA-A	UGGT1
MDM2	TRAF5
CD8A	HLA-A
TNFRSF14	TRAF5
HLA-A	ITM2B
ITM2B	SYNE4
SNRPD1	USP7
IFIT3	SNRPD1
ICAM1	YBX2
ICAM1	SNRPD1

**BRAF_PrimaryMelanoma_TCGASKCM**		**NRAS_PrimaryMelanoma_TCGASKCM**	
**From**	**To**	**From**	**To**
ARF3	ELAVL1	CAPN1	VIM

CTNNB1	MSN	CTNNB1	MDM2
PGK1	YWHAZ	SNCA	SQSTM1
CDK2	MYC	NPM1	PRKAR1A
FN1	SMAD4	FN1	SMAD4
FTL	SDCBP	ITGA3	ITGB1
SDCBP	TSPAN3	KRT15	KRT6B
BRAF	YWHAQ	CCT6A	VIM
ABCE1	LMNA	MDM2	PTK2
ELAVL1	MITF	S100A1	S100B
PDIA3	PLXNA1	JUP	MDM2
AKT1	CTNNB1	NONO	SFN
CTTN	STAT1	KRT10	SFN
NPM1	PRKAR1A	CDK2	GRN
SHC1	STXBP1	PARP1	TRIM29
CDH1	JUP	FTH1	SDCBP
NONO	SFN	FTL	SDCBP
AHCY	PXN	MDM2	TPM3
BCAR1	YWHAZ	PHC2	SDCBP
HNRNPA2B1	SERPINH1	S100A2	S100B
CAPNS1	CSPG4	ITGB1	SPP1
STMN1	TRIM28	CD63	SDC1
HNRNPA2B1	HNRNPAB	CAP1	PXN
MCL1	MYC	SHC1	STXBP1
CCT5	CCT7	ERBB3	SHC1
ITCH	SFN	TNFRSF14	VIM
GGA2	IGF2R	CDK2	PUF60
CDK2	SORT1	CDK2	VAT1
ABCE1	VIM	EIF4A2	TPM3
COPS6	SAT1	CDK2	TSC22D1
BRAF	SFN	CDK2	CTSA
CUL1	KRT6B	MMP14	TIMP2
LASP1	MAP4	CDK2	SORT1
S100A1	S100B	FN1	GSN
AP2B1	GRB2	MDM2	NRAS
ELAVL1	PHLDA1	ANKS1A	SFN
JUP	MSN	LAD1	SFN
PTBP1	SLC3A2	ITGB1	LGALS3BP
ILF3	IVNS1ABP	FLOT1	FN1
PARP1	SPP1	CTNNB1	PARP1
SQSTM1	STXBP1	ATP1A1	TPM3
MED15	UBAP2L	PGK1	SFN
GRN	RAC1	LGALS3BP	TUBA1A
HSPD1	PLOD3	CTSB	PARP1
KRT6A	SHC1	CSTB	CTSB
KRT17	PA2G4	LRPAP1	SORT1
GRB2	SIRPA	DSP	PKP1
ANXA5	TERF2IP	DSP	KRT1
FTH1	SDCBP	CDK6	SOX10
ELAVL1	ITPKB	MORF4L1	TUBA1A
STMN1	TPM3	MDM2	RAB7A
PHC2	SDCBP	ANXA5	NDRG1
CANX	RPN2	PRKCSH	TUBA1A
ECM1	MCL1	ASAH1	TSC22D1
ABCE1	TPP1	ICAM1	RPL35A
PGK1	TPI1	PTK2	STAT1
CCT6A	CCT7	KPNB1	MDM2
MYC	YWHAQ	CDK2	CDK6

IVNS1ABP	PARP1	FBXO7	PSMB4
GRB2	SHC1	FN1	PLAT
ERBB3	PA2G4	DAG1	TUBA1A
ABCE1	CBX3	GRN	SLPI
BRAF	HSP90AB1	FASN	NPM1
BCAR1	MMP14	PKN1	VIM
TNFRSF14	VIM	CD63	SDCBP
PUF60	SRRM2	KRT1	SFN
FDFT1	FN1	IVL	KRT1
COPS6	CUL1	FN1	IGF2R
ARHGDIA	PDIA3	SP100	TRIM29
MAP3K11	RAC1	KRT1	KRT5
TSC22D1	VIM	CCT6A	FASN
DYNC1H1	YBX1	PUF60	SDCBP
SGK1	YWHAQ	MBP	PKN1
ICAM1	SNRPB	MBP	PLP1
CALML5	MAGED1	AHNAK	MDM2
GANAB	TNS3	SDC3	TUBA1A
FN1	GSN	KRT14	KRT5
CDK2	CTSA	NPM1	SQSTM1
MMP14	TIMP2	ITGB1	TGOLN2
AHNAK	VARS	KRT17	MDM2
MAGED1	MAGED2	EIF3E	NPM1
FN1	MYH10	CDK6	FBXO7
CALM3	GRB2	FN1	SRM
GNAS	GNB1	H2AFZ	MORF4L1
NPM1	PA2G4	CALU	NPM1
DDOST	HSP90AB1	CALU	WARS
NCL	PA2G4	GALNT2	TGOLN2
CANX	PDIA6	SLC39A6	SPP1
KPNB1	NCL	LDHB	PGK1
LDHB	MIF	CDK2	COPA
CTNNA1	JUP	APOD	CDK2
HMG20B	VIM	NAP1L1	NPM1
CLIC4	EZR	LDHB	WDR1
MSN	WDR1	CDK2	MARCKSL1
ELAVL1	TFAP2A	NRAS	VAT1
MYC	PRKDC	ARPC1B	CALU
PRKDC	XRCC5	ATP6V1B2	JUP
ANKS1A	SFN	PSAP	SNCA
HNRNPU	MAGED2	MDM2	RRBP1
KRT1	SFN	CAP1	MYL12A
CD81	NAMPT	IFI16	MDM2
FN1	RCC2	ERBB3	TNS3
AKT1	YBX1	CD59	SMAD4
ATP1A1	PHB	GPNMB	SMAD4
MLPH	RAB27A	EIF3E	ICAM1
MLPH	MYO5A	CRTAP	SP100
PGK1	YWHAQ	CAPN1	GNS
KRT10	SFN	SERPINE2	SP100
DDOST	PHB	KRT15	PKN1
ELAVL1	TXNIP	SETDB1	VIM
HSP90AB1	TUBB	MDM2	SFN
MAP4	SERBP1	GRN	NPM1
CTSB	PARP1	PARP1	SPP1
ABCE1	CSTB	SDC1	SDCBP
CST3	CSTB	CAP1	FASN

CANX	DDOST	CLIC4	SHC1
ABCE1	IGF2R	ERBB3	MDM2
LRPAP1	SORT1	PUF60	VIM
KPNB1	SREBF2	CDK2	VIM
ATP2A2	S100A1	FECH	TIMP2
PEA15	TERF2IP	MDM2	SETDB1
CDK2	S100A6	FN1	ITGB1
DSP	KRT1	MDM2	PARP1
LDHB	PPIA	FN1	VIM
PAX3	TRIM28	PAX3	SOX10
PAX3	SOX10	KPNB1	MORF4L1
CHD4	ELAVL1	CLIC4	NDRG1
ANXA5	TUBB	SETDB1	ZNF24
GPAA1	PIGT	ASAH1	SETDB1
FN1	RAB7A	PGBD1	ZNF24
AKT1	CD81	CLIC4	PSMB4
CSDE1	JUP	CLIC4	DAG1
COPS6	SFN	MDM2	NPM1
EZR	L1CAM	PKN1	PLAUR
COPS6	PSAP	CBX3	SP100
MYC	MYO9B	MBP	SQSTM1
SERBP1	SFN	RAD23B	VIM
LAMC1	PDIA3	CDK2	CLIC4
APOD	SUMF2	PLAUR	PSAP
MYC	USP22	ICAM1	IFI16
FKBP9	HEXA	FECH	MDM2
ASAH1	TSC22D1	PLAUR	PLTP
ATP6AP1	HNRNPAB	CAPN1	FN1
ECH1	GSTO1	CDK2	CTNNB1
MYL6	STMN1	CTNNB1	SMAD4
COPS6	PAEP	FASN	TPM3
ABCE1	RPL35A	NDRG1	S100B
FN1	GSTO1	FASN	SHC1
GAPDH	TPI1	FN1	PUF60
CAPNS1	YWHAZ	CDK2	NPM1
ECH1	TIMP1	NPM1	PARP1
HNRNPU	SLC7A5	CTNNB1	TUBA1A
CD63	TSPAN3	FN1	NPM1
CDK2	COPA	FN1	RAB7A
MBP	SQSTM1	FN1	KPNB1
FN1	HSP90AB1	DNAJC13	NPM1
ABCE1	CD44	FN1	KRT5
KRT1	KRT5	AHNAK	FN1
CLIC4	PSMB4	KRT17	SHC1
COX4I1	GNB1	DNAJC13	OGN
CLIC4	DAG1	ITGB1	PTK2
ANXA5	PDIA3	CCT6A	SHC1
FN1	PLAT	CCT6A	CDK2
IGFBP5	SPP1	CCT6A	MDM2
PTTG1IP	STX7	FASN	FN1
FN1	MIA	CDK2	KPNB1
HNRNPU	SND1	CD63	ITGB1
HSP90AB1	HSPD1	KRT5	SHC1
ICAM1	TUBB	CCT6A	LGALS3BP
FN1	TNC	SQSTM1	STXBP1
ATP1A1	EIF3B	TTR	VIM
HMGA1	NPM1	AHNAK	S100B

GLG1	ITM2B	FN1	PARP1
ARF4	LDHB	PAX3	RAD23B
TLN1	YWHAQ	CTSA	PLAUR
AGRN	PDIA3	CBX3	FN1
CAPNS1	PDIA4	PUF60	RAD23B
COL6A1	PDIA3	CTNNB1	SMAD2
ACTR2	FN1	PRKAR1A	SMAD2
CCT6A	FASN	KRT6B	UCHL5
SERPINH1	STX7	CTNNB1	NONO
MBP	PLP1	NEDD4L	SFN
ELAVL1	ETV5	FTL	UCHL5
CD81	TUBA1B	FN1	TPM3
BACE2	GGA2	CAP1	FN1
FBXO7	PSMB4	DYRK3	NEDD4L
SDC3	TUBB	CDSN	UCHL5
HNRNPU	KRT14	KRT10	KRT5
LYST	YWHAZ	CCT6A	FN1
CALM3	MYO10	PKP1	UCHL5
SF3B2	TMED9	NEDD4L	SMAD2
ELAVL1	HNRNPA2B1	CTNNB1	NDRG1
GNAS	MAGED2	KPNB1	SMAD2
CAPRIN1	CDK2	PSMB4	UCHL5
FN1	UBAP2L	PKN1	TUBA1A
AHCY	LDHB	IVL	UCHL5
NUCB1	WARS	CBX3	MDM2
CDK2	GLG1	CCT6A	SMAD2
EZR	H2AFZ	RAD23B	UCHL5
ITCH	MLANA	SMAD2	UCHL5
YWHAQ	ZC3H13	PSAP	SMAD2
PDIA3	PLOD1	ICAM1	NPM1
LAMP2	TUBB	CDK4	SNCA
LDHB	PPT1	CDK4	SETDB1
FLG	MCL1	PLEKHA4	SETDB1
LGALS3BP	MAGED2	CDK4	CDK6
CALU	MSN	CDK2	PRKAR1A
ILF3	RRBP1	KRT31	KRT6B
ACADVL	CDH1	CAP1	TPM3
AEBP1	GRB2	AARS	CDK2
ABCE1	HM13	KRT31	SLC23A1
LYPD3	USP22	KRT5	MDM2
NAP1L1	NCL	PLAUR	TUBA1A
ACTR2	ARPC1A	FN1	KRT31
CDK2	MARCKSL1	CDK2	DSP
ELAVL1	KDELR2	AARS	STAT1
ELAVL1	SERPINE2	KRT31	PKN1
ELAVL1	TTYH3	KRT31	KRT5
CIRBP	FN1	CCT6A	LDHB
ELAVL1	MLEC	ANXA5	CDK2
ALDH1A3	ELAVL1	SQSTM1	TUBA1A
HNRNPU	LAPTM4A	S100A1	S100A2
FKBP9	WARS	AARS	WARS
ILF3	LAMP1	CDK2	NAP1L1
DMKN	ELAVL1	AARS	FN1
CANX	PMP22	ATP6V1B2	FN1
ELAVL1	SEPN1	MITF	TFEC
ELAVL1	IFI6	CDK2	JUP
MAGED2	UBB	CDK2	MITF

PRAME	SNRPB	CDK2	PARP1
TERF2IP	TMSB10	CDK2	FASN
LDHB	TPI1	FASN	MDM2
RPS24	SERBP1	SHC1	TUBA1A
CDK2	VAT1	FN1	PXN
CUL1	QPCT	KPNB1	SMAD4
CUL1	KRT6C	PTK2	PXN
CD99	PPIA	ERBB3	SYK
ACTR2	ARPC1B	CDK2	PGK1
ATP6V1B2	JUP	EIF4A2	SYK
ABCE1	FLOT1	PGK1	SYK
HSPD1	SCARB2	SYK	TUBA1A
GANAB	PRKCSH	NPM1	SYK
SERPINH1	TMED9	EIF3E	FN1
MYL12A	MYL6		
CNDP2	PDIA3		
IFITM3	NAMPT		
ABCE1	CD59		
GPNMB	SMAD4		
ABCE1	GNB1		
ICAM1	IFI16		
GAPDH	GAS7		
ACTR2	CRTAP		
ABCE1	GNS		
COX4I1	TPM3		
PPT1	VGF		
ABCE1	MFGE8		
LAPTM4B	LYPD3		
CLCN7	LYPD3		
CANX	PLTP		
ATP1A1	SSR2		
CTNNA1	CTNNB1		
FBXW11	YWHAZ		
CDK2	SRRM2		
PUF60	SDCBP		
FN1	YWHAQ		
AKT1	SFN		
CDK2	PRKAR1A		
CLIC4	SHC1		
CDH1	LMNA		
HNRNPU	NONO		
SERPINH1	VIM		
FN1	TRIM28		
AHNAK	HNRNPAB		
MCL1	USP9X		
ITCH	UBAP2L		
ELAVL1	SORT1		
FN1	ILF3		
MED15	ZC3H13		
TERF2IP	XRCC5		
CSNK1A1	DYNLL2		
S100A6	S100B		
FN1	PARP1		
FN1	GRB2		
C1QBP	MMP14		
CDK2	CUL1		
FBXW11	GLI3		

DYNC1H1	RPS24
ELAVL1	SGK1
FN1	SNRPB
HNRNPU	SRRM2
GANAB	JUP
MAGED1	TRIM28
CTNNA1	FBXW11
EZR	FN1
FN1	PRKDC
FBXW11	HNRNPU
PA2G4	YBX1
DYNLL2	MYO5A
CST3	CTSB
CANX	PTBP1
ATP2A2	SLC3A2
HNRNPA2B1	PGK1
CD81	NONO
COPS6	KRT5
RCAN1	USP22
FN1	MYL6
GAPDH	PGK1
CAPNS1	FN1
ELAVL1	SLC7A5
CD63	SDCBP
CSNK1A1	GLI3
MBP	PRKDC
FN1	KPNB1
FN1	HNRNPU
PDIA3	VIM
FN1	SND1
C1QBP	FN1
HMGA1	PA2G4
FN1	TLN1
ACTR2	CCT5
ELAVL1	STX7
FN1	TUBA1B
COPS6	FBXO7
KRT14	KRT5
KPNB1	LYST
SF3B2	YBX1
ELAVL1	FN1
MAGED2	NAP1L1
FBXW11	UBAP2L
FASN	TPM3
ELAVL1	LAPTM4A
FN1	LDHB
ABCE1	MSN
NUCB1	PRKDC
NTRK1	YWHAZ
CDK2	RAC1
ELAVL1	SDCBP
AKT1	PRKDC
CTTN	GRB2
ELAVL1	PRKAR1A
KIAA1211	NTRK1
ERBB3	SHC1
CDH1	KRT1

FN1	NONO
FN1	PXN
ELAVL1	SERPINH1
CCT5	CCT6A
FN1	VIM
AHNAK	LASP1
AP2B1	FN1
NTRK1	PTBP1
ILF3	RPS24
ILF3	KRT17
PARP1	TERF2IP
FTH1	GRB2
AHNAK	S100B
FN1	MYC
ERBB3	GRB2
HSP90AB1	NTRK1
NTRK1	PUF60
CUL1	KRT5
FN1	RAC1
DYNC1H1	NTRK1
CSDE1	GANAB
ABCE1	CTSA
MAGED1	NTRK1
FN1	PDIA6
FN1	SLC3A2
FN1	MIF
CTNNA1	CTTN
CSDE1	XRCC5
IVNS1ABP	YBX1
MYO5A	NTRK1
KRT10	KRT5
COX4I1	PHB
AHNAK	ARHGDIA
NTRK1	TUBB
CANX	CCT6A
ATP2A2	SPP1
FN1	PPIA
NTRK1	PIGT
CSDE1	CUL1
CANX	L1CAM
ELAVL1	SUMF2
HEXA	USP22
EZR	HEXA
FN1	TUBB
CUL1	PSMB4
DAG1	ELAVL1
HSPD1	PDIA3
FN1	HSPD1
FN1	HMGA1
ARF4	ELAVL1
NTRK1	TLN1
ACTR2	ELAVL1
TUBA1B	TUBB
CTTN	SDC3
SF3B2	SRRM2
CAPRIN1	CIRBP
AHCY	MSN

GLG1	NTRK1
FASN	NTRK1
NES	NTRK1
INTS1	TGOLN2
INTS1	NTRK1
HNRNPU	LAMP2
ELAVL1	MARCKSL1
ELAVL1	FKBP9
AP3D1	NTRK1
FN1	RPS24
CD99	FTH1
ATP6V1B2	CSDE1
CANX	PRKCSH
ERBB3	TNS3
NTRK1	PCDHGC3
GAS7	HNRNPU
CNP	NTRK1
NTRK1	VGF
IGSF3	NTRK1
BCAR1	GRB2
ELAVL1	ITCH
ELAVL1	GRB2
CTNNB1	USP9X
SGK1	YWHAZ
CALM3	ELAVL1
ELAVL1	EZR
CTSA	NAMPT
CANX	ELAVL1
COPS6	TUBB
ELAVL1	LAMC1
SND1	USP22
LAMC1	NID1
COL4A1	NID1
COL4A1	COL4A2
ELAVL1	ZBTB20
UBAP2L	YBX1
AKT1	SMAD4
CALML5	YWHAQ
GRB2	ITCH
ADNP	MYC
PARP1	TUBB
CDK2	TSC22D1
CALML5	ZSCAN20
FBLN1	YWHAQ
EZR	WDR1
ZNF24	ZSCAN20
ATP1A1	CCT6A
MYO5A	RAB27A
COPS6	ZNF24
CBX3	FN1
FBLN1	NID1
C1QBP	MYC
ACTR2	STXBP1
ADNP	ZSCAN20
SBSN	ZSCAN20

NF1_PrimaryMelanoma_TCGASKCM		TWT_PrimaryMelanoma_TCGASKCM	
**From**	**To**	**From**	**To**
CDK2	LMNA	CDC42	STMN1
LMNA	NUMA1	CDK2	MIF
LMNA	SDCBP	LMNA	NUMA1
CTTN	LMNA	LMNA	MDM2
CTSD	MAPK1	CDK2	MITF
CAP1	TPM3	CTTN	SDC3
GRB2	VIM	MAGED2	USP7
AHNAK	LASP1	CAP1	FASN
S100A1	S100B	GRB2	VIM
AP2B1	GRB2	AHNAK	LASP1
ISG15	PGK1	S100A1	S100B
SELENBP1	TMED9	AP2B1	FN1
SAT1	VKORC1	SLC15A3	TRIM27
FTH1	SDCBP	SAT1	VKORC1
FTL	SDCBP	FN1	RPS15A
STMN1	TPM3	FTH1	SDCBP
ITGB1	SPP1	FTL	VKORC1
GRB2	PHC2	TPM3	TRIM27
CBX3	MDM2	MIF	YKT6
TNFRSF14	VIM	ITGB1	SPP1
ARHGDIA	PDIA3	GRB2	TRIM27
CANX	DDOST	CBX3	MDM2
CDK2	CTSA	TNFRSF14	VIM
CDK2	SORT1	ARHGDIA	PDIA3
GNAS	HLA-A	CANX	DDOST
GNAS	GNB1	CDK2	CTSA
MSN	PGK1	CDK2	SORT1
FN1	GSN	GNAS	HLA-A
CCND1	CDK2	EIF3E	TRIM27
MDM2	PLK1	GNAS	GNB1
FLOT1	FN1	NDRG1	SGK1
ANKRD28	SDCBP	MSN	PGK1
PGK1	TPI1	GAB2	GRB2
MAGED2	TUBA1A	FN1	GSN
STXBP1	TUBB2A	CCND1	CDK2
MDM2	TUBB2A	MDM2	TRIM27
MDM2	PARP1	FLOT1	FN1
CDK2	NOV	CD63	SDCBP
FN1	LMNA	PGK1	TPI1
LRPAP1	SORT1	TUBA1A	TUBB2A
L1CAM	PEA15	STXBP1	TUBB2A
LDHB	MIF	MDM2	TUBB2A
CD63	ITGB1	PARP1	SPP1
TFAP2A	YBX1	FN1	GRB2
ANXA5	PDIA3	LRPAP1	SORT1
CANX	MDM2	L1CAM	PEA15
MDM2	RAB7A	CD63	ITGB1
CTSD	PSAP	MORF4L1	TUBA1A
CANX	L1CAM	TFAP2A	YBX1
S100A13	VAT1	ANXA5	PDIA3
CAPNS1	FN1	CANX	ITGB1
CNDP2	GBP2	MDM2	RAB7A
ECH1	MDM2	CTSD	PSAP
ECH1	TIMP1	CANX	L1CAM
ISG15	STAT1	CAPNS1	FN1

STAT1	STAT2	CANX	NDRG1
CD44	ITGB1	ECH1	MDM2
CDC42	MAP3K11	ECH1	TIMP1
MDM2	PDIA3	CTTN	STAT1
GYPC	PDIA3	CD44	ITGB1
FN1	PLAT	CDC42	MAP3K11
IGFBP5	SPP1	ITGB1	PDIA3
PTTG1	PTTG1IP	GYPC	PDIA3
FN1	MIA	FN1	PLAT
ICAM1	IFI16	DSP	MDM2
HLA-A	TAP1	IGFBP5	SPP1
HLA-A	TAP2	PTTG1IP	STX7
CD63	SDCBP	FN1	MIA
HLA-A	ITM2B	PKN1	VIM
CBX3	SP100	HLA-A	TAP1
FECH	TIMP2	CD63	VKORC1
MMP14	TIMP2	MDM2	TCEB1
FN1	YBX1	HLA-A	ITM2B
CTSD	MDM2	CBX3	SP100
MAPK1	MBP	FECH	TIMP2
PDIA3	TAPBP	MMP14	TIMP2
AHNAK	ARHGDIA	FLOT1	YBX1
SDC3	TUBB2A	CTSD	MDM2
CALM3	MYO10	MBP	PKN1
CALM3	GRB2	MBP	PLP1
ITGB1	TGOLN2	STX7	YKT6
MICAL1	VIM	PDIA3	TAPBP
CDK2	GRN	AHNAK	ARHGDIA
TPP1	USP7	SDC3	TUBB2A
CTSB	PARP1	CALM3	MYO10
FN1	SRM	CALM3	GRB2
ACACB	ANKRD28	ARHGDIA	COTL1
CAPRIN1	CDK2	CDK2	GRN
PHC2	SDCBP	FN1	ST13
FASN	MDM2	TPP1	USP7
ITGB1	LGALS3BP	CTSB	PARP1
CALU	MSN	PHC2	SDCBP
MDM2	RRBP1	FASN	LMNA
CUTA	GRB2	H2AFZ	MORF4L1
CNDP2	PDIA3	ITGB1	LGALS3BP
GBP1	ISG15	CALU	MSN
ISG15	PLOD3	MDM2	RRBP1
CTSD	WARS	CUTA	GRB2
LDHB	TPI1	GNAS	MAGED2
CDK2	COPA	CNDP2	PDIA3
CDK2	MARCKSL1	CTSD	WARS
CDK2	VAT1	PRAME	TCEB1
CIRBP	MDM2	LDHB	TPI1
ACTR2	ARPC1B	CDK2	COPA
ATP6V1B2	FN1	AHNAK	STMN1
GALNT2	TGOLN2	CDK2	MARCKSL1
AK2	MDM2	CDK2	VAT1
DPP7	SELENBP1	CIRBP	MDM2
MDM2	TMED9	ARPC1B	CALU
CAP1	MYL12A	ATP6V1B2	FN1
FTL	VKORC1	MDM2	TMED9
IFITM3	VKORC1	CAP1	MYL12A

PDIA3	PLXNA1	SLC15A3	VKORC1
LRRC59	MDM2	IFITM3	VKORC1
IFI16	MDM2	PDIA3	PLXNA1
GNAS	MAGED2	IFI16	MDM2
C1QB	FN1	ABL2	GPX1
GPX1	SELENBP1	MDM2	NUCB1
MDM2	NUCB1	CRTAP	SP100
CRTAP	SP100	CNDP2	GNS
CNDP2	GNS	SERPINE2	SP100
SERPINE2	SP100	FECH	MDM2
FECH	MDM2	TIMP2	TIMP3
TIMP2	TIMP3	CANX	PLTP
CANX	PLTP	ARHGDIA	CDC42
ITPK1	MAPK1	ARHGDIA	CDK2
CDK2	PARP1	CTTN	LMNA
LMNA	PARP1	LMNA	USP7
CAP1	FN1	CAP1	TPM3
FN1	VIM	CYTH3	GNAQ
ISG15	MSN	AP2B1	GRB2
SAT1	TUBB2A	SAT1	TUBB2A
FTH1	FTL	FTL	SDCBP
FN1	GRB2	STMN1	TPM3
FN1	MSN	AHNAK	GRB2
CEBPA	PARP1	ARHGDIA	MDM2
FN1	PARP1	EIF3E	NUMA1
FN1	ITGB1	GNAQ	GNAS
NUMA1	PTTG1	GNAS	MDM2
PKN1	VIM	MAGED2	TUBA1A
FLOT1	YBX1	LMNA	PARP1
MBP	PKN1	ABL2	MDM2
GRB2	MICAL1	LDHB	MIF
MYL12A	MYL9	CANX	PDIA3
ARPC1B	CALU	FASN	NDRG1
BRCA2	CDK2	GNAQ	GNB1
CDK2	NUMA1	MDM2	PDIA3
CTTN	STAT1	PKN1	TUBA1A
BRCA2	PCNA	FN1	YBX1
CDK2	VIM	AHNAK	MDM2
FTH1	SPP1	FASN	MDM2
MDM2	PCNA	CNDP2	LDHB
CDK2	TUBB2A		
ITGB1	PDIA3		
BRCA2	MORF4L1		
GBP2	GNS		
CDK2	PDIA3		
RCAN1	STAT2		
MDM2	TCEB1		
ICAM1	YBX1		
FBLN1	NOV		
CTSD	LDHB		
MBP	PLP1		
H2AFZ	MORF4L1		
PRAME	TCEB1		
FBLN1	IFI30		
IFI30	TIMP3		
BUB1	CDC42		
ARHGDIA	CDK2		

CTTN	GRB2
AHNAK	ISG15
FTH1	GRB2
MDM2	S100B
CDC42	GRB2
CBX3	FN1
ARHGDIA	CDC42
ABL2	GRB2
CANX	PDIA3
FN1	RAB7A
BUB1	SVIL
DAPK1	SVIL
AHNAK	CDC42
CNDP2	LDHB
FN1	LDHB
CDC42	STMN1
CIRBP	FN1
MAGED2	TPM3
ABL2	GPX1
DAPK1	IFI30
CDK2	PIN1
CCND1	MAPK1
CAPRIN1	MDM2
CNKSR1	PIN1
MAP3K11	PIN1
CAPRIN1	PIN1
ATP6V1B2	PIN1
CDK2	LYN
PARP1	SPP1
DENND2C	LYN
LYN	SRC
MDM2	SRC
LYN	TTYH3
CDK2	FLNC
LMNA	MDM2
MAPK1	YBX1
FLNB	ISG15
FLNB	MDM2
FLNC	FN1
MAPK1	PEA15
ABL2	MDM2
FLNB	FLNC
CAPN3	FLNC
FLNC	NAT8L
TAP1	TAPBP
TAP1	TAP2
FBLN1	FN1
AHNAK	FLNB
FAT3	NAT8L
LMNA	MORF4L1
LMNA	RB1
ANP32A	VPS72
CAP1	FASN
CLP1	VPS72
FN1	LASP1
MDM2	S100A1
ISG15	LMNA

MDM2	TPM3
SPP1	VPS72
HDAC5	PARP1
CLP1	HMCN1
MDM2	PGK1
MORF4L1	TUBA1A
ACTR2	STXBP1
MORF4L1	TUBB2A
MDM2	MIF
MORF4L1	VPS72
RB1	TFAP2A
CANX	ITGB1
PSAP	THRAP3
GBP2	SAT1
MDM2	TPI1
GNB1	HDAC5
PKN1	TUBA1A
PDIA3	TAP1
LMNA	THRAP3
AHNAK	MDM2
H2AFZ	VPS72
ADAR	HDAC5
ACTR2	CALU
CNDP2	PEPD
ISG15	WARS
FN1	STMN1
PEPD	SPP1
ANP32A	ARPC1B
LGALS3BP	MAGED2
ACTR2	CRTAP
GNS	PEPD
CDK2	MIF
PSMF1	QKI
MIF	YKT6
TUBA1A	TUBB2A
FN1	IGF2R
ECH1	GSTO1
FN1	GSTO1
PTTG1IP	STX7
STX7	YKT6
FBXO7	PSMF1
FASN	TPM3
FN1	QKI
CDV3	RRBP1
CDV3	STX7
LMNA	LRRC59
NUCB1	WARS
CIRBP	RTN4
ARRB2	MAPK1
LMNA	USP7
PDIA3	VIM
AHNAK	GRB2
ARRB2	SRPK2
MDM2	SLC12A2
LRPAP1	RTN4
PCNA	YBX1
CALU	PCNA

MCOLN3	SRPK2
KCNQ5	MCOLN3
HEXA	PCNA
MCOLN3	SLC12A2
CLCN7	MCOLN3
CDK2	PRC1
MAPK1	PML
USP11	USP7
PRC1	SH3KBP1
ABI2	SH3KBP1
ISG15	VIM
FTL	GRB2
GRB2	SH3KBP1
GNAS	NUCB1
MDM2	USP7
PML	USP7
ATP6V1B2	TUBA1A
DDN	SH3KBP1
NET1	USP11
MAGI1	NET1
PML	SP100
ACTR2	CTTN
ABI2	MBP
ABI2	GPX3
FN1	RPS15A
GALNT2	RPS15A
IFI30	PRC1
CDK2	TPM3
EGFR	MEGF6
CTTN	EGFR
EGFR	TPM3
ARHGDIA	MSN
GNAS	MDM2
CDK2	MSN
GSN	MDM2
EGFR	FN1
ANXA5	CDK2
CD44	MSN
HLA-A	TAPBP
SDC3	TUBA1A
EGFR	TPP1
ADAR	EGFR
CDK2	LDHB
CDK2	RPS15A
CDK2	STMN1
CNP	EGFR
ISG15	PALLD
FBXO7	PINK1
FASN	ISG15
PINK1	VIM
MAGED2	USP7
ABI2	CAP1
ABI2	PRKAA2
DDOST	FN1
BAIAP2L1	MDM2
BAIAP2L1	PRKAA2
CD44	FN1

CD44	PTPRC
ICAM1	MSN
LDHB	PGK1
CDV3	QKI
CD63	VKORC1
GRB2	ROS1
ARHGDIA	MDM2
FASN	FN1
LGALS3BP	TUBA1A
CALU	WARS
LMNA	SHMT2
CTTN	SHMT2
SHMT2	TPM3
SAMD9	SHMT2
MDM2	SHMT2
PARP1	SHMT2
MDM2	UBE2D2
SHMT2	STAT1
FBXO7	UBE2D2
COPA	SHMT2
LYN	SLITRK4
CDC42	SYNE1
LMNA	SYNE1
FASN	LMNA
CDC42	SSX2IP
HDAC5	NUMA1
CAP1	CDC42
HDAC5	PKN1
CHCHD3	MDM2
ITGB1	TUBA1A
PKN1	SSX2IP
AHNAK	STMN1
SSX2IP	SYT17
PHC2	SSX2IP
CHCHD3	SSX2IP
TNFRSF14	UGGT1
HLA-A	UGGT1
CDK2	USP7
ICAM1	YBX2

Supplemental Table S2. Drug combination predictions. For each melanoma genomic subgroup-specific signaling network based on a unique combination of root gene and gene expression dataset, drug combinations and corresponding scores generated are shown.

BRAF_PrimaryMelanoma_GSE15605			NRAS_PrimaryMelanoma_GSE15605		
drug1	drug2	score	drug1	drug2	score
tretinoin	etoposide	10.04233281	tretinoin	estradiol	29.68027351
amitriptyline	tretinoin	10.03778148	dasatinib	tretinoin	25.96208168
fluticasone	tretinoin	9.536022682	tretinoin	fluvastatin	24.42506327
dasatinib	tretinoin	9.402201405	tretinoin	doxorubicin	23.80265741
tretinoin	dinoprostone	9.209737795	fluticasone	tretinoin	22.75853728
dabrafenib	tretinoin	9.153716567	tretinoin	celecoxib	22.62813817
tretinoin	vemurafenib	8.564421509	chloroquine	tretinoin	21.81070144
tretinoin	calcitriol	8.430968592	levofloxacin	tretinoin	21.30102114
tretinoin	celecoxib	7.977309093	tretinoin	dinoprostone	20.55412755
tretinoin	doxorubicin	7.571390368	tretinoin	fexofenadine	20.39244678
tretinoin	chloroquine	7.357754521	tretinoin	fluoxetine	17.8060005
tretinoin	chloramphenicol	7.185917304	dasatinib	estradiol	17.56763743
amitriptyline	etoposide	6.721116295	tretinoin	fludarabine	17.40349962
tretinoin	mifepristone	6.570910254	estradiol	doxorubicin	17.22767198
fluticasone	etoposide	6.412809204	tretinoin	gatifloxacin	17.05671464
dasatinib	etoposide	6.331696494	fluvastatin	estradiol	16.60978178
tretinoin	decitabine	6.284707015	tretinoin	methylprednisolone	16.3502235
tretinoin	bosutinib	6.225008089	celecoxib	estradiol	15.66051316
dabrafenib	etoposide	6.166516491	tretinoin	mifepristone	15.25444753
dinoprostone	etoposide	6.093212124	tretinoin	azelastine	15.14290602
vemurafenib	etoposide	5.769530451	fluticasone	estradiol	15.01418114
tretinoin	paclitaxel	5.754127992	tretinoin	palbociclib	14.9594583
calcitriol	etoposide	5.578318538	estradiol	dinoprostone	14.65428705
tretinoin	pirfenidone	5.576148856	chloroquine	estradiol	14.31899905
celecoxib	etoposide	5.3739219	dasatinib	doxorubicin	14.06778472
amitriptyline	dinoprostone	5.209175688	levofloxacin	estradiol	13.9333812
etoposide	doxorubicin	5.002395328	tretinoin	pirfenidone	13.36281088
chloroquine	etoposide	4.999631047	fexofenadine	estradiol	13.33906636
chloramphenicol	etoposide	4.887839286	tretinoin	clarithromycin	13.32240433
tretinoin	fluorometholone	4.817561576	fluvastatin	doxorubicin	13.30199233
fluticasone	dinoprostone	4.78505507	tretinoin	floxuridine	13.18772517
dinoprostone	calcitriol	4.771322449	celecoxib	doxorubicin	12.54585405
amitriptyline	calcitriol	4.763485566	tretinoin	cilostazol	12.34773437
tretinoin	fluvastatin	4.665737776	fluticasone	doxorubicin	12.01722326
dasatinib	dinoprostone	4.665345563	doxorubicin	dinoprostone	11.74607121
tretinoin	fluoxetine	4.571350299	fluoxetine	estradiol	11.58520531
dabrafenib	dinoprostone	4.529284268	chloroquine	doxorubicin	11.45972605
tretinoin	temsirolimus	4.510057646	fludarabine	estradiol	11.27075207
mifepristone	etoposide	4.469611045	estradiol	gatifloxacin	11.15637549
tretinoin	latanoprost	4.401969455	levofloxacin	doxorubicin	11.15031816
amitriptyline	doxorubicin	4.386479913	dasatinib	dinoprostone	11.10196994
fluticasone	calcitriol	4.37445742	estradiol	methylprednisolone	10.6942771
dinoprostone	doxorubicin	4.327400246	fexofenadine	doxorubicin	10.67471217
decitabine	etoposide	4.270596388	fluvastatin	dinoprostone	10.55961137
dasatinib	calcitriol	4.26462334	celecoxib	dinoprostone	10.16398543
vemurafenib	dinoprostone	4.237699444	dasatinib	celecoxib	9.998305571
etoposide	bosutinib	4.145991744	estradiol	palbociclib	9.925429505
dabrafenib	calcitriol	4.140151399	estradiol	azelastine	9.905247297
fluticasone	doxorubicin	4.053204964	estradiol	mifepristone	9.87722052
calcitriol	doxorubicin	3.960059109	fluvastatin	celecoxib	9.654087261
dasatinib	doxorubicin	3.959727076	dasatinib	fluvastatin	9.406760358

celecoxib	dinoprostone	3.947744326	fluoxetine	doxorubicin	9.270202356
paclitaxel	etoposide	3.914010074	fluticasone	dinoprostone	9.192804718
vemurafenib	calcitriol	3.873618047	fludarabine	doxorubicin	9.017761072
dabrafenib	doxorubicin	3.84619026	gatifloxacin	doxorubicin	8.927982117
fluticasone	amitriptyline	3.825479673	estradiol	pirfenidone	8.815665992
etoposide	pirfenidone	3.749863009	estradiol	clarithromycin	8.713855415
dasatinib	amitriptyline	3.637540364	chloroquine	dinoprostone	8.712254914
celecoxib	calcitriol	3.608578593	estradiol	floxuridine	8.654297895
vemurafenib	doxorubicin	3.598581445	methylprednisolone	doxorubicin	8.558184041
dabrafenib	amitriptyline	3.508802927	levofloxacin	dinoprostone	8.437355881
chloroquine	dinoprostone	3.386195477	fexofenadine	dinoprostone	8.077468664
dinoprostone	bosutinib	3.361680718	estradiol	cilostazol	7.996567112
celecoxib	doxorubicin	3.352272515	palbociclib	doxorubicin	7.945101217
amitriptyline	vemurafenib	3.282914327	azelastine	doxorubicin	7.926766466
chloramphenicol	dinoprostone	3.277679975	mifepristone	doxorubicin	7.902764549
etoposide	fluorometholone	3.276125224	fluticasone	celecoxib	7.60208222
etoposide	fluvastatin	3.173620768	chloroquine	celecoxib	7.074874531
amitriptyline	bosutinib	3.105293037	doxorubicin	pirfenidone	7.055984302
etoposide	fluoxetine	3.093991478	doxorubicin	clarithromycin	6.973335144
chloroquine	calcitriol	3.093323657	fluoxetine	dinoprostone	6.966269428
calcitriol	bosutinib	3.075016938	doxorubicin	floxuridine	6.926118267
etoposide	temsirolimus	3.067010848	levofloxacin	celecoxib	6.755824799
amitriptyline	celecoxib	3.059266572	gatifloxacin	dinoprostone	6.755163416
mifepristone	dinoprostone	2.996581294	fludarabine	dinoprostone	6.735301448
chloramphenicol	calcitriol	2.993951478	fluticasone	fluvastatin	6.636826622
etoposide	latanoprost	2.992108422	dasatinib	fluticasone	6.588803602
dasatinib	fluticasone	2.95570444	methylprednisolone	dinoprostone	6.475363748
chloroquine	doxorubicin	2.914393927	fexofenadine	celecoxib	6.467661655
decitabine	dinoprostone	2.891725788	doxorubicin	cilostazol	6.398076291
fluticasone	dabrafenib	2.840158852	palbociclib	dinoprostone	6.12142919
chloramphenicol	doxorubicin	2.825835202	chloroquine	fluvastatin	6.068894098
doxorubicin	bosutinib	2.811645685	azelastine	dinoprostone	5.998120295
dinoprostone	pirfenidone	2.798040676	dasatinib	chloroquine	5.985471005
fluticasone	bosutinib	2.75706947	mifepristone	dinoprostone	5.901118531
mifepristone	calcitriol	2.737180971	levofloxacin	fluvastatin	5.71428336
fluticasone	vemurafenib	2.657316008	dasatinib	levofloxacin	5.605510584
dasatinib	bosutinib	2.656423115	fluvastatin	fexofenadine	5.470546155
dasatinib	dabrafenib	2.641961499	fluoxetine	celecoxib	5.460431003
decitabine	calcitriol	2.641615431	celecoxib	gatifloxacin	5.40750656
paclitaxel	dinoprostone	2.624192649	dinoprostone	pirfenidone	5.39761011
mifepristone	doxorubicin	2.583583293	dasatinib	fexofenadine	5.366412976
dabrafenib	bosutinib	2.571172584	dinoprostone	clarithromycin	5.276222313
calcitriol	pirfenidone	2.55794544	dinoprostone	floxuridine	5.262773246
decitabine	doxorubicin	2.488922727	celecoxib	methylprednisolone	5.183526998
fluticasone	celecoxib	2.476762512	fludarabine	celecoxib	5.178558673
dasatinib	vemurafenib	2.471878141	celecoxib	palbociclib	5.167003519
vemurafenib	bosutinib	2.405646452	celecoxib	azelastine	4.802719051
paclitaxel	calcitriol	2.397029093	dinoprostone	cilostazol	4.778677563
dabrafenib	vemurafenib	2.370573739	fluvastatin	palbociclib	4.597878416
pirfenidone	doxorubicin	2.370094428	dasatinib	palbociclib	4.596513707
dasatinib	celecoxib	2.304103533	fluvastatin	gatifloxacin	4.572652465
paclitaxel	doxorubicin	2.262502688	celecoxib	mifepristone	4.533740348
celecoxib	bosutinib	2.241381402	fluvastatin	fluoxetine	4.517959438
amitriptyline	pirfenidone	2.236933029	dasatinib	gatifloxacin	4.485163186
dabrafenib	celecoxib	2.209721115	celecoxib	pirfenidone	4.463607909
dinoprostone	fluorometholone	2.20194441	dasatinib	fluoxetine	4.393840063
dinoprostone	fluoxetine	2.176423619	fluvastatin	methylprednisolone	4.383252659

amitriptyline	chloroquine	2.170443217	dasatinib	methylprednisolone	4.299387196
dinoprostone	fluvastatin	2.12816188	celecoxib	floxuridine	4.266908489
vemurafenib	celecoxib	2.06746439	celecoxib	clarithromycin	4.223614591
dinoprostone	temsirolimus	2.061394767	fluvastatin	fludarabine	4.196509069
amitriptyline	chloramphenicol	2.044573139	fluvastatin	azelastine	4.062286749
dinoprostone	latanoprost	2.020268159	dasatinib	fludarabine	4.047058218
calcitriol	fluorometholone	2.011373315	dasatinib	azelastine	3.984960131
calcitriol	fluoxetine	1.988782177	fluvastatin	pirfenidone	3.896852329
calcitriol	fluvastatin	1.943940059	dasatinib	pirfenidone	3.868655326
fluorometholone	doxorubicin	1.897643733	celecoxib	cilostazol	3.674172913
calcitriol	temsirolimus	1.882987785	fluvastatin	mifepristone	3.670902393
amitriptyline	mifepristone	1.868111092	fluvastatin	floxuridine	3.654468164
doxorubicin	fluoxetine	1.861250312	dasatinib	floxuridine	3.602095215
amitriptyline	decitabine	1.852297828	fluvastatin	clarithromycin	3.571539203
calcitriol	latanoprost	1.845489124	dasatinib	mifepristone	3.538956276
fluticasone	pirfenidone	1.844348393	dasatinib	clarithromycin	3.503204382
fluvastatin	doxorubicin	1.834783993	fluticasone	chloroquine	3.192383604
doxorubicin	temsirolimus	1.776517537	fluvastatin	cilostazol	2.977411463
chloroquine	bosutinib	1.766779567	dasatinib	cilostazol	2.871376501
doxorubicin	latanoprost	1.739704783	fluticasone	levofloxacin	2.845908523
dasatinib	pirfenidone	1.728335647	fluticasone	palbociclib	2.745829565
chloramphenicol	bosutinib	1.690823235	fluticasone	fexofenadine	2.72451906
dabrafenib	pirfenidone	1.660770854	chloroquine	palbociclib	2.398951129
amitriptyline	paclitaxel	1.636141558	chloroquine	levofloxacin	2.352756489
pirfenidone	bosutinib	1.61218469	fluticasone	gatifloxacin	2.274968011
vemurafenib	pirfenidone	1.553854276	chloroquine	fexofenadine	2.252401947
mifepristone	bosutinib	1.545432377	fluticasone	pirfenidone	2.184087161
fluticasone	chloroquine	1.53674381	fluticasone	methylprednisolone	2.180738566
amitriptyline	fluoxetine	1.533911371	levofloxacin	palbociclib	2.173169111
decitabine	bosutinib	1.508371174	fexofenadine	palbociclib	2.080474694
celecoxib	pirfenidone	1.448276384	fluticasone	fluoxetine	2.048379232
fluticasone	chloramphenicol	1.414540621	fluticasone	azelastine	2.023157718
amitriptyline	fluorometholone	1.382292556	levofloxacin	fexofenadine	1.937982316
paclitaxel	bosutinib	1.353442468	fluticasone	floxuridine	1.911020711
dasatinib	chloroquine	1.346579236	chloroquine	gatifloxacin	1.88008115
amitriptyline	fluvastatin	1.327519053	chloroquine	pirfenidone	1.87442707
fluticasone	decitabine	1.310778071	chloroquine	methylprednisolone	1.802207967
amitriptyline	temsirolimus	1.294061116	fluticasone	clarithromycin	1.776898091
fluticasone	mifepristone	1.291781192	gatifloxacin	palbociclib	1.737733288
amitriptyline	latanoprost	1.284189506	fluticasone	fludarabine	1.721855789
dabrafenib	chloroquine	1.270048003	chloroquine	azelastine	1.672575703
dasatinib	chloramphenicol	1.225248294	levofloxacin	pirfenidone	1.67099216
vemurafenib	chloroquine	1.188285256	methylprednisolone	palbociclib	1.665756169
bosutinib	fluoxetine	1.183262475	chloroquine	fluoxetine	1.636382879
fluticasone	fluoxetine	1.167653586	levofloxacin	gatifloxacin	1.617067987
dabrafenib	chloramphenicol	1.151719005	palbociclib	pirfenidone	1.612230203
dasatinib	decitabine	1.14827047	fluoxetine	palbociclib	1.610230524
fluorometholone	bosutinib	1.138898725	chloroquine	floxuridine	1.60559575
fluticasone	paclitaxel	1.131486864	fexofenadine	pirfenidone	1.599717613
dasatinib	mifepristone	1.118619333	levofloxacin	methylprednisolone	1.55008884
chloroquine	celecoxib	1.108588744	fexofenadine	gatifloxacin	1.548093523
fluvastatin	bosutinib	1.097833096	azelastine	palbociclib	1.544906951
dabrafenib	decitabine	1.082926488	fluticasone	mifepristone	1.499772785
vemurafenib	chloramphenicol	1.077574004	fexofenadine	methylprednisolone	1.483971306
temsirolimus	bosutinib	1.066203062	chloroquine	clarithromycin	1.468465751
dasatinib	fluoxetine	1.058307273	levofloxacin	azelastine	1.439095779
dabrafenib	mifepristone	1.051406916	palbociclib	floxuridine	1.43850397

bosutinib	latanoprost	1.050406896	levofloxacin	floxuridine	1.403233244
vemurafenib	decitabine	1.013210191	fludarabine	palbociclib	1.398899027
dabrafenib	fluoxetine	1.007763039	fexofenadine	azelastine	1.377712547
chloramphenicol	celecoxib	1.005475837	levofloxacin	fluoxetine	1.359687797
vemurafenib	mifepristone	0.983719775	palbociclib	clarithromycin	1.357282805
dasatinib	paclitaxel	0.979860981	fexofenadine	floxuridine	1.343379693
fluticasone	fluorometholone	0.961622185	gatifloxacin	pirfenidone	1.335761034
celecoxib	decitabine	0.945259408	chloroquine	fludarabine	1.319376367
vemurafenib	fluoxetine	0.94288559	fexofenadine	fluoxetine	1.301691635
dabrafenib	paclitaxel	0.920999345	methylprednisolone	pirfenidone	1.280433654
fluticasone	fluvastatin	0.918445806	levofloxacin	clarithromycin	1.263035351
celecoxib	mifepristone	0.917904857	gatifloxacin	methylprednisolone	1.23822875
fluticasone	latanoprost	0.902939462	fluticasone	cilostazol	1.221651872
fluticasone	temsirolimus	0.900242045	mifepristone	palbociclib	1.220250983
chloroquine	pirfenidone	0.898604431	fexofenadine	clarithromycin	1.209161805
celecoxib	fluoxetine	0.87922072	fluoxetine	pirfenidone	1.202718081
vemurafenib	paclitaxel	0.861707542	azelastine	pirfenidone	1.187909119
dasatinib	fluorometholone	0.835266908	gatifloxacin	azelastine	1.149574398
chloramphenicol	pirfenidone	0.827146634	chloroquine	mifepristone	1.146999977
celecoxib	paclitaxel	0.804055145	floxuridine	pirfenidone	1.122067206
dasatinib	fluvastatin	0.795540361	gatifloxacin	floxuridine	1.121279659
dasatinib	latanoprost	0.788488009	methylprednisolone	azelastine	1.101958891
dabrafenib	fluorometholone	0.785784602	fluoxetine	gatifloxacin	1.085359485
dasatinib	temsirolimus	0.781951999	methylprednisolone	floxuridine	1.074836123
decitabine	pirfenidone	0.766471923	levofloxacin	fludarabine	1.047109705
mifepristone	pirfenidone	0.755363578	clarithromycin	pirfenidone	1.04331631
dabrafenib	fluvastatin	0.747798595	fluoxetine	methylprednisolone	1.040403767
dabrafenib	latanoprost	0.742932785	fludarabine	pirfenidone	1.010997894
dabrafenib	temsirolimus	0.735628138	gatifloxacin	clarithromycin	1.008927129
vemurafenib	fluorometholone	0.73519761	fexofenadine	fludarabine	1.002446258
vemurafenib	fluvastatin	0.699657055	azelastine	floxuridine	0.997559178
vemurafenib	latanoprost	0.695104494	palbociclib	cilostazol	0.992514952
vemurafenib	temsirolimus	0.688270103	fluoxetine	floxuridine	0.97255774
celecoxib	fluorometholone	0.685978408	methylprednisolone	clarithromycin	0.967137248
pirfenidone	fluoxetine	0.682780487	fluoxetine	azelastine	0.966602699
paclitaxel	pirfenidone	0.661632149	chloroquine	cilostazol	0.936093847
celecoxib	fluvastatin	0.652844501	levofloxacin	mifepristone	0.908292204
celecoxib	latanoprost	0.648517884	azelastine	clarithromycin	0.89789243
celecoxib	temsirolimus	0.642192552	mifepristone	pirfenidone	0.880600534
fluorometholone	pirfenidone	0.562304498	clarithromycin	floxuridine	0.875792396
pirfenidone	fluvastatin	0.537057293	fexofenadine	mifepristone	0.869549882
pirfenidone	latanoprost	0.527990024	fluoxetine	clarithromycin	0.847736402
pirfenidone	temsirolimus	0.526412722	fludarabine	gatifloxacin	0.835021171
chloroquine	fluoxetine	0.36001794	fludarabine	methylprednisolone	0.800434495
decitabine	fluoxetine	0.306398405	fludarabine	floxuridine	0.780670609
chloramphenicol	fluoxetine	0.299553978	fludarabine	azelastine	0.744390786
mifepristone	fluoxetine	0.272893828	levofloxacin	cilostazol	0.742921411
paclitaxel	fluoxetine	0.239139816	gatifloxacin	mifepristone	0.724285313
fluorometholone	fluoxetine	0.20884137	pirfenidone	cilostazol	0.717300182
latanoprost	fluoxetine	0.205464394	fexofenadine	cilostazol	0.71123282
temsirolimus	fluoxetine	0.19551107	methylprednisolone	mifepristone	0.694285329
fluvastatin	fluoxetine	0.194497133	mifepristone	floxuridine	0.678533656
chloroquine	decitabine	0.172803314	fludarabine	clarithromycin	0.652205885
chloroquine	chloramphenicol	0.105482788	mifepristone	azelastine	0.645705358
chloroquine	latanoprost	0.104450092	gatifloxacin	cilostazol	0.592445188
chloroquine	mifepristone	0.094600003	fluoxetine	fludarabine	0.58173502
chloramphenicol	decitabine	0.088067155	methylprednisolone	cilostazol	0.567906039

decitabine	latanoprost	0.087996536	mifepristone	clarithromycin	0.565713972
chloroquine	fluorometholone	0.085254491	floxuridine	cilostazol	0.553883616
chloroquine	paclitaxel	0.083144648	azelastine	cilostazol	0.528143183
chloroquine	temsirolimus	0.079812715	fluoxetine	mifepristone	0.501601592
mifepristone	decitabine	0.078944298	clarithromycin	cilostazol	0.462738254
decitabine	fluorometholone	0.071467515	fluoxetine	cilostazol	0.412739372
decitabine	paclitaxel	0.069390885	fludarabine	mifepristone	0.27185373
chloroquine	fluvastatin	0.068488824	fludarabine	cilostazol	0.226853568
decitabine	temsirolimus	0.066905759	mifepristone	cilostazol	0.192879462
decitabine	fluvastatin	0.057181044			
chloramphenicol	latanoprost	0.045015825			
mifepristone	latanoprost	0.040043291			
fluorometholone	latanoprost	0.038955125			
temsirolimus	latanoprost	0.036468628			
paclitaxel	latanoprost	0.035249129			
fluvastatin	latanoprost	0.029228284			
fluorometholone	temsirolimus	0.021702504			
chloramphenicol	fluorometholone	0.020063778			
chloramphenicol	temsirolimus	0.018783112			
mifepristone	fluorometholone	0.017104854			
mifepristone	temsirolimus	0.016013055			
paclitaxel	fluorometholone	0.015181961			
paclitaxel	temsirolimus	0.0142129			
fluorometholone	fluvastatin	0.013027193			
fluvastatin	temsirolimus	0.01219567			
chloramphenicol	mifepristone	0.005574798			
chloramphenicol	fluvastatin	0.005282208			
chloramphenicol	paclitaxel	0.005187039			
mifepristone	fluvastatin	0.003619656			
paclitaxel	fluvastatin	0.003367888			
mifepristone	paclitaxel	0.003249525			

**NF1_PrimaryMelanoma_GSE15605**			**TWT_PrimaryMelanoma_GSE15605**		
drug1	drug2	score	drug1	drug2	score
tretinoin	dexamethasone	24.69211945	tretinoin	brimonidine	1.039750252
tretinoin	sirolimus	20.92964374	tretinoin	cilostazol	0.869498749
tretinoin	bosutinib	19.53833587	brimonidine	cilostazol	0.734831402
dexamethasone	sirolimus	18.44093396	tretinoin	progesterone	0.617019295
dexamethasone	bosutinib	17.05878992	tretinoin	fluvastatin	0.581468943
tretinoin	celecoxib	16.61594953	tretinoin	imiquimod	0.539935447
tretinoin	doxorubicin	16.59050939	brimonidine	progesterone	0.522568779
tretinoin	naproxen	14.85546126	tretinoin	erythromycin	0.481096328
tretinoin	fludarabine	14.81503927	brimonidine	fluvastatin	0.473418362
dexamethasone	doxorubicin	14.6445745	tretinoin	estradiol	0.45340733
bosutinib	sirolimus	14.59031975	brimonidine	imiquimod	0.439602765
dexamethasone	celecoxib	14.49095053	cilostazol	progesterone	0.437001962
tretinoin	indinavir	13.83134849	fluvastatin	cilostazol	0.395899566
tretinoin	capsaicin	13.82667055	brimonidine	erythromycin	0.391697335
tretinoin	pirfenidone	13.70812207	brimonidine	estradiol	0.369153604
tretinoin	propranolol	13.64380425	imiquimod	cilostazol	0.367621025
naproxen	dexamethasone	12.75808163	cilostazol	erythromycin	0.32755976
fludarabine	dexamethasone	12.68836523	cilostazol	estradiol	0.3087074
celecoxib	sirolimus	12.40783064	fluvastatin	progesterone	0.292240868
sirolimus	doxorubicin	12.3908477	imiquimod	progesterone	0.27136652
tretinoin	idarubicin	12.19581682	erythromycin	progesterone	0.241794527
dexamethasone	capsaicin	11.99922451	estradiol	progesterone	0.227878296

dexamethasone	indinavir	11.85967396	fluvastatin	imiquimod	0.076210373
dexamethasone	pirfenidone	11.76258734	fluvastatin	erythromycin	0.067905397
bosutinib	doxorubicin	11.75205642	fluvastatin	estradiol	0.063997172
dexamethasone	propranolol	11.74903354	imiquimod	erythromycin	0.063055011
tretinoin	azelastine	11.35608495	imiquimod	estradiol	0.059425946
tretinoin	salmeterol	11.32215856	erythromycin	estradiol	0.052950042
naproxen	sirolimus	11.09093666			
tretinoin	olopatadine	11.06580485			
fludarabine	sirolimus	11.060357			
bosutinib	celecoxib	10.54478074			
tretinoin	fluoxetine	10.54425581			
tretinoin	prazosin	10.48916608			
dexamethasone	idarubicin	10.459148			
indinavir	sirolimus	10.32612809			
sirolimus	capsaicin	10.32428047			
pirfenidone	sirolimus	10.23422865			
propranolol	sirolimus	10.18668719			
tretinoin	gatifloxacin	10.05721387			
celecoxib	doxorubicin	10.01359196			
tretinoin	telmisartan	9.836469568			
dexamethasone	azelastine	9.72842259			
dexamethasone	salmeterol	9.6556257			
dexamethasone	olopatadine	9.503379148			
tretinoin	clozapine	9.431452145			
tretinoin	cetirizine	9.368770087			
fludarabine	doxorubicin	9.202755879			
tretinoin	dactinomycin	9.190212676			
naproxen	doxorubicin	9.186340118			
idarubicin	sirolimus	9.105103622			
dexamethasone	fluoxetine	9.005097262			
dexamethasone	prazosin	8.993943928			
tretinoin	naloxone	8.891602286			
dexamethasone	gatifloxacin	8.625079393			
indinavir	doxorubicin	8.575391732			
pirfenidone	doxorubicin	8.488847728			
azelastine	sirolimus	8.478058893			
tretinoin	fluticasone	8.458202977			
salmeterol	sirolimus	8.452229566			
capsaicin	doxorubicin	8.402636334			
propranolol	doxorubicin	8.399757203			
dexamethasone	telmisartan	8.388375821			
bosutinib	capsaicin	8.36293697			
sirolimus	olopatadine	8.261616588			
tretinoin	gemfibrozil	8.08604987			
dexamethasone	clozapine	8.079957895			
naproxen	bosutinib	8.052997456			
dexamethasone	cetirizine	8.025948637			
dexamethasone	dactinomycin	7.881538066			
sirolimus	fluoxetine	7.871656287			
sirolimus	prazosin	7.830941125			
fludarabine	bosutinib	7.787519248			
dexamethasone	naloxone	7.627947238			
bosutinib	propranolol	7.615650375			
idarubicin	doxorubicin	7.559168108			
sirolimus	gatifloxacin	7.508474077			
indinavir	bosutinib	7.36642004			
bosutinib	pirfenidone	7.360453414			

sirolimus	telmisartan	7.343128699
dexamethasone	fluticasone	7.257766656
salmeterol	doxorubicin	7.081872135
azelastine	doxorubicin	7.051194719
sirolimus	clozapine	7.041198403
sirolimus	cetirizine	6.994398587
celecoxib	capsaicin	6.978894256
dexamethasone	gemfibrozil	6.943285452
sirolimus	dactinomycin	6.861191829
olopatadine	doxorubicin	6.842995676
sirolimus	naloxone	6.638285109
naproxen	celecoxib	6.608181641
fluoxetine	doxorubicin	6.58007259
bosutinib	idarubicin	6.508289208
prazosin	doxorubicin	6.503225148
fludarabine	celecoxib	6.357663092
sirolimus	fluticasone	6.314736826
propranolol	celecoxib	6.27874045
gatifloxacin	doxorubicin	6.233626781
telmisartan	doxorubicin	6.152878874
sirolimus	gemfibrozil	6.036949858
pirfenidone	celecoxib	6.030421659
indinavir	celecoxib	6.027157936
bosutinib	olopatadine	5.998025026
bosutinib	azelastine	5.986610944
clozapine	doxorubicin	5.855787623
cetirizine	doxorubicin	5.817235643
dactinomycin	doxorubicin	5.696245162
bosutinib	salmeterol	5.664398218
bosutinib	prazosin	5.586553763
naloxone	doxorubicin	5.508206224
bosutinib	gatifloxacin	5.367025223
bosutinib	fluoxetine	5.364799617
idarubicin	celecoxib	5.326806727
doxorubicin	fluticasone	5.237799662
doxorubicin	gemfibrozil	5.001598527
bosutinib	clozapine	4.974165148
bosutinib	cetirizine	4.938954029
celecoxib	olopatadine	4.921813336
bosutinib	telmisartan	4.919429698
bosutinib	dactinomycin	4.904350635
celecoxib	azelastine	4.889808865
bosutinib	naloxone	4.762379105
naproxen	capsaicin	4.627850957
salmeterol	celecoxib	4.584652333
celecoxib	prazosin	4.570902124
bosutinib	fluticasone	4.541556362
propranolol	capsaicin	4.507723692
celecoxib	gatifloxacin	4.392722135
bosutinib	gemfibrozil	4.375508524
celecoxib	fluoxetine	4.355182913
fludarabine	capsaicin	4.329684148
pirfenidone	capsaicin	4.187669319
indinavir	capsaicin	4.154732198
celecoxib	clozapine	4.063151312
celecoxib	cetirizine	4.034092313
celecoxib	dactinomycin	4.014039193

celecoxib	telmisartan	3.981444078
celecoxib	naloxone	3.900208839
celecoxib	fluticasone	3.720898471
idarubicin	capsaicin	3.678608493
celecoxib	gemfibrozil	3.589429476
capsaicin	olopatadine	3.446529964
azelastine	capsaicin	3.33907892
naproxen	propranolol	3.219677222
capsaicin	prazosin	3.150957
capsaicin	gatifloxacin	3.033544444
salmeterol	capsaicin	2.972287463
fluoxetine	capsaicin	2.873103878
pirfenidone	propranolol	2.848087682
fludarabine	propranolol	2.786737891
capsaicin	clozapine	2.775711403
capsaicin	dactinomycin	2.772031992
indinavir	propranolol	2.768851839
capsaicin	cetirizine	2.754740109
capsaicin	naloxone	2.702342094
naproxen	pirfenidone	2.619006963
capsaicin	fluticasone	2.583881069
capsaicin	telmisartan	2.580285386
naproxen	indinavir	2.515193257
capsaicin	gemfibrozil	2.509794652
fludarabine	naproxen	2.476583244
propranolol	idarubicin	2.463966699
propranolol	olopatadine	2.397221265
naproxen	idarubicin	2.245141339
naproxen	olopatadine	2.233372365
indinavir	pirfenidone	2.173388229
propranolol	azelastine	2.166212258
fludarabine	pirfenidone	2.120604297
propranolol	prazosin	2.100039302
propranolol	gatifloxacin	2.031896709
fludarabine	indinavir	1.999194607
pirfenidone	olopatadine	1.94960752
pirfenidone	idarubicin	1.942478882
naproxen	azelastine	1.934938777
naproxen	prazosin	1.907727574
indinavir	olopatadine	1.872230096
propranolol	dactinomycin	1.856733199
naproxen	gatifloxacin	1.851443568
indinavir	idarubicin	1.849695226
fludarabine	olopatadine	1.843314627
propranolol	naloxone	1.826674661
propranolol	clozapine	1.802855643
fludarabine	idarubicin	1.792976748
propranolol	cetirizine	1.787125113
propranolol	fluticasone	1.757330697
propranolol	gemfibrozil	1.738832638
naproxen	dactinomycin	1.691836364
propranolol	fluoxetine	1.673777915
naproxen	naloxone	1.673637679
idarubicin	olopatadine	1.671234081
pirfenidone	azelastine	1.660367747
pirfenidone	prazosin	1.648501671
propranolol	salmeterol	1.629743284

naproxen	gemfibrozil	1.616338914
naproxen	fluticasone	1.615983019
naproxen	clozapine	1.611589033
pirfenidone	gatifloxacin	1.601854623
naproxen	cetirizine	1.596324491
indinavir	azelastine	1.568573399
indinavir	prazosin	1.567895772
indinavir	gatifloxacin	1.525341087
fludarabine	prazosin	1.51644493
fludarabine	azelastine	1.497881688
fludarabine	gatifloxacin	1.478568503
pirfenidone	dactinomycin	1.463763707
pirfenidone	naloxone	1.451261948
azelastine	olopatadine	1.440202114
olopatadine	prazosin	1.420052149
propranolol	telmisartan	1.412948432
pirfenidone	gemfibrozil	1.409713959
idarubicin	azelastine	1.406023317
pirfenidone	fluticasone	1.403331618
idarubicin	prazosin	1.402998841
indinavir	dactinomycin	1.393846166
naproxen	fluoxetine	1.38652877
indinavir	naloxone	1.384892051
pirfenidone	clozapine	1.383338753
olopatadine	gatifloxacin	1.378174076
pirfenidone	cetirizine	1.369803391
idarubicin	gatifloxacin	1.364506746
indinavir	gemfibrozil	1.352629768
fludarabine	dactinomycin	1.351105701
fludarabine	naloxone	1.34776512
indinavir	fluticasone	1.34102678
fludarabine	gemfibrozil	1.329703671
fludarabine	fluticasone	1.30845797
indinavir	clozapine	1.307260796
indinavir	cetirizine	1.294073054
naproxen	salmeterol	1.285320573
olopatadine	dactinomycin	1.259365966
fludarabine	clozapine	1.249076623
idarubicin	dactinomycin	1.246876854
olopatadine	naloxone	1.245848558
idarubicin	naloxone	1.238195118
fludarabine	cetirizine	1.235752393
idarubicin	gemfibrozil	1.207672326
olopatadine	gemfibrozil	1.203269103
olopatadine	fluticasone	1.202949321
olopatadine	clozapine	1.199532303
idarubicin	fluticasone	1.1985508
azelastine	prazosin	1.189798456
olopatadine	cetirizine	1.188166744
idarubicin	clozapine	1.171698389
idarubicin	cetirizine	1.159969236
azelastine	gatifloxacin	1.159469465
prazosin	gatifloxacin	1.156975348
pirfenidone	fluoxetine	1.150696854
naproxen	telmisartan	1.113089103
azelastine	dactinomycin	1.059515201
prazosin	dactinomycin	1.057236094

azelastine	naloxone	1.055903373
indinavir	fluoxetine	1.051236478
prazosin	naloxone	1.050436981
pirfenidone	salmeterol	1.041573486
azelastine	gemfibrozil	1.03928463
fluoxetine	olopatadine	1.031659879
gatifloxacin	dactinomycin	1.028230202
prazosin	gemfibrozil	1.02594766
azelastine	fluticasone	1.024482227
gatifloxacin	naloxone	1.021070856
prazosin	fluticasone	1.017160605
gatifloxacin	gemfibrozil	0.995900401
prazosin	clozapine	0.991585915
gatifloxacin	fluticasone	0.988378384
azelastine	clozapine	0.982869135
prazosin	cetirizine	0.981583726
azelastine	cetirizine	0.972521006
gatifloxacin	clozapine	0.96623469
gatifloxacin	cetirizine	0.956562309
salmeterol	olopatadine	0.956128004
idarubicin	fluoxetine	0.950523293
fludarabine	fluoxetine	0.938484156
dactinomycin	naloxone	0.933047506
indinavir	salmeterol	0.92771481
dactinomycin	gemfibrozil	0.910046918
dactinomycin	fluticasone	0.903173351
pirfenidone	telmisartan	0.901492879
naloxone	gemfibrozil	0.900618892
naloxone	fluticasone	0.8954595
clozapine	dactinomycin	0.882938596
clozapine	naloxone	0.879807157
cetirizine	dactinomycin	0.874100041
cetirizine	naloxone	0.871120283
fluticasone	gemfibrozil	0.869823507
clozapine	gemfibrozil	0.865657276
cetirizine	gemfibrozil	0.85740982
clozapine	fluticasone	0.853549443
cetirizine	fluticasone	0.845197837
salmeterol	idarubicin	0.844490818
olopatadine	telmisartan	0.828003342
clozapine	cetirizine	0.810867037
indinavir	telmisartan	0.80245075
fluoxetine	prazosin	0.7974772
fluoxetine	gatifloxacin	0.783843853
fludarabine	salmeterol	0.783264297
azelastine	fluoxetine	0.750907519
fluoxetine	gemfibrozil	0.740374205
idarubicin	telmisartan	0.73058481
fluoxetine	naloxone	0.72470264
fluoxetine	dactinomycin	0.716271107
fluoxetine	fluticasone	0.710004442
salmeterol	prazosin	0.703834814
salmeterol	gatifloxacin	0.696404751
salmeterol	gemfibrozil	0.683533531
fludarabine	telmisartan	0.676544562
salmeterol	naloxone	0.651271671
salmeterol	fluticasone	0.642673002

salmeterol	dactinomycin	0.636369858
salmeterol	azelastine	0.635781336
fluoxetine	clozapine	0.627595952
fluoxetine	cetirizine	0.619498703
telmisartan	prazosin	0.608801326
telmisartan	gatifloxacin	0.602472782
telmisartan	gemfibrozil	0.591883939
telmisartan	naloxone	0.563584536
telmisartan	fluticasone	0.556240287
telmisartan	dactinomycin	0.550535473
azelastine	telmisartan	0.549360981
salmeterol	clozapine	0.532464356
salmeterol	cetirizine	0.524519602
telmisartan	clozapine	0.460111892
telmisartan	cetirizine	0.453222809
salmeterol	fluoxetine	0.19356083
fluoxetine	telmisartan	0.165081952
salmeterol	telmisartan	0.006055737

**BRAF_PrimaryMelanoma_TCGASKCM**			**NRAS_PrimaryMelanoma_TCGASKCM**		
drug1	drug2	score	drug1	drug2	score
etoposide	tretinoin	26.47989847	tretinoin	estradiol	24.29600728
chloroquine	tretinoin	21.45722009	chloroquine	tretinoin	21.23708644
tretinoin	estradiol	21.25679196	tretinoin	doxorubicin	20.73788428
capsaicin	tretinoin	21.08266574	levofloxacin	tretinoin	20.71673762
diazoxide	tretinoin	20.63916133	tretinoin	bosutinib	20.38013371
pirfenidone	tretinoin	20.57552872	tretinoin	pirfenidone	19.97041109
levofloxacin	tretinoin	20.4623552	diazoxide	tretinoin	19.54477548
teniposide	tretinoin	20.45605773	tretinoin	olopatadine	19.23532311
olopatadine	tretinoin	20.23958374	tretinoin	celecoxib	18.72191984
fludarabine	tretinoin	20.12901111	tretinoin	prazosin	18.6593205
mifepristone	tretinoin	20.00676871	tretinoin	omeprazole	17.18699304
nifedipine	tretinoin	19.44956479	tretinoin	fluoxetine	16.68956719
prazosin	tretinoin	19.1982856	tretinoin	chloramphenicol	16.68055458
etoposide	estradiol	18.72443467	tretinoin	calcitriol	16.67005075
chloramphenicol	tretinoin	18.55603268	tretinoin	capsaicin	16.22835269
fluoxetine	tretinoin	18.17473385	tretinoin	etoposide	16.22565637
tretinoin	bosutinib	17.93788711	tretinoin	clozapine	15.26135589
gatifloxacin	tretinoin	17.43684715	tretinoin	azelastine	13.92007922
latanoprost	tretinoin	17.24154341	tretinoin	latanoprost	13.81776406
tretinoin	panobinostat	16.18040251	tretinoin	cetirizine	13.78937425
tretinoin	miglitol	15.50504381	tretinoin	vinblastine	13.66108805
etoposide	capsaicin	15.31216757	estradiol	doxorubicin	13.58568754
chloroquine	estradiol	14.94181104	tretinoin	palbociclib	13.47982065
tretinoin	calcitriol	14.86455511	tretinoin	panobinostat	13.28717056
capsaicin	estradiol	14.78020742	bosutinib	estradiol	12.87928226
diazoxide	estradiol	14.40439307	chloroquine	estradiol	12.56689857
pirfenidone	estradiol	14.33844192	levofloxacin	estradiol	12.05530175
chloroquine	etoposide	14.33190228	tretinoin	formoterol	11.95950468
teniposide	estradiol	14.20955931	pirfenidone	estradiol	11.78817193
levofloxacin	estradiol	14.20330585	tretinoin	eplerenone	11.77725503
diazoxide	etoposide	14.18531701	olopatadine	estradiol	11.72665457
olopatadine	estradiol	14.1235846	celecoxib	estradiol	11.52815102
fludarabine	estradiol	14.04755688	tretinoin	lamivudine	11.23164327
tretinoin	omeprazole	14.00436944	diazoxide	estradiol	11.17719362
olopatadine	etoposide	13.88666518	bosutinib	doxorubicin	11.00894825

mifepristone	estradiol	13.88122766	tretinoin	bepridil	10.92216411
pirfenidone	etoposide	13.87444611	prazosin	estradiol	10.87675569
tretinoin	azelastine	13.8328071	chloroquine	doxorubicin	10.76048694
etoposide	fludarabine	13.82483878	estradiol	calcitriol	10.60007311
tretinoin	decitabine	13.81958931	levofloxacin	doxorubicin	10.32715618
etoposide	bosutinib	13.62531672	estradiol	etoposide	10.18391093
nifedipine	estradiol	13.45431581	pirfenidone	doxorubicin	10.09437454
tretinoin	amitriptyline	13.40445115	olopatadine	doxorubicin	10.03303217
tretinoin	irbesartan	13.40234997	celecoxib	doxorubicin	9.860618362
tretinoin	vinblastine	13.40104673	omeprazole	estradiol	9.828833754
prazosin	estradiol	13.32090777	fluoxetine	estradiol	9.716495538
teniposide	etoposide	13.22804984	chloramphenicol	estradiol	9.684933926
levofloxacin	etoposide	13.10032203	diazoxide	doxorubicin	9.57955504
tretinoin	lamivudine	13.06188294	capsaicin	estradiol	9.459713579
tretinoin	clozapine	12.90096253	prazosin	doxorubicin	9.31711541
chloramphenicol	estradiol	12.87217959	doxorubicin	calcitriol	9.059305643
tretinoin	nilotinib	12.76428583	doxorubicin	etoposide	8.706519613
tretinoin	pimozide	12.74463567	clozapine	estradiol	8.690245227
mifepristone	etoposide	12.73613752	chloroquine	bosutinib	8.436509854
bosutinib	estradiol	12.62368412	omeprazole	doxorubicin	8.423926178
fluoxetine	estradiol	12.61028653	celecoxib	bosutinib	8.385225834
tretinoin	azacitidine	12.36576033	olopatadine	bosutinib	8.367171095
prazosin	etoposide	12.22924187	fluoxetine	doxorubicin	8.323511613
gatifloxacin	estradiol	12.13466614	chloramphenicol	doxorubicin	8.297095515
tretinoin	formoterol	11.98418311	bosutinib	calcitriol	8.173310701
latanoprost	estradiol	11.96352388	capsaicin	doxorubicin	8.103265867
nifedipine	etoposide	11.88154223	estradiol	azelastine	8.100250074
chloramphenicol	etoposide	11.78174617	estradiol	vinblastine	8.060783401
etoposide	calcitriol	11.75186913	estradiol	cetirizine	8.024191388
tretinoin	icosapent	11.66406895	estradiol	latanoprost	7.87372295
etoposide	fluoxetine	11.57202928	pirfenidone	bosutinib	7.871796666
etoposide	gatifloxacin	11.55302259	estradiol	palbociclib	7.859609851
etoposide	panobinostat	11.53521457	levofloxacin	bosutinib	7.800365421
tretinoin	vemurafenib	11.48785724	estradiol	panobinostat	7.723526942
panobinostat	estradiol	11.32597204	bosutinib	etoposide	7.673787406
tretinoin	fluvastatin	11.10007746	clozapine	doxorubicin	7.448981754
tretinoin	eplerenone	11.01316544	chloroquine	calcitriol	7.196903449
etoposide	latanoprost	10.98676906	prazosin	bosutinib	7.065097446
tretinoin	estrone	10.78156198	olopatadine	calcitriol	7.050761542
miglitol	estradiol	10.76025372	celecoxib	calcitriol	7.039557251
tretinoin	mebendazole	10.68199377	estradiol	formoterol	6.959369782
estradiol	calcitriol	10.49801831	diazoxide	bosutinib	6.945584549
etoposide	decitabine	10.46166879	azelastine	doxorubicin	6.939067088
etoposide	miglitol	9.900666917	estradiol	eplerenone	6.905156418
estradiol	decitabine	9.722596663	vinblastine	doxorubicin	6.902632568
omeprazole	estradiol	9.719855826	cetirizine	doxorubicin	6.873911528
azelastine	estradiol	9.601611744	latanoprost	doxorubicin	6.748954074
amitriptyline	estradiol	9.329555828	palbociclib	doxorubicin	6.732555977
vinblastine	estradiol	9.310706766	olopatadine	celecoxib	6.72747942
irbesartan	estradiol	9.308943622	pirfenidone	calcitriol	6.722538622
capsaicin	bosutinib	9.266429011	levofloxacin	calcitriol	6.705695239
lamivudine	estradiol	9.068966871	panobinostat	doxorubicin	6.616546655
clozapine	estradiol	8.980351602	estradiol	lamivudine	6.534126833
etoposide	omeprazole	8.955393743	celecoxib	etoposide	6.482509702
etoposide	amitriptyline	8.895171538	olopatadine	etoposide	6.440355988
nilotinib	estradiol	8.860578484	chloroquine	etoposide	6.399911975
etoposide	azelastine	8.855980938	etoposide	calcitriol	6.399071244

pimozide	estradiol	8.816141763	estradiol	bepridil	6.381912366
etoposide	vinblastine	8.68854408	chloroquine	celecoxib	6.376489993
etoposide	azacitidine	8.65930794	fluoxetine	bosutinib	6.293838346
etoposide	irbesartan	8.656294896	chloramphenicol	bosutinib	6.234959691
estradiol	azacitidine	8.643183982	capsaicin	bosutinib	6.144644609
etoposide	clozapine	8.576261665	omeprazole	bosutinib	6.107704509
diazoxide	capsaicin	8.463401205	prazosin	calcitriol	6.068629754
etoposide	lamivudine	8.393030782	diazoxide	calcitriol	6.023155559
capsaicin	calcitriol	8.358155399	chloroquine	olopatadine	6.018490633
formoterol	estradiol	8.318447041	pirfenidone	etoposide	5.963636125
diazoxide	bosutinib	8.268003227	doxorubicin	formoterol	5.961733695
olopatadine	capsaicin	8.264148548	pirfenidone	celecoxib	5.915477647
fludarabine	capsaicin	8.239688694	doxorubicin	eplerenone	5.914068427
chloroquine	capsaicin	8.186307529	levofloxacin	etoposide	5.862288438
chloroquine	bosutinib	8.180690936	levofloxacin	celecoxib	5.657023559
etoposide	nilotinib	8.179926213	doxorubicin	lamivudine	5.597489899
icosapent	estradiol	8.093467142	pirfenidone	olopatadine	5.567240517
olopatadine	bosutinib	8.083948702	doxorubicin	bepridil	5.466437727
fludarabine	bosutinib	8.053801923	fluoxetine	calcitriol	5.409348337
pirfenidone	capsaicin	8.043640663	bosutinib	vinblastine	5.378276224
pirfenidone	bosutinib	7.975788916	chloramphenicol	calcitriol	5.365748131
estradiol	vemurafenib	7.974520636	clozapine	bosutinib	5.344622781
etoposide	pimozide	7.785420464	prazosin	etoposide	5.315033511
estradiol	fluvastatin	7.732362947	omeprazole	calcitriol	5.296552666
chloroquine	calcitriol	7.71740059	capsaicin	calcitriol	5.277998412
diazoxide	calcitriol	7.675154999	bosutinib	azelastine	5.241254999
etoposide	formoterol	7.672462748	levofloxacin	olopatadine	5.226892204
estradiol	eplerenone	7.641088273	bosutinib	cetirizine	5.192041337
olopatadine	calcitriol	7.511423348	diazoxide	etoposide	5.163963372
estradiol	estrone	7.503114175	prazosin	celecoxib	5.146890612
bosutinib	calcitriol	7.487780098	bosutinib	palbociclib	5.108273795
pirfenidone	calcitriol	7.483132805	bosutinib	panobinostat	4.985183339
fludarabine	calcitriol	7.479234955	bosutinib	latanoprost	4.850662646
etoposide	fluvastatin	7.448599947	diazoxide	celecoxib	4.794658785
etoposide	icosapent	7.433012317	prazosin	olopatadine	4.766899304
estradiol	mebendazole	7.421577857	fluoxetine	etoposide	4.731398415
teniposide	bosutinib	7.36451759	chloramphenicol	etoposide	4.679644917
etoposide	vemurafenib	7.361933591	clozapine	calcitriol	4.645367388
capsaicin	panobinostat	7.245713424	capsaicin	etoposide	4.622581962
levofloxacin	bosutinib	7.235188404	vinblastine	calcitriol	4.593856282
teniposide	capsaicin	7.163089149	fluoxetine	celecoxib	4.570199337
etoposide	estrone	7.143471986	bosutinib	eplerenone	4.543724079
capsaicin	decitabine	7.086728025	omeprazole	etoposide	4.54100906
teniposide	calcitriol	7.08307258	azelastine	calcitriol	4.505719514
etoposide	eplerenone	7.009169116	bosutinib	formoterol	4.503050069
levofloxacin	calcitriol	7.002187906	chloroquine	pirfenidone	4.497075257
mifepristone	bosutinib	7.001700921	chloramphenicol	celecoxib	4.494965907
levofloxacin	capsaicin	6.971066194	capsaicin	celecoxib	4.476345004
etoposide	mebendazole	6.925651078	cetirizine	calcitriol	4.463412288
panobinostat	bosutinib	6.895600683	palbociclib	calcitriol	4.387254659
mifepristone	calcitriol	6.800587889	diazoxide	olopatadine	4.31095702
prazosin	bosutinib	6.726524582	panobinostat	calcitriol	4.287830218
mifepristone	capsaicin	6.709001935	fluoxetine	olopatadine	4.225528868
diazoxide	olopatadine	6.671961886	bosutinib	lamivudine	4.225424402
diazoxide	fludarabine	6.660596771	omeprazole	celecoxib	4.216255503
gatifloxacin	bosutinib	6.554489296	latanoprost	calcitriol	4.214457508
prazosin	calcitriol	6.530674709	bosutinib	bepridil	4.16766748

capsaicin	gatifloxacin	6.514595705	olopatadine	capsaicin	4.145859606
bosutinib	decitabine	6.498058951	chloramphenicol	olopatadine	4.140029374
olopatadine	fludarabine	6.489684031	vinblastine	etoposide	4.073709868
chloramphenicol	bosutinib	6.463174248	celecoxib	vinblastine	4.037981506
prazosin	capsaicin	6.44934972	clozapine	etoposide	3.962379047
chloroquine	diazoxide	6.376141892	azelastine	etoposide	3.939013998
fluoxetine	bosutinib	6.362696037	cetirizine	etoposide	3.902027951
pirfenidone	diazoxide	6.349019846	calcitriol	eplerenone	3.892257556
nifedipine	bosutinib	6.307586791	levofloxacin	chloroquine	3.879570337
diazoxide	decitabine	6.306423608	formoterol	calcitriol	3.871111131
nifedipine	calcitriol	6.29614116	palbociclib	etoposide	3.843507397
chloramphenicol	calcitriol	6.288009502	celecoxib	azelastine	3.80109154
panobinostat	calcitriol	6.278235664	olopatadine	vinblastine	3.798529102
chloroquine	decitabine	6.23034264	omeprazole	olopatadine	3.790905062
gatifloxacin	calcitriol	6.212453038	celecoxib	cetirizine	3.765400539
chloroquine	olopatadine	6.203805076	panobinostat	etoposide	3.744163188
chloroquine	fludarabine	6.198487483	celecoxib	palbociclib	3.723874522
pirfenidone	olopatadine	6.180567592	clozapine	celecoxib	3.640549803
fluoxetine	calcitriol	6.179207607	calcitriol	lamivudine	3.632897392
chloramphenicol	capsaicin	6.177032295	celecoxib	panobinostat	3.60496504
pirfenidone	fludarabine	6.173413569	latanoprost	etoposide	3.597854414
olopatadine	decitabine	6.165487869	chloroquine	prazosin	3.578940484
fludarabine	decitabine	6.142816694	calcitriol	bepridil	3.575814866
diazoxide	panobinostat	6.108635509	olopatadine	azelastine	3.512075834
fluoxetine	capsaicin	6.097821145	levofloxacin	pirfenidone	3.502988013
pirfenidone	decitabine	6.07744227	olopatadine	cetirizine	3.479098596
latanoprost	bosutinib	6.044897757	olopatadine	palbociclib	3.45017664
olopatadine	panobinostat	5.960978974	etoposide	eplerenone	3.429572581
fludarabine	panobinostat	5.945590818	etoposide	formoterol	3.384223294
latanoprost	calcitriol	5.867549374	celecoxib	eplerenone	3.359308598
chloroquine	panobinostat	5.842159773	olopatadine	panobinostat	3.325743087
chloroquine	pirfenidone	5.812756938	celecoxib	latanoprost	3.311392711
capsaicin	latanoprost	5.79785761	celecoxib	formoterol	3.265726534
nifedipine	capsaicin	5.78549348	olopatadine	clozapine	3.248015411
pirfenidone	panobinostat	5.762941578	pirfenidone	prazosin	3.23619777
decitabine	calcitriol	5.746339288	etoposide	lamivudine	3.175093927
teniposide	decitabine	5.598320595	chloroquine	fluoxetine	3.146474322
levofloxacin	decitabine	5.496661826	etoposide	bepridil	3.139635287
miglitol	bosutinib	5.456489619	olopatadine	eplerenone	3.135374132
mifepristone	decitabine	5.317404207	chloroquine	capsaicin	3.112670069
capsaicin	azacitidine	5.307006646	celecoxib	lamivudine	3.062293396
teniposide	diazoxide	5.301116974	chloroquine	vinblastine	3.062212457
miglitol	calcitriol	5.289476135	celecoxib	bepridil	3.05486555
panobinostat	decitabine	5.269077601	chloroquine	chloramphenicol	3.0255693
capsaicin	miglitol	5.244044223	olopatadine	formoterol	3.017417266
teniposide	fludarabine	5.149136873	olopatadine	latanoprost	2.95817308
teniposide	olopatadine	5.147403845	pirfenidone	fluoxetine	2.842211073
bosutinib	azacitidine	5.11375698	olopatadine	bepridil	2.838493882
prazosin	decitabine	5.108626136	olopatadine	lamivudine	2.828424195
levofloxacin	diazoxide	5.067247619	pirfenidone	capsaicin	2.814580455
bosutinib	amitriptyline	5.052563488	pirfenidone	vinblastine	2.79269724
teniposide	panobinostat	5.037139424	diazoxide	chloroquine	2.771623245
capsaicin	amitriptyline	5.02848583	pirfenidone	chloramphenicol	2.726482439
diazoxide	gatifloxacin	5.01429831	chloroquine	azelastine	2.606777545
gatifloxacin	decitabine	4.989598939	chloroquine	palbociclib	2.594778795
omeprazole	bosutinib	4.941331451	levofloxacin	prazosin	2.588860113
levofloxacin	fludarabine	4.920486476	chloroquine	cetirizine	2.582300761

levofloxacin	olopatadine	4.916692526	diazoxide	pirfenidone	2.453701006
chloramphenicol	decitabine	4.907616944	chloroquine	panobinostat	2.450082077
bosutinib	azelastine	4.891077519	chloroquine	eplerenone	2.440332241
bosutinib	clozapine	4.877963471	chloroquine	omeprazole	2.437268695
levofloxacin	panobinostat	4.877423163	levofloxacin	vinblastine	2.380805508
olopatadine	gatifloxacin	4.876526966	pirfenidone	azelastine	2.353742734
fludarabine	gatifloxacin	4.873668236	pirfenidone	palbociclib	2.346784866
capsaicin	clozapine	4.862015344	pirfenidone	cetirizine	2.331641863
bosutinib	vinblastine	4.847227862	levofloxacin	fluoxetine	2.255535594
fluoxetine	decitabine	4.83217151	levofloxacin	capsaicin	2.251579042
mifepristone	diazoxide	4.824864381	chloroquine	formoterol	2.239625778
bosutinib	irbesartan	4.814656268	pirfenidone	eplerenone	2.2163752
omeprazole	calcitriol	4.785705009	pirfenidone	panobinostat	2.210157057
amitriptyline	calcitriol	4.784519012	prazosin	vinblastine	2.199998473
nifedipine	decitabine	4.777208077	chloroquine	bepridil	2.163974165
capsaicin	omeprazole	4.755613507	pirfenidone	omeprazole	2.15769898
azelastine	calcitriol	4.733566279	levofloxacin	chloramphenicol	2.123388761
capsaicin	vinblastine	4.726039969	prazosin	fluoxetine	2.106204471
teniposide	pirfenidone	4.716703726	prazosin	capsaicin	2.099978317
capsaicin	azelastine	4.712527614	chloroquine	lamivudine	2.095655157
azacitidine	calcitriol	4.699529229	pirfenidone	formoterol	2.022229673
mifepristone	fludarabine	4.68426781	chloroquine	clozapine	1.994943661
mifepristone	panobinostat	4.680106913	prazosin	chloramphenicol	1.988484619
mifepristone	olopatadine	4.679424793	pirfenidone	bepridil	1.960447546
capsaicin	irbesartan	4.67773017	fluoxetine	vinblastine	1.931837123
vinblastine	calcitriol	4.654503522	capsaicin	vinblastine	1.913378954
bosutinib	lamivudine	4.649050374	pirfenidone	lamivudine	1.891812204
prazosin	diazoxide	4.643786676	levofloxacin	palbociclib	1.880440891
irbesartan	calcitriol	4.634099039	levofloxacin	azelastine	1.861954766
gatifloxacin	panobinostat	4.63306045	chloramphenicol	vinblastine	1.852454208
chloroquine	teniposide	4.627309891	levofloxacin	cetirizine	1.844471622
clozapine	calcitriol	4.614388583	levofloxacin	eplerenone	1.833259929
latanoprost	decitabine	4.591051152	fluoxetine	capsaicin	1.831804593
pirfenidone	gatifloxacin	4.546300444	chloroquine	latanoprost	1.83115226
bosutinib	nilotinib	4.521281092	pirfenidone	clozapine	1.753813364
chloroquine	gatifloxacin	4.508890714	prazosin	palbociclib	1.753398213
prazosin	fludarabine	4.508560939	prazosin	azelastine	1.739517994
prazosin	olopatadine	4.504035217	levofloxacin	panobinostat	1.735370458
prazosin	panobinostat	4.500498218	chloramphenicol	fluoxetine	1.729520134
capsaicin	lamivudine	4.494996165	chloramphenicol	capsaicin	1.729421482
lamivudine	calcitriol	4.489047207	prazosin	cetirizine	1.723184492
levofloxacin	pirfenidone	4.468323384	prazosin	eplerenone	1.701408459
diazoxide	chloramphenicol	4.419852376	diazoxide	vinblastine	1.662232505
diazoxide	azacitidine	4.389026184	levofloxacin	diazoxide	1.638464922
diazoxide	fluoxetine	4.386888586	prazosin	panobinostat	1.623094564
nilotinib	calcitriol	4.372980301	pirfenidone	latanoprost	1.611783317
levofloxacin	chloroquine	4.360469594	azelastine	vinblastine	1.599721051
capsaicin	nilotinib	4.360338955	levofloxacin	formoterol	1.599707616
chloramphenicol	panobinostat	4.302975953	palbociclib	vinblastine	1.595422299
chloramphenicol	fludarabine	4.290683923	levofloxacin	bepridil	1.59122525
capsaicin	fluvastatin	4.285767719	cetirizine	vinblastine	1.584700196
chloramphenicol	olopatadine	4.285709578	diazoxide	prazosin	1.573482561
olopatadine	azacitidine	4.279984318	fluoxetine	palbociclib	1.529752571
fludarabine	azacitidine	4.270668973	capsaicin	palbociclib	1.52496253
bosutinib	fluvastatin	4.266451114	fluoxetine	azelastine	1.515549153
fludarabine	fluoxetine	4.259080673	capsaicin	azelastine	1.51289065
olopatadine	fluoxetine	4.254714736	vinblastine	eplerenone	1.507793162

fluoxetine	panobinostat	4.25417602	panobinostat	vinblastine	1.501899971
bosutinib	formoterol	4.237431215	fluoxetine	cetirizine	1.501318645
mifepristone	pirfenidone	4.231390611	capsaicin	cetirizine	1.498685104
diazoxide	latanoprost	4.17756424	prazosin	formoterol	1.49451546
chloroquine	azacitidine	4.146495349	levofloxacin	lamivudine	1.493928856
miglitol	decitabine	4.144692792	fluoxetine	eplerenone	1.489381104
bosutinib	pimozide	4.132998028	prazosin	bepridil	1.48088278
pimozide	calcitriol	4.125552757	capsaicin	eplerenone	1.479746089
chloroquine	mifepristone	4.115797395	omeprazole	vinblastine	1.461709219
pirfenidone	azacitidine	4.107826121	chloramphenicol	palbociclib	1.444852686
formoterol	calcitriol	4.100969864	levofloxacin	omeprazole	1.440808836
bosutinib	icosapent	4.089788583	chloramphenicol	azelastine	1.426756486
capsaicin	formoterol	4.082742818	chloramphenicol	eplerenone	1.417872095
prazosin	pirfenidone	4.075141169	chloramphenicol	cetirizine	1.413359712
bosutinib	vemurafenib	4.069152983	fluoxetine	panobinostat	1.412972144
fludarabine	latanoprost	4.055962661	capsaicin	panobinostat	1.411635061
bosutinib	estrone	4.052775824	prazosin	lamivudine	1.39606133
olopatadine	latanoprost	4.051960201	prazosin	omeprazole	1.383665617
latanoprost	panobinostat	4.046646781	vinblastine	formoterol	1.374408227
capsaicin	estrone	4.028108795	diazoxide	capsaicin	1.368486594
calcitriol	fluvastatin	4.014069091	diazoxide	fluoxetine	1.344276097
icosapent	calcitriol	3.969678879	vinblastine	bepridil	1.333142251
chloroquine	prazosin	3.965306864	chloramphenicol	panobinostat	1.327629008
nifedipine	panobinostat	3.938118041	fluoxetine	formoterol	1.302091526
vemurafenib	calcitriol	3.935682271	capsaicin	formoterol	1.29980746
capsaicin	vemurafenib	3.924305059	fluoxetine	bepridil	1.293767141
capsaicin	icosapent	3.922833692	capsaicin	bepridil	1.287950868
azacitidine	decitabine	3.904178137	vinblastine	lamivudine	1.285723196
diazoxide	amitriptyline	3.879485828	azelastine	palbociclib	1.263513911
pirfenidone	chloramphenicol	3.86605665	cetirizine	palbociclib	1.25164993
bosutinib	mebendazole	3.863732353	azelastine	cetirizine	1.239345286
panobinostat	azacitidine	3.856080254	palbociclib	eplerenone	1.234637149
bosutinib	eplerenone	3.852525145	azelastine	eplerenone	1.231811877
pirfenidone	fluoxetine	3.847993374	chloramphenicol	bepridil	1.225923306
amitriptyline	decitabine	3.846596497	chloramphenicol	formoterol	1.225804869
calcitriol	estrone	3.841287756	cetirizine	eplerenone	1.220245568
nifedipine	diazoxide	3.797291985	fluoxetine	lamivudine	1.21608406
diazoxide	miglitol	3.7933416	capsaicin	lamivudine	1.214180101
capsaicin	pimozide	3.790817569	diazoxide	chloramphenicol	1.205927477
olopatadine	amitriptyline	3.77323782	omeprazole	capsaicin	1.203399322
fludarabine	amitriptyline	3.770823394	diazoxide	eplerenone	1.20256773
capsaicin	mebendazole	3.767133309	clozapine	vinblastine	1.186709424
diazoxide	clozapine	3.760934514	omeprazole	fluoxetine	1.182109457
chloroquine	chloramphenicol	3.754528815	palbociclib	panobinostat	1.179143086
omeprazole	decitabine	3.753720973	azelastine	panobinostat	1.166037563
chloroquine	fluoxetine	3.74328572	cetirizine	panobinostat	1.155088853
eplerenone	calcitriol	3.742450519	panobinostat	eplerenone	1.153163523
azelastine	decitabine	3.715811889	diazoxide	palbociclib	1.147435959
clozapine	decitabine	3.71403941	chloramphenicol	lamivudine	1.144322236
calcitriol	mebendazole	3.710111503	diazoxide	azelastine	1.10092438
capsaicin	eplerenone	3.690586334	levofloxacin	clozapine	1.093482926
vinblastine	decitabine	3.685315812	latanoprost	vinblastine	1.090829303
fludarabine	miglitol	3.683170559	diazoxide	cetirizine	1.090587062
nifedipine	fludarabine	3.680582761	palbociclib	formoterol	1.085554205
olopatadine	miglitol	3.679890766	palbociclib	bepridil	1.075048143
nifedipine	olopatadine	3.668060101	azelastine	formoterol	1.074882404
pirfenidone	latanoprost	3.667307803	azelastine	bepridil	1.069182897

miglitol	panobinostat	3.664096274	cetirizine	formoterol	1.064789612
irbesartan	decitabine	3.65971561	prazosin	clozapine	1.063111473
olopatadine	clozapine	3.658307552	omeprazole	chloramphenicol	1.060450512
fludarabine	clozapine	3.65574613	cetirizine	bepridil	1.059143621
panobinostat	amitriptyline	3.578600853	formoterol	eplerenone	1.058317246
chloroquine	latanoprost	3.569222656	omeprazole	eplerenone	1.057496068
teniposide	gatifloxacin	3.561357854	eplerenone	bepridil	1.03687158
lamivudine	decitabine	3.532732046	levofloxacin	latanoprost	1.017411875
pirfenidone	amitriptyline	3.521223773	palbociclib	lamivudine	1.014080226
teniposide	azacitidine	3.517012254	omeprazole	palbociclib	1.009015113
diazoxide	vinblastine	3.513333884	diazoxide	panobinostat	1.006798557
chloroquine	amitriptyline	3.494404813	azelastine	lamivudine	1.003807086
panobinostat	clozapine	3.462787561	panobinostat	formoterol	1.00180692
diazoxide	irbesartan	3.454542726	diazoxide	bepridil	1.000755452
diazoxide	omeprazole	3.449381951	panobinostat	bepridil	0.999068688
nilotinib	decitabine	3.435080533	cetirizine	lamivudine	0.994381668
diazoxide	azelastine	3.425522534	eplerenone	lamivudine	0.989360659
pirfenidone	clozapine	3.417782468	prazosin	latanoprost	0.986915042
fludarabine	vinblastine	3.412864074	omeprazole	azelastine	0.968114455
olopatadine	vinblastine	3.412083065	omeprazole	cetirizine	0.959024178
chloroquine	clozapine	3.394097212	diazoxide	formoterol	0.945864608
levofloxacin	azacitidine	3.386032339	panobinostat	lamivudine	0.935397283
diazoxide	fluvastatin	3.36016191	clozapine	capsaicin	0.924607514
fludarabine	irbesartan	3.35538534	formoterol	bepridil	0.918593756
olopatadine	irbesartan	3.354086904	fluoxetine	clozapine	0.900379861
fludarabine	omeprazole	3.349355571	omeprazole	panobinostat	0.885343493
olopatadine	omeprazole	3.346596048	omeprazole	bepridil	0.880029397
levofloxacin	gatifloxacin	3.339180088	diazoxide	lamivudine	0.87944859
pirfenidone	miglitol	3.336698833	formoterol	lamivudine	0.862425807
panobinostat	vinblastine	3.327632587	lamivudine	bepridil	0.858369717
fludarabine	azelastine	3.326310172	capsaicin	latanoprost	0.858338082
omeprazole	panobinostat	3.325337925	clozapine	eplerenone	0.838624859
olopatadine	azelastine	3.323745415	fluoxetine	latanoprost	0.837185685
panobinostat	azelastine	3.297198836	omeprazole	formoterol	0.831760306
diazoxide	lamivudine	3.289302683	chloramphenicol	clozapine	0.789746113
panobinostat	irbesartan	3.287463127	clozapine	palbociclib	0.776593866
gatifloxacin	azacitidine	3.27583749	latanoprost	eplerenone	0.774074877
olopatadine	fluvastatin	3.270170124	omeprazole	lamivudine	0.77335638
fludarabine	fluvastatin	3.266879396	chloramphenicol	latanoprost	0.737400446
chloroquine	miglitol	3.251351204	clozapine	azelastine	0.734737739
decitabine	fluvastatin	3.250119727	clozapine	cetirizine	0.727838793
mifepristone	azacitidine	3.237988323	latanoprost	palbociclib	0.720705493
formoterol	decitabine	3.219228733	clozapine	bepridil	0.686095577
levofloxacin	teniposide	3.206857453	latanoprost	azelastine	0.683623752
fludarabine	lamivudine	3.194396731	latanoprost	cetirizine	0.67720475
olopatadine	lamivudine	3.192453391	clozapine	panobinostat	0.666059578
diazoxide	nilotinib	3.17526356	latanoprost	bepridil	0.635229629
nifedipine	pirfenidone	3.165992823	clozapine	formoterol	0.63125355
panobinostat	lamivudine	3.150891395	latanoprost	panobinostat	0.620732605
mifepristone	gatifloxacin	3.141998492	latanoprost	formoterol	0.587338716
pirfenidone	vinblastine	3.13294116	clozapine	lamivudine	0.585751789
pimozide	decitabine	3.130224891	latanoprost	lamivudine	0.545205084
prazosin	azacitidine	3.114932929	diazoxide	omeprazole	0.457298835
icosapent	decitabine	3.106170817	diazoxide	latanoprost	0.219496706
diazoxide	estrone	3.100443993	diazoxide	clozapine	0.210661556
vemurafenib	decitabine	3.09157248	omeprazole	latanoprost	0.193017738
decitabine	estrone	3.085171863	omeprazole	clozapine	0.185248416

fludarabine	nilotinib	3.083394105	clozapine	latanoprost	0.030355402
olopatadine	nilotinib	3.081152985			
chloroquine	vinblastine	3.077547813			
pirfenidone	fluvastatin	3.072471208			
pirfenidone	irbesartan	3.070514571			
panobinostat	fluvastatin	3.064475307			
chloroquine	fluvastatin	3.061815428			
panobinostat	nilotinib	3.052329837			
pirfenidone	omeprazole	3.038346702			
prazosin	gatifloxacin	3.028204655			
pirfenidone	azelastine	3.020642287			
olopatadine	estrone	3.015257131			
fludarabine	estrone	3.01348952			
chloroquine	irbesartan	3.010487575			
nifedipine	chloroquine	2.983709465			
teniposide	mifepristone	2.981262454			
chloramphenicol	azacitidine	2.972267771			
diazoxide	formoterol	2.967733808			
chloroquine	omeprazole	2.963074093			
chloroquine	azelastine	2.947731782			
fluoxetine	azacitidine	2.943637036			
decitabine	mebendazole	2.937570575			
eplerenone	decitabine	2.925734699			
pirfenidone	lamivudine	2.910315124			
fludarabine	formoterol	2.881780241			
olopatadine	formoterol	2.87955824			
teniposide	prazosin	2.87732777			
panobinostat	estrone	2.864716767			
chloramphenicol	gatifloxacin	2.861878283			
fluoxetine	gatifloxacin	2.857924586			
diazoxide	vemurafenib	2.857737204			
panobinostat	formoterol	2.85655936			
chloroquine	lamivudine	2.845748557			
diazoxide	icosapent	2.82680185			
pirfenidone	estrone	2.811071267			
pirfenidone	nilotinib	2.802531532			
latanoprost	azacitidine	2.801423508			
diazoxide	mebendazole	2.800483531			
chloroquine	estrone	2.78794006			
fludarabine	vemurafenib	2.775054695			
olopatadine	vemurafenib	2.773037687			
teniposide	amitriptyline	2.76725947			
panobinostat	vemurafenib	2.747096853			
fludarabine	icosapent	2.744522916			
olopatadine	icosapent	2.74182081			
panobinostat	icosapent	2.738033291			
chloroquine	nilotinib	2.736375957			
latanoprost	gatifloxacin	2.726278934			
fludarabine	mebendazole	2.7203989			
olopatadine	mebendazole	2.719776356			
gatifloxacin	amitriptyline	2.718388577			
teniposide	fluoxetine	2.712836866			
teniposide	chloramphenicol	2.699448984			
levofloxacin	mifepristone	2.696710423			
teniposide	clozapine	2.695649885			
diazoxide	eplerenone	2.652883542			
panobinostat	mebendazole	2.652460758			

nifedipine	azacitidine	2.646928083
gatifloxacin	clozapine	2.64203907
pirfenidone	formoterol	2.616961982
levofloxacin	prazosin	2.604718109
levofloxacin	amitriptyline	2.597252674
teniposide	latanoprost	2.592494722
panobinostat	pimozide	2.580332649
fludarabine	eplerenone	2.575557694
panobinostat	eplerenone	2.574166041
olopatadine	eplerenone	2.572864897
chloroquine	formoterol	2.553795276
miglitol	azacitidine	2.539757496
levofloxacin	clozapine	2.532895712
amitriptyline	azacitidine	2.532172495
pirfenidone	vemurafenib	2.522278379
pirfenidone	mebendazole	2.497271939
diazoxide	pimozide	2.487970535
gatifloxacin	miglitol	2.486327602
pirfenidone	icosapent	2.481647421
teniposide	fluvastatin	2.467201962
chloroquine	vemurafenib	2.462738361
levofloxacin	fluoxetine	2.45446404
chloroquine	mebendazole	2.453117822
clozapine	azacitidine	2.452295429
mifepristone	amitriptyline	2.445426572
levofloxacin	chloramphenicol	2.433770537
chloroquine	icosapent	2.41534195
fludarabine	pimozide	2.411500896
olopatadine	pimozide	2.403292904
mifepristone	prazosin	2.396259374
gatifloxacin	fluvastatin	2.391006641
mifepristone	clozapine	2.38650471
teniposide	miglitol	2.374858143
gatifloxacin	vinblastine	2.371490903
prazosin	amitriptyline	2.356688407
levofloxacin	latanoprost	2.347896286
teniposide	vinblastine	2.331964147
levofloxacin	fluvastatin	2.331131444
vinblastine	azacitidine	2.32675401
pirfenidone	eplerenone	2.326006101
gatifloxacin	irbesartan	2.315658866
omeprazole	azacitidine	2.306944667
prazosin	clozapine	2.299718313
irbesartan	azacitidine	2.293820228
azelastine	azacitidine	2.289000055
gatifloxacin	omeprazole	2.267665197
chloroquine	eplerenone	2.262135062
teniposide	irbesartan	2.261815708
gatifloxacin	azelastine	2.257327349
mifepristone	fluoxetine	2.25721102
mifepristone	chloramphenicol	2.232961274
chloramphenicol	amitriptyline	2.228091122
fluoxetine	amitriptyline	2.224283465
nifedipine	gatifloxacin	2.207669771
mifepristone	fluvastatin	2.203951562
teniposide	estrone	2.202060963
lamivudine	azacitidine	2.19209907

gatifloxacin	lamivudine	2.18337337
prazosin	fluoxetine	2.181292393
azacitidine	fluvastatin	2.179635494
chloramphenicol	clozapine	2.175145414
teniposide	omeprazole	2.172595797
fluoxetine	clozapine	2.170638483
teniposide	azelastine	2.167875599
gatifloxacin	estrone	2.167576949
mifepristone	latanoprost	2.160614428
prazosin	chloramphenicol	2.158453465
levofloxacin	miglitol	2.156060256
levofloxacin	vinblastine	2.150347504
prazosin	fluvastatin	2.122963422
latanoprost	amitriptyline	2.121628545
nilotinib	azacitidine	2.120230652
teniposide	lamivudine	2.112144101
gatifloxacin	nilotinib	2.096561232
prazosin	latanoprost	2.087784276
levofloxacin	irbesartan	2.078294384
pirfenidone	pimozide	2.07428561
latanoprost	clozapine	2.070246141
levofloxacin	estrone	2.064683871
amitriptyline	clozapine	2.048261258
chloramphenicol	fluoxetine	2.032833692
azacitidine	estrone	2.025517837
teniposide	nilotinib	2.017491786
chloramphenicol	fluvastatin	2.01209491
fluoxetine	fluvastatin	2.004365548
mifepristone	vinblastine	2.002158051
mifepristone	miglitol	1.98728079
formoterol	azacitidine	1.983096822
levofloxacin	omeprazole	1.975714347
levofloxacin	azelastine	1.973993169
fluoxetine	latanoprost	1.966767381
gatifloxacin	formoterol	1.955656874
chloroquine	pimozide	1.95482079
chloramphenicol	latanoprost	1.946475927
mifepristone	estrone	1.942762426
miglitol	amitriptyline	1.934445838
prazosin	vinblastine	1.932009789
levofloxacin	lamivudine	1.930812284
mifepristone	irbesartan	1.930673172
prazosin	miglitol	1.919928641
latanoprost	fluvastatin	1.910701697
vemurafenib	azacitidine	1.908207587
icosapent	azacitidine	1.895548513
gatifloxacin	mebendazole	1.890318836
miglitol	clozapine	1.887110203
gatifloxacin	vemurafenib	1.886905109
teniposide	formoterol	1.878159505
prazosin	estrone	1.872401351
prazosin	irbesartan	1.863524773
teniposide	mebendazole	1.858812001
azacitidine	mebendazole	1.854658993
amitriptyline	fluvastatin	1.852466766
gatifloxacin	icosapent	1.844960253
vinblastine	amitriptyline	1.842252266

levofloxacin	nilotinib	1.839043726
nifedipine	teniposide	1.835220407
mifepristone	omeprazole	1.823041973
mifepristone	azelastine	1.82300985
fluoxetine	vinblastine	1.821792529
teniposide	vemurafenib	1.815742608
chloramphenicol	vinblastine	1.814277567
fluoxetine	miglitol	1.808884175
irbesartan	amitriptyline	1.799529931
clozapine	fluvastatin	1.798670296
vinblastine	clozapine	1.794088823
chloramphenicol	miglitol	1.791768819
mifepristone	lamivudine	1.787704628
eplerenone	azacitidine	1.780700769
chloramphenicol	estrone	1.769558328
fluoxetine	estrone	1.767113675
omeprazole	amitriptyline	1.764038035
prazosin	omeprazole	1.761029788
prazosin	azelastine	1.7608219
fluoxetine	irbesartan	1.756884285
azelastine	amitriptyline	1.755775172
teniposide	icosapent	1.754608789
irbesartan	clozapine	1.753188426
chloramphenicol	irbesartan	1.747535234
latanoprost	vinblastine	1.740579596
miglitol	fluvastatin	1.73948504
pimozide	azacitidine	1.734292851
latanoprost	miglitol	1.730848793
nifedipine	amitriptyline	1.729296238
gatifloxacin	eplerenone	1.726672381
prazosin	lamivudine	1.726201731
omeprazole	clozapine	1.720567695
levofloxacin	mebendazole	1.714045112
azelastine	clozapine	1.712269064
levofloxacin	formoterol	1.710187631
nifedipine	clozapine	1.699606352
mifepristone	nilotinib	1.699581814
lamivudine	amitriptyline	1.697601172
latanoprost	estrone	1.685714456
amitriptyline	estrone	1.680835685
latanoprost	irbesartan	1.679131414
fluoxetine	omeprazole	1.659326391
fluoxetine	azelastine	1.659248276
levofloxacin	vemurafenib	1.655139353
lamivudine	clozapine	1.654830348
chloramphenicol	azelastine	1.645259211
chloramphenicol	omeprazole	1.644586347
prazosin	nilotinib	1.641468107
vinblastine	fluvastatin	1.639828592
teniposide	eplerenone	1.637448856
clozapine	estrone	1.633627211
amitriptyline	nilotinib	1.630558462
fluoxetine	lamivudine	1.626971019
nifedipine	fluvastatin	1.623608458
chloramphenicol	lamivudine	1.615457196
irbesartan	fluvastatin	1.605630941
mifepristone	mebendazole	1.595923084

miglitol	vinblastine	1.593554463
nilotinib	clozapine	1.589969821
levofloxacin	icosapent	1.589156022
latanoprost	omeprazole	1.587484116
latanoprost	azelastine	1.587207146
omeprazole	fluvastatin	1.584596865
mifepristone	formoterol	1.579381805
azelastine	fluvastatin	1.575873905
latanoprost	lamivudine	1.555737457
fluoxetine	nilotinib	1.546870172
prazosin	mebendazole	1.540007803
miglitol	irbesartan	1.538587155
miglitol	estrone	1.537347601
chloramphenicol	nilotinib	1.534406515
nifedipine	levofloxacin	1.530090117
mifepristone	vemurafenib	1.529623633
prazosin	formoterol	1.525504687
amitriptyline	formoterol	1.521132407
lamivudine	fluvastatin	1.519823691
irbesartan	vinblastine	1.513426367
clozapine	formoterol	1.483440479
levofloxacin	eplerenone	1.480713
latanoprost	nilotinib	1.479552482
fluvastatin	estrone	1.478408687
prazosin	vemurafenib	1.477321296
amitriptyline	mebendazole	1.468461952
amitriptyline	vemurafenib	1.467502616
vinblastine	estrone	1.466341703
nilotinib	fluvastatin	1.462480682
mifepristone	icosapent	1.462451752
miglitol	omeprazole	1.458238031
miglitol	azelastine	1.4575236
omeprazole	vinblastine	1.457269961
azelastine	vinblastine	1.453661708
fluoxetine	mebendazole	1.452153465
gatifloxacin	pimozide	1.446359149
chloramphenicol	mebendazole	1.446163278
fluoxetine	formoterol	1.437505419
amitriptyline	icosapent	1.435766105
irbesartan	estrone	1.431819604
clozapine	vemurafenib	1.430972839
clozapine	mebendazole	1.430070801
miglitol	lamivudine	1.427274218
chloramphenicol	formoterol	1.42538586
vinblastine	lamivudine	1.414989975
prazosin	icosapent	1.413148931
omeprazole	irbesartan	1.407808106
azelastine	irbesartan	1.404952902
omeprazole	estrone	1.40214413
clozapine	icosapent	1.400985657
azelastine	estrone	1.395752025
fluoxetine	vemurafenib	1.392183155
latanoprost	mebendazole	1.387418518
chloramphenicol	vemurafenib	1.380965863
latanoprost	formoterol	1.375091906
irbesartan	lamivudine	1.369433092
formoterol	fluvastatin	1.365273245

nifedipine	estrone	1.365047721
mifepristone	eplerenone	1.361238692
miglitol	nilotinib	1.358310516
vinblastine	nilotinib	1.352481008
lamivudine	estrone	1.350024755
amitriptyline	eplerenone	1.343912144
omeprazole	azelastine	1.335607518
latanoprost	vemurafenib	1.331597234
fluoxetine	icosapent	1.331241035
nifedipine	mifepristone	1.32784258
chloramphenicol	icosapent	1.317533506
vemurafenib	fluvastatin	1.316232614
prazosin	eplerenone	1.315509409
clozapine	eplerenone	1.311572115
irbesartan	nilotinib	1.307649454
mebendazole	fluvastatin	1.307109747
omeprazole	lamivudine	1.307048649
azelastine	lamivudine	1.305253164
nilotinib	estrone	1.296347021
icosapent	fluvastatin	1.292979642
nifedipine	prazosin	1.292223479
latanoprost	icosapent	1.274110826
miglitol	mebendazole	1.270224572
miglitol	formoterol	1.26273934
vinblastine	formoterol	1.259393554
nifedipine	vinblastine	1.254848166
omeprazole	nilotinib	1.244472927
azelastine	nilotinib	1.243216475
vinblastine	mebendazole	1.242727192
fluoxetine	eplerenone	1.239153383
chloramphenicol	eplerenone	1.225709424
miglitol	vemurafenib	1.222479464
vinblastine	vemurafenib	1.217232907
irbesartan	formoterol	1.217194219
lamivudine	nilotinib	1.215514872
eplerenone	fluvastatin	1.211430384
nifedipine	fluoxetine	1.211236458
formoterol	estrone	1.209222953
irbesartan	mebendazole	1.206354351
teniposide	pimozide	1.2022416
latanoprost	eplerenone	1.186159658
nifedipine	irbesartan	1.178212137
vinblastine	icosapent	1.178014021
irbesartan	vemurafenib	1.176884509
miglitol	icosapent	1.171532073
nifedipine	latanoprost	1.169713401
mebendazole	estrone	1.168823097
vemurafenib	estrone	1.166712319
omeprazole	mebendazole	1.161591998
nifedipine	chloramphenicol	1.15996937
azelastine	mebendazole	1.158715854
omeprazole	formoterol	1.157116191
azelastine	formoterol	1.156108149
icosapent	estrone	1.14077695
irbesartan	icosapent	1.136448854
pimozide	amitriptyline	1.132956342
lamivudine	formoterol	1.130818409

lamivudine	mebendazole	1.12789056
omeprazole	vemurafenib	1.120025635
azelastine	vemurafenib	1.118894828
pimozide	clozapine	1.113509766
vinblastine	eplerenone	1.099755693
nifedipine	miglitol	1.099316228
lamivudine	vemurafenib	1.093963385
miglitol	eplerenone	1.091081457
nilotinib	mebendazole	1.078064572
nilotinib	formoterol	1.077072338
omeprazole	icosapent	1.074484796
azelastine	icosapent	1.074289273
eplerenone	estrone	1.067637121
pimozide	fluvastatin	1.063741074
irbesartan	eplerenone	1.06038073
lamivudine	icosapent	1.052965589
nifedipine	lamivudine	1.047704373
nilotinib	vemurafenib	1.0422835
nifedipine	azelastine	1.034359985
nifedipine	omeprazole	1.023011189
formoterol	mebendazole	1.003864427
levofloxacin	pimozide	1.002291676
nilotinib	icosapent	1.001417813
azelastine	eplerenone	1.000980439
omeprazole	eplerenone	1.000959019
nifedipine	mebendazole	1.000241292
lamivudine	eplerenone	0.981709688
nifedipine	nilotinib	0.973083714
vemurafenib	mebendazole	0.970258114
formoterol	vemurafenib	0.969365104
icosapent	mebendazole	0.938996683
nilotinib	eplerenone	0.93323851
formoterol	icosapent	0.930720661
icosapent	vemurafenib	0.901276032
nifedipine	formoterol	0.896127545
pimozide	estrone	0.894313672
eplerenone	mebendazole	0.876616857
nifedipine	vemurafenib	0.875775342
mifepristone	pimozide	0.869768385
formoterol	eplerenone	0.86720886
prazosin	pimozide	0.84644189
vemurafenib	eplerenone	0.839914659
pimozide	vinblastine	0.822053796
icosapent	eplerenone	0.802875467
fluoxetine	pimozide	0.793389952
nifedipine	icosapent	0.792152673
pimozide	irbesartan	0.771834702
latanoprost	pimozide	0.766196866
chloramphenicol	pimozide	0.759788157
nifedipine	eplerenone	0.726956278
miglitol	pimozide	0.720097137
pimozide	lamivudine	0.686319939
azelastine	pimozide	0.677561663
omeprazole	pimozide	0.670121804
pimozide	mebendazole	0.655260272
pimozide	nilotinib	0.63742685
pimozide	formoterol	0.587011947

pimozide	vemurafenib	0.573684165
pimozide	icosapent	0.518883644
pimozide	eplerenone	0.476172531
nifedipine	pimozide	0.085782058

NF1_PrimaryMelanoma_TCGASKCM			TWT_PrimaryMelanoma_TCGASKCM		
drug1	drug2	score	drug1	drug2	score
mifepristone	tretinoin	27.55449655	chloroquine	tretinoin	9.909066141
tretinoin	estradiol	26.41790275	pirfenidone	tretinoin	9.609671428
chloroquine	tretinoin	25.86051977	tretinoin	bosutinib	9.266556553
pirfenidone	tretinoin	24.01822385	tretinoin	estradiol	9.071217195
fluoxetine	tretinoin	23.27403058	tretinoin	etoposide	8.536740237
levofloxacin	tretinoin	22.86953569	nifedipine	tretinoin	8.111411285
prazosin	tretinoin	22.61938055	mifepristone	tretinoin	7.911821032
tretinoin	bosutinib	22.4815161	tretinoin	fluoxetine	7.639154805
nifedipine	tretinoin	22.11713947	prazosin	tretinoin	7.411372908
tretinoin	capsaicin	21.23007606	latanoprost	tretinoin	7.034566603
tretinoin	olopatadine	19.73041145	tretinoin	olopatadine	6.956407434
tretinoin	calcitriol	19.61787809	tretinoin	rofecoxib	6.770685356
tretinoin	propranolol	19.17318406	tretinoin	capsaicin	6.271161691
tretinoin	latanoprost	19.08541245	chloroquine	estradiol	6.134989961
tretinoin	etoposide	18.88252601	pirfenidone	estradiol	5.909737
tretinoin	celecoxib	18.68422389	tretinoin	vinblastine	5.884420996
mifepristone	estradiol	17.64193635	bosutinib	estradiol	5.821879987
tretinoin	rofecoxib	16.97597326	tretinoin	testosterone	5.669149928
tretinoin	alitretinoin	16.96115661	tretinoin	icosapent	5.637516259
chloroquine	estradiol	16.30624986	etoposide	estradiol	5.490484934
tretinoin	azelastine	16.22056525	chloroquine	etoposide	5.40895997
tretinoin	pimozide	15.46723421	bosutinib	etoposide	5.222156473
pirfenidone	estradiol	14.99678853	tretinoin	decitabine	5.200477083
tretinoin	vinblastine	14.85609911	pirfenidone	etoposide	5.168324334
tretinoin	flurbiprofen	14.81656745	tretinoin	fluvastatin	5.101344656
bosutinib	estradiol	14.58860748	chloroquine	bosutinib	4.986632108
fluoxetine	estradiol	14.58137438	tretinoin	propranolol	4.942778621
tretinoin	eplerenone	14.36108478	tretinoin	clozapine	4.864861012
tretinoin	azacitidine	14.23908546	tretinoin	eplerenone	4.848036143
levofloxacin	estradiol	14.1764279	nifedipine	estradiol	4.823971953
prazosin	estradiol	14.05748389	tretinoin	lamivudine	4.785259251
tretinoin	icosapent	13.90273672	mifepristone	estradiol	4.699301118
tretinoin	formoterol	13.66326893	tretinoin	azacitidine	4.659993668
nifedipine	estradiol	13.60506686	pirfenidone	bosutinib	4.655881926
capsaicin	estradiol	13.52768942	fluoxetine	estradiol	4.633517443
estradiol	calcitriol	13.00141017	tretinoin	paclitaxel	4.596231487
tretinoin	fluvastatin	12.88796868	tretinoin	pimozide	4.560807199
tretinoin	clozapine	12.66773481	prazosin	estradiol	4.386563204
tretinoin	cladribine	12.66072811	pirfenidone	chloroquine	4.352450997
tretinoin	paclitaxel	12.64318132	olopatadine	estradiol	4.291418959
mifepristone	calcitriol	12.62232841	latanoprost	estradiol	4.16927391
tretinoin	naproxen	12.57748518	rofecoxib	estradiol	4.116192531
tretinoin	cetirizine	12.56728479	nifedipine	etoposide	4.04437592
olopatadine	estradiol	12.4281673	fluoxetine	etoposide	3.983889246
mifepristone	bosutinib	12.37816977	mifepristone	etoposide	3.933300978
estradiol	etoposide	12.26090612	olopatadine	etoposide	3.76723191
estradiol	celecoxib	12.22706751	capsaicin	estradiol	3.711707327
propranolol	estradiol	12.10148767	prazosin	etoposide	3.654521129
tretinoin	lamivudine	11.87489166	vinblastine	estradiol	3.589234294

latanoprost	estradiol	11.72975224	rofecoxib	etoposide	3.549253788
chloroquine	calcitriol	11.51876897	estradiol	testosterone	3.545679827
chloroquine	mifepristone	11.04750853	latanoprost	etoposide	3.479811582
mifepristone	celecoxib	10.97650267	bosutinib	olopatadine	3.430786806
estradiol	alitretinoin	10.7360167	estradiol	icosapent	3.427290015
chloroquine	bosutinib	10.62653674	fluoxetine	bosutinib	3.410449351
bosutinib	calcitriol	10.55242706	chloroquine	olopatadine	3.236517085
rofecoxib	estradiol	10.52578494	nifedipine	bosutinib	3.18791993
pirfenidone	calcitriol	10.50535693	etoposide	testosterone	3.163747304
mifepristone	etoposide	10.44858111	estradiol	decitabine	3.137927159
fluoxetine	calcitriol	10.24410613	etoposide	vinblastine	3.10758368
azelastine	estradiol	10.05484662	estradiol	fluvastatin	3.095189163
mifepristone	capsaicin	10.01756102	capsaicin	etoposide	3.092287109
levofloxacin	calcitriol	9.868398224	mifepristone	bosutinib	3.082517951
prazosin	calcitriol	9.807576789	bosutinib	rofecoxib	3.065362336
chloroquine	celecoxib	9.6815557	chloroquine	fluoxetine	3.04708615
capsaicin	calcitriol	9.640409849	bosutinib	testosterone	3.014313066
pimozide	estradiol	9.514378978	estradiol	propranolol	2.955460094
bosutinib	celecoxib	9.436431681	etoposide	icosapent	2.955236418
nifedipine	calcitriol	9.406806571	pirfenidone	olopatadine	2.948375859
mifepristone	fluoxetine	9.311295845	chloroquine	testosterone	2.947750352
pirfenidone	bosutinib	9.286377434	estradiol	eplerenone	2.944564739
capsaicin	bosutinib	9.280656077	estradiol	clozapine	2.892952571
pirfenidone	mifepristone	9.267582559	estradiol	lamivudine	2.845165062
estradiol	vinblastine	9.266861081	prazosin	bosutinib	2.817598565
estradiol	flurbiprofen	9.206511526	estradiol	paclitaxel	2.782277076
fluoxetine	bosutinib	9.192958744	chloroquine	rofecoxib	2.761222961
bosutinib	etoposide	9.158119771	estradiol	azacitidine	2.75810663
chloroquine	etoposide	8.989494798	pirfenidone	testosterone	2.737305942
estradiol	eplerenone	8.930213027	bosutinib	vinblastine	2.717568521
celecoxib	calcitriol	8.903864911	estradiol	pimozide	2.712143035
etoposide	calcitriol	8.87322101	latanoprost	bosutinib	2.700219274
estradiol	azacitidine	8.849301334	pirfenidone	fluoxetine	2.690807349
olopatadine	calcitriol	8.771647482	etoposide	decitabine	2.680325744
pirfenidone	celecoxib	8.626812228	etoposide	fluvastatin	2.662288102
estradiol	icosapent	8.620254905	nifedipine	chloroquine	2.620004209
propranolol	calcitriol	8.5556404	bosutinib	icosapent	2.552330982
prazosin	bosutinib	8.485631871	etoposide	eplerenone	2.536029776
fluoxetine	celecoxib	8.481153989	chloroquine	mifepristone	2.51724864
estradiol	formoterol	8.469623056	etoposide	propranolol	2.495298136
levofloxacin	bosutinib	8.435391265	chloroquine	vinblastine	2.475700121
mifepristone	olopatadine	8.342991245	pirfenidone	rofecoxib	2.450207569
capsaicin	celecoxib	8.296611083	etoposide	clozapine	2.425152247
mifepristone	prazosin	8.285295283	etoposide	paclitaxel	2.386215115
mifepristone	propranolol	8.270719754	etoposide	lamivudine	2.38459695
levofloxacin	mifepristone	8.131586706	bosutinib	capsaicin	2.384121863
latanoprost	calcitriol	8.103809126	chloroquine	icosapent	2.299093595
estradiol	fluvastatin	8.071033	etoposide	azacitidine	2.297825994
olopatadine	bosutinib	8.057202326	bosutinib	fluvastatin	2.281869613
chloroquine	capsaicin	7.973318207	etoposide	pimozide	2.273580232
prazosin	celecoxib	7.961684959	chloroquine	prazosin	2.258697226
levofloxacin	celecoxib	7.95953945	bosutinib	decitabine	2.247614907
etoposide	celecoxib	7.944899093	pirfenidone	vinblastine	2.211362729
propranolol	bosutinib	7.925586016	nifedipine	pirfenidone	2.193324491
estradiol	cladribine	7.921408065	bosutinib	eplerenone	2.182413381
pirfenidone	etoposide	7.868332508	chloroquine	latanoprost	2.180604169
capsaicin	etoposide	7.838972866	pirfenidone	mifepristone	2.0980584

estradiol	naproxen	7.81345348	chloroquine	fluvastatin	2.041071977
estradiol	paclitaxel	7.806020439	pirfenidone	icosapent	2.040130988
estradiol	clozapine	7.792319434	fluoxetine	olopatadine	2.036498431
estradiol	cetirizine	7.790241528	bosutinib	paclitaxel	2.026855631
fluoxetine	etoposide	7.784737043	olopatadine	testosterone	2.022244163
nifedipine	bosutinib	7.743773689	bosutinib	propranolol	2.014463121
alitretinoin	calcitriol	7.608602013	fluoxetine	testosterone	1.980054874
mifepristone	alitretinoin	7.522669316	chloroquine	decitabine	1.969113287
nifedipine	celecoxib	7.438453734	chloroquine	eplerenone	1.959392269
olopatadine	celecoxib	7.355061919	chloroquine	capsaicin	1.911205345
rofecoxib	calcitriol	7.328764429	bosutinib	clozapine	1.91084449
estradiol	lamivudine	7.309918442	bosutinib	lamivudine	1.877540712
propranolol	celecoxib	7.207394607	pirfenidone	prazosin	1.858214848
prazosin	etoposide	7.195803254	rofecoxib	olopatadine	1.850042529
nifedipine	mifepristone	7.158968796	nifedipine	testosterone	1.810363934
levofloxacin	etoposide	7.156593916	pirfenidone	fluvastatin	1.803644979
bosutinib	alitretinoin	7.132274855	pirfenidone	latanoprost	1.803371005
azelastine	calcitriol	6.9993112	chloroquine	paclitaxel	1.797684954
olopatadine	etoposide	6.817055473	bosutinib	pimozide	1.79141671
propranolol	etoposide	6.703620884	rofecoxib	testosterone	1.783686473
chloroquine	fluoxetine	6.680325354	bosutinib	azacitidine	1.771600436
latanoprost	bosutinib	6.641307827	mifepristone	testosterone	1.747646427
fluoxetine	capsaicin	6.64128882	pirfenidone	eplerenone	1.735300873
nifedipine	etoposide	6.579747563	pirfenidone	decitabine	1.718298814
pimozide	calcitriol	6.578372879	nifedipine	olopatadine	1.70431759
pirfenidone	capsaicin	6.564511159	chloroquine	propranolol	1.698594789
vinblastine	calcitriol	6.485963679	olopatadine	vinblastine	1.664379038
chloroquine	pirfenidone	6.472799199	mifepristone	olopatadine	1.633883887
alitretinoin	celecoxib	6.451660143	prazosin	testosterone	1.589960718
flurbiprofen	calcitriol	6.422145793	vinblastine	testosterone	1.58623595
latanoprost	celecoxib	6.393167199	pirfenidone	paclitaxel	1.580521591
rofecoxib	bosutinib	6.27210967	fluoxetine	rofecoxib	1.58037208
chloroquine	propranolol	6.243364855	pirfenidone	capsaicin	1.572335641
eplerenone	calcitriol	6.233489931	chloroquine	clozapine	1.56976054
chloroquine	olopatadine	6.217484348	chloroquine	lamivudine	1.541181715
azacitidine	calcitriol	6.173949979	olopatadine	icosapent	1.540411981
mifepristone	latanoprost	6.107867324	latanoprost	testosterone	1.526562908
mifepristone	rofecoxib	6.054009931	icosapent	testosterone	1.48516154
alitretinoin	etoposide	6.030033604	chloroquine	pimozide	1.471650506
olopatadine	capsaicin	6.010661099	prazosin	olopatadine	1.456606857
icosapent	calcitriol	6.002005351	fluoxetine	vinblastine	1.451931813
bosutinib	azelastine	5.982929268	pirfenidone	propranolol	1.446615299
capsaicin	propranolol	5.979233674	chloroquine	azacitidine	1.420184209
rofecoxib	celecoxib	5.914941023	latanoprost	olopatadine	1.409897984
formoterol	calcitriol	5.895816191	olopatadine	fluvastatin	1.364611505
prazosin	capsaicin	5.807450231	capsaicin	testosterone	1.345351377
chloroquine	alitretinoin	5.777323797	rofecoxib	vinblastine	1.331740024
mifepristone	azelastine	5.767451274	fluvastatin	testosterone	1.325230629
bosutinib	vinblastine	5.707769768	fluoxetine	icosapent	1.315874661
mifepristone	vinblastine	5.67072375	pirfenidone	clozapine	1.313731356
fluvastatin	calcitriol	5.668244145	olopatadine	eplerenone	1.311491789
levofloxacin	capsaicin	5.663924565	olopatadine	decitabine	1.308049991
azelastine	celecoxib	5.645424147	decitabine	testosterone	1.298006714
latanoprost	etoposide	5.64403632	pirfenidone	lamivudine	1.289114404
cladribine	calcitriol	5.558757198	eplerenone	testosterone	1.268761602
bosutinib	flurbiprofen	5.551776404	capsaicin	olopatadine	1.232513494
chloroquine	prazosin	5.550003119	pirfenidone	pimozide	1.231623147

capsaicin	alitretinoin	5.46397156	rofecoxib	icosapent	1.211141569
naproxen	calcitriol	5.449323082	olopatadine	paclitaxel	1.198762547
cetirizine	calcitriol	5.422889759	nifedipine	fluoxetine	1.182552732
mifepristone	flurbiprofen	5.415882099	testosterone	paclitaxel	1.174416015
bosutinib	pimozide	5.415132062	pirfenidone	azacitidine	1.168375891
paclitaxel	calcitriol	5.414850258	propranolol	testosterone	1.151606671
bosutinib	eplerenone	5.407629842	fluoxetine	fluvastatin	1.150062172
clozapine	calcitriol	5.387717154	nifedipine	rofecoxib	1.130388645
bosutinib	azacitidine	5.341774817	olopatadine	propranolol	1.114511926
rofecoxib	etoposide	5.321010982	mifepristone	fluoxetine	1.11390011
vinblastine	celecoxib	5.31333565	fluoxetine	eplerenone	1.113276766
chloroquine	levofloxacin	5.308786556	vinblastine	icosapent	1.108854664
mifepristone	eplerenone	5.294551458	nifedipine	vinblastine	1.085577269
mifepristone	azacitidine	5.215661293	clozapine	testosterone	1.085015517
flurbiprofen	celecoxib	5.211054395	mifepristone	rofecoxib	1.068502788
pimozide	celecoxib	5.201739238	lamivudine	testosterone	1.065888587
bosutinib	icosapent	5.13664154	rofecoxib	fluvastatin	1.060926813
mifepristone	fluvastatin	5.133368067	fluoxetine	decitabine	1.057094667
bosutinib	fluvastatin	5.077228772	mifepristone	vinblastine	1.030490568
azelastine	etoposide	5.075922842	rofecoxib	eplerenone	1.025750442
eplerenone	celecoxib	5.067462231	olopatadine	clozapine	1.020982018
lamivudine	calcitriol	5.057428849	pimozide	testosterone	1.017202047
fluoxetine	propranolol	5.050489991	olopatadine	lamivudine	1.002122516
bosutinib	formoterol	5.039674653	azacitidine	testosterone	0.999707743
azacitidine	celecoxib	5.011945942	fluoxetine	paclitaxel	0.993532631
mifepristone	pimozide	5.005919744	rofecoxib	decitabine	0.982212747
mifepristone	cladribine	4.993707768	vinblastine	fluvastatin	0.974224278
pirfenidone	fluoxetine	4.992708645	olopatadine	pimozide	0.957170642
fluoxetine	olopatadine	4.9921282	nifedipine	icosapent	0.941202261
bosutinib	cladribine	4.958842274	prazosin	fluoxetine	0.94082773
mifepristone	icosapent	4.958025374	vinblastine	eplerenone	0.940426548
pirfenidone	propranolol	4.904526459	latanoprost	fluoxetine	0.930953327
nifedipine	capsaicin	4.880512782	rofecoxib	paclitaxel	0.919136117
mifepristone	formoterol	4.858168416	olopatadine	azacitidine	0.91585983
icosapent	celecoxib	4.844132734	prazosin	rofecoxib	0.912526762
vinblastine	etoposide	4.837010985	vinblastine	decitabine	0.910432815
pirfenidone	olopatadine	4.825329975	latanoprost	rofecoxib	0.898829232
formoterol	celecoxib	4.755379799	prazosin	vinblastine	0.891697155
fluoxetine	alitretinoin	4.719389856	mifepristone	icosapent	0.88967387
flurbiprofen	etoposide	4.708054603	icosapent	fluvastatin	0.88336584
bosutinib	naproxen	4.70581433	latanoprost	vinblastine	0.873591933
celecoxib	fluvastatin	4.688065183	icosapent	eplerenone	0.854076728
olopatadine	propranolol	4.686183315	vinblastine	paclitaxel	0.847161574
bosutinib	cetirizine	4.635422676	fluoxetine	propranolol	0.826045669
pirfenidone	alitretinoin	4.610614718	icosapent	decitabine	0.817825677
pimozide	etoposide	4.601150547	nifedipine	fluvastatin	0.798203369
celecoxib	cladribine	4.587336152	fluoxetine	capsaicin	0.796085002
mifepristone	naproxen	4.58555375	nifedipine	eplerenone	0.785296749
eplerenone	etoposide	4.585188848	rofecoxib	propranolol	0.779641852
bosutinib	paclitaxel	4.540059939	capsaicin	rofecoxib	0.772138029
azacitidine	etoposide	4.529817131	icosapent	paclitaxel	0.765305804
mifepristone	cetirizine	4.468475762	prazosin	icosapent	0.759802618
bosutinib	clozapine	4.435017662	capsaicin	vinblastine	0.754512978
celecoxib	naproxen	4.419191628	mifepristone	fluvastatin	0.75225055
celecoxib	cetirizine	4.373932222	latanoprost	icosapent	0.748397561
icosapent	etoposide	4.357724512	fluvastatin	eplerenone	0.747470759
propranolol	alitretinoin	4.344204252	mifepristone	eplerenone	0.741288485

olopatadine	alitretinoin	4.342614654	vinblastine	propranolol	0.737149843
paclitaxel	celecoxib	4.323127055	decitabine	fluvastatin	0.710599972
etoposide	fluvastatin	4.299773405	fluoxetine	clozapine	0.707588585
mifepristone	paclitaxel	4.285419095	fluoxetine	lamivudine	0.693021373
etoposide	formoterol	4.275664736	decitabine	eplerenone	0.690029722
chloroquine	nifedipine	4.26448157	rofecoxib	clozapine	0.676532125
clozapine	celecoxib	4.260247973	fluvastatin	paclitaxel	0.667456932
prazosin	propranolol	4.220401056	fluoxetine	pimozide	0.663364298
capsaicin	rofecoxib	4.219521381	nifedipine	paclitaxel	0.663064407
etoposide	cladribine	4.200162539	rofecoxib	lamivudine	0.662887455
bosutinib	lamivudine	4.178356137	nifedipine	decitabine	0.662039844
prazosin	fluoxetine	4.161872005	vinblastine	clozapine	0.649894409
latanoprost	capsaicin	4.152423037	icosapent	propranolol	0.64915786
prazosin	olopatadine	4.121232527	eplerenone	paclitaxel	0.646839154
mifepristone	clozapine	4.099870921	capsaicin	icosapent	0.642909907
levofloxacin	propranolol	4.046160452	vinblastine	lamivudine	0.637116099
capsaicin	azelastine	4.017224452	prazosin	fluvastatin	0.636407953
capsaicin	vinblastine	4.008217454	rofecoxib	pimozide	0.634248867
celecoxib	lamivudine	4.006702222	prazosin	eplerenone	0.630360052
prazosin	alitretinoin	4.005527126	latanoprost	fluvastatin	0.62930254
etoposide	naproxen	3.990820744	mifepristone	paclitaxel	0.622369532
chloroquine	rofecoxib	3.962847791	latanoprost	eplerenone	0.62200079
etoposide	cetirizine	3.932696976	mifepristone	decitabine	0.617107792
levofloxacin	olopatadine	3.93226569	decitabine	paclitaxel	0.609352326
chloroquine	vinblastine	3.927678689	vinblastine	pimozide	0.609276008
levofloxacin	fluoxetine	3.908495628	fluoxetine	azacitidine	0.591557235
mifepristone	lamivudine	3.878883277	rofecoxib	azacitidine	0.573762646
levofloxacin	alitretinoin	3.863238268	icosapent	clozapine	0.563304991
etoposide	paclitaxel	3.854747036	vinblastine	azacitidine	0.56066577
pirfenidone	prazosin	3.844375432	propranolol	fluvastatin	0.556536573
capsaicin	flurbiprofen	3.794530056	icosapent	lamivudine	0.551943948
etoposide	clozapine	3.768363118	propranolol	eplerenone	0.544334624
chloroquine	azelastine	3.765337429	capsaicin	fluvastatin	0.538499037
capsaicin	eplerenone	3.716123324	capsaicin	eplerenone	0.533381582
chloroquine	fluvastatin	3.671161236	icosapent	pimozide	0.528098429
capsaicin	fluvastatin	3.658340227	prazosin	paclitaxel	0.519756239
capsaicin	azacitidine	3.655837523	latanoprost	paclitaxel	0.516726862
chloroquine	flurbiprofen	3.62152149	latanoprost	decitabine	0.505643447
chloroquine	latanoprost	3.593859352	prazosin	decitabine	0.503769611
chloroquine	eplerenone	3.565891928	decitabine	propranolol	0.47977426
pirfenidone	levofloxacin	3.56327113	icosapent	azacitidine	0.477735425
capsaicin	cladribine	3.552217277	clozapine	fluvastatin	0.477626651
etoposide	lamivudine	3.549753936	clozapine	eplerenone	0.469953854
chloroquine	cladribine	3.545812127	propranolol	paclitaxel	0.469035984
chloroquine	azacitidine	3.493772455	lamivudine	fluvastatin	0.467822975
capsaicin	icosapent	3.45564251	lamivudine	eplerenone	0.460398678
capsaicin	pimozide	3.412610242	fluvastatin	pimozide	0.447774986
capsaicin	formoterol	3.383878254	pimozide	eplerenone	0.440581738
nifedipine	propranolol	3.278141662	capsaicin	paclitaxel	0.439793741
chloroquine	icosapent	3.245435691	capsaicin	decitabine	0.426266594
capsaicin	naproxen	3.211040638	azacitidine	fluvastatin	0.400149482
nifedipine	alitretinoin	3.19989515	clozapine	paclitaxel	0.396657153
chloroquine	formoterol	3.17170315	azacitidine	eplerenone	0.396346788
nifedipine	olopatadine	3.128823347	clozapine	decitabine	0.39586448
capsaicin	cetirizine	3.11244417	nifedipine	propranolol	0.394234361
chloroquine	naproxen	3.059584819	lamivudine	paclitaxel	0.388324406
propranolol	rofecoxib	3.019624716	decitabine	lamivudine	0.387222586

fluoxetine	vinblastine	3.012993068	pimozide	paclitaxel	0.371866081
chloroquine	pimozide	2.981575488	decitabine	pimozide	0.37112295
propranolol	vinblastine	2.97815491	nifedipine	mifepristone	0.357019775
capsaicin	paclitaxel	2.953377004	mifepristone	propranolol	0.354493917
olopatadine	rofecoxib	2.936078943	azacitidine	paclitaxel	0.326803254
olopatadine	vinblastine	2.925747292	decitabine	azacitidine	0.316751461
fluoxetine	rofecoxib	2.923183219	latanoprost	propranolol	0.270047086
nifedipine	fluoxetine	2.919433144	prazosin	propranolol	0.254140034
chloroquine	cetirizine	2.917288459	nifedipine	clozapine	0.249341205
rofecoxib	alitretinoin	2.881328881	nifedipine	lamivudine	0.241329626
fluoxetine	fluvastatin	2.874869873	nifedipine	latanoprost	0.239254436
propranolol	azelastine	2.869800704	clozapine	propranolol	0.235188307
pirfenidone	vinblastine	2.82707505	nifedipine	pimozide	0.233757379
alitretinoin	vinblastine	2.804978892	propranolol	lamivudine	0.229069839
capsaicin	clozapine	2.79494323	propranolol	pimozide	0.220489038
olopatadine	azelastine	2.789019116	capsaicin	propranolol	0.215041568
propranolol	fluvastatin	2.776216429	mifepristone	clozapine	0.211976997
fluoxetine	azelastine	2.772159839	mifepristone	lamivudine	0.204626402
latanoprost	propranolol	2.765954498	nifedipine	prazosin	0.199468758
fluoxetine	cladribine	2.764200419	mifepristone	pimozide	0.198728434
propranolol	flurbiprofen	2.754344967	mifepristone	latanoprost	0.186756358
levofloxacin	prazosin	2.746332752	nifedipine	capsaicin	0.168781257
olopatadine	fluvastatin	2.742634706	propranolol	azacitidine	0.159793734
azelastine	alitretinoin	2.740060369	mifepristone	prazosin	0.144822318
pirfenidone	fluvastatin	2.734695609	clozapine	lamivudine	0.143452584
fluoxetine	flurbiprofen	2.712389123	latanoprost	clozapine	0.14154555
propranolol	eplerenone	2.710338088	clozapine	pimozide	0.138973293
latanoprost	alitretinoin	2.708197322	latanoprost	lamivudine	0.135707009
olopatadine	flurbiprofen	2.688757652	lamivudine	pimozide	0.134486798
fluoxetine	eplerenone	2.684120028	latanoprost	pimozide	0.132698954
propranolol	cladribine	2.683010079	nifedipine	azacitidine	0.125418484
chloroquine	paclitaxel	2.675843697	mifepristone	capsaicin	0.122541961
pirfenidone	rofecoxib	2.66869737	prazosin	clozapine	0.117553148
propranolol	azacitidine	2.656777058	prazosin	lamivudine	0.111870845
capsaicin	lamivudine	2.650171847	prazosin	pimozide	0.110206076
olopatadine	eplerenone	2.649275912	capsaicin	clozapine	0.099468048
olopatadine	cladribine	2.647290063	capsaicin	lamivudine	0.094659946
latanoprost	olopatadine	2.633227617	capsaicin	pimozide	0.093251295
pirfenidone	cladribine	2.621646375	mifepristone	azacitidine	0.091058849
fluoxetine	azacitidine	2.619932541	clozapine	azacitidine	0.073913016
alitretinoin	flurbiprofen	2.615176221	lamivudine	azacitidine	0.070340197
alitretinoin	fluvastatin	2.596053547	azacitidine	pimozide	0.069293452
olopatadine	azacitidine	2.594349657	prazosin	latanoprost	0.054022836
alitretinoin	eplerenone	2.569128542	latanoprost	capsaicin	0.04571163
pirfenidone	azelastine	2.527304124	latanoprost	azacitidine	0.033967536
alitretinoin	azacitidine	2.521512158	prazosin	capsaicin	0.00560845
nifedipine	pirfenidone	2.515968729	prazosin	azacitidine	0.004167544
alitretinoin	cladribine	2.512900799	capsaicin	azacitidine	0.003526383
pirfenidone	flurbiprofen	2.503300614			
pirfenidone	eplerenone	2.485916949			
propranolol	icosapent	2.472968517			
chloroquine	clozapine	2.441923816			
fluoxetine	latanoprost	2.434092528			
pirfenidone	azacitidine	2.420065846			
propranolol	formoterol	2.417354647			
olopatadine	icosapent	2.40454741			
fluoxetine	icosapent	2.393986257			

alitretinoin	icosapent	2.359708997
olopatadine	formoterol	2.349308895
fluoxetine	formoterol	2.335107612
chloroquine	lamivudine	2.333014633
propranolol	naproxen	2.327411137
alitretinoin	formoterol	2.308068869
prazosin	vinblastine	2.29659674
propranolol	pimozide	2.291988953
fluoxetine	naproxen	2.28799525
prazosin	fluvastatin	2.274608637
olopatadine	naproxen	2.271075447
pimozide	alitretinoin	2.237349741
propranolol	cetirizine	2.223449194
alitretinoin	naproxen	2.210935765
olopatadine	pimozide	2.187533598
pirfenidone	icosapent	2.185571122
prazosin	cladribine	2.169645511
olopatadine	cetirizine	2.160861657
fluoxetine	cetirizine	2.147799515
levofloxacin	fluvastatin	2.132993986
pirfenidone	formoterol	2.128855276
alitretinoin	cetirizine	2.122929655
levofloxacin	vinblastine	2.119183552
pirfenidone	naproxen	2.109331663
pirfenidone	latanoprost	2.079045655
prazosin	rofecoxib	2.062296534
propranolol	paclitaxel	2.047743687
fluoxetine	pimozide	2.040946965
levofloxacin	cladribine	2.027702747
alitretinoin	paclitaxel	1.976006581
prazosin	flurbiprofen	1.97410637
olopatadine	paclitaxel	1.973109483
prazosin	eplerenone	1.973014921
pirfenidone	cetirizine	1.958091484
prazosin	azelastine	1.947878182
prazosin	azacitidine	1.911502951
fluoxetine	paclitaxel	1.904440412
propranolol	clozapine	1.877149323
levofloxacin	rofecoxib	1.835087437
alitretinoin	clozapine	1.832399562
propranolol	lamivudine	1.791731243
olopatadine	clozapine	1.791599915
levofloxacin	eplerenone	1.790751868
levofloxacin	flurbiprofen	1.783384381
pirfenidone	pimozide	1.758733968
alitretinoin	lamivudine	1.744799176
levofloxacin	azelastine	1.729793039
levofloxacin	azacitidine	1.728838978
nifedipine	prazosin	1.72553218
olopatadine	lamivudine	1.713514632
pirfenidone	paclitaxel	1.692853993
prazosin	icosapent	1.688949747
fluoxetine	clozapine	1.6715448
prazosin	naproxen	1.660103027
vinblastine	fluvastatin	1.649633599
prazosin	formoterol	1.64078027
fluoxetine	lamivudine	1.610388443

rofecoxib	fluvastatin	1.595523918
rofecoxib	vinblastine	1.587911308
nifedipine	fluvastatin	1.583528314
vinblastine	cladribine	1.579917814
rofecoxib	cladribine	1.517305646
fluvastatin	cladribine	1.515284532
azelastine	fluvastatin	1.512858354
prazosin	cetirizine	1.50916688
azelastine	vinblastine	1.503063094
levofloxacin	icosapent	1.502873332
levofloxacin	naproxen	1.497520159
flurbiprofen	vinblastine	1.49501315
vinblastine	eplerenone	1.486387512
nifedipine	cladribine	1.484094277
flurbiprofen	fluvastatin	1.482271037
eplerenone	fluvastatin	1.467402624
nifedipine	vinblastine	1.466589782
levofloxacin	formoterol	1.457077921
vinblastine	azacitidine	1.445726473
pirfenidone	clozapine	1.440411078
azelastine	cladribine	1.438178945
azacitidine	fluvastatin	1.431884781
flurbiprofen	cladribine	1.413554512
eplerenone	cladribine	1.400645261
pirfenidone	lamivudine	1.397236125
prazosin	latanoprost	1.396921801
nifedipine	levofloxacin	1.387639152
azacitidine	cladribine	1.365809633
rofecoxib	eplerenone	1.344210739
levofloxacin	cetirizine	1.340200013
rofecoxib	flurbiprofen	1.339362092
latanoprost	fluvastatin	1.319031807
icosapent	fluvastatin	1.30667907
rofecoxib	azelastine	1.301563613
vinblastine	icosapent	1.300444605
rofecoxib	azacitidine	1.298232572
flurbiprofen	eplerenone	1.282995982
formoterol	fluvastatin	1.27434465
azelastine	eplerenone	1.270117938
vinblastine	formoterol	1.266093687
azelastine	flurbiprofen	1.264892436
vinblastine	naproxen	1.259266332
naproxen	fluvastatin	1.250194951
flurbiprofen	azacitidine	1.242716141
icosapent	cladribine	1.242621003
eplerenone	azacitidine	1.242029065
prazosin	paclitaxel	1.241106518
latanoprost	cladribine	1.23346462
azelastine	azacitidine	1.226205282
formoterol	cladribine	1.211439021
latanoprost	vinblastine	1.207871722
prazosin	pimozide	1.2059557
naproxen	cladribine	1.191903199
cetirizine	fluvastatin	1.172124491
vinblastine	cetirizine	1.16453537
nifedipine	eplerenone	1.145309764
rofecoxib	icosapent	1.130568733

rofecoxib	naproxen	1.124851635
cetirizine	cladribine	1.114264768
nifedipine	flurbiprofen	1.113839325
pimozide	fluvastatin	1.107018725
levofloxacin	latanoprost	1.101334868
eplerenone	icosapent	1.100862243
flurbiprofen	icosapent	1.096891368
rofecoxib	formoterol	1.096362143
nifedipine	azacitidine	1.086236651
eplerenone	naproxen	1.079227681
flurbiprofen	naproxen	1.07909303
eplerenone	formoterol	1.069874119
azelastine	icosapent	1.065935718
flurbiprofen	formoterol	1.065472458
azacitidine	icosapent	1.06320771
azelastine	naproxen	1.062138899
levofloxacin	paclitaxel	1.059046013
nifedipine	rofecoxib	1.056175826
azacitidine	naproxen	1.045048463
pimozide	cladribine	1.037483239
paclitaxel	fluvastatin	1.036425327
azelastine	formoterol	1.033454627
azacitidine	formoterol	1.032884629
pimozide	vinblastine	1.025154645
rofecoxib	cetirizine	1.008418655
vinblastine	paclitaxel	0.997933807
prazosin	clozapine	0.987683175
nifedipine	azelastine	0.984204127
eplerenone	cetirizine	0.98405534
flurbiprofen	cetirizine	0.980006752
paclitaxel	cladribine	0.978929089
prazosin	lamivudine	0.972862319
levofloxacin	pimozide	0.969622805
azelastine	cetirizine	0.950557197
azacitidine	cetirizine	0.950032921
latanoprost	eplerenone	0.93026988
nifedipine	naproxen	0.928230921
icosapent	naproxen	0.921214701
clozapine	fluvastatin	0.906653345
latanoprost	flurbiprofen	0.900703951
icosapent	formoterol	0.897882789
formoterol	naproxen	0.894684568
latanoprost	azacitidine	0.879373724
lamivudine	fluvastatin	0.874116549
nifedipine	icosapent	0.864971582
clozapine	cladribine	0.849703467
latanoprost	rofecoxib	0.840289637
vinblastine	clozapine	0.839606293
nifedipine	formoterol	0.82903681
icosapent	cetirizine	0.825860106
cetirizine	naproxen	0.822918426
lamivudine	cladribine	0.820610151
vinblastine	lamivudine	0.816497645
eplerenone	paclitaxel	0.815275442
flurbiprofen	paclitaxel	0.80395008
formoterol	cetirizine	0.800694576
pimozide	eplerenone	0.800470085

rofecoxib	paclitaxel	0.800460672
levofloxacin	clozapine	0.794125465
levofloxacin	lamivudine	0.793465974
azacitidine	paclitaxel	0.781286726
latanoprost	azelastine	0.781138469
pimozide	flurbiprofen	0.778441768
nifedipine	cetirizine	0.762536531
pimozide	azacitidine	0.759158998
azelastine	paclitaxel	0.751144457
latanoprost	naproxen	0.749529033
rofecoxib	pimozide	0.738027287
latanoprost	icosapent	0.688168238
azelastine	pimozide	0.687719689
paclitaxel	naproxen	0.672977019
latanoprost	formoterol	0.657986008
eplerenone	clozapine	0.655588622
icosapent	paclitaxel	0.655549688
pimozide	naproxen	0.648714478
eplerenone	lamivudine	0.644181737
flurbiprofen	clozapine	0.637547331
formoterol	paclitaxel	0.632720781
flurbiprofen	lamivudine	0.628499262
azacitidine	clozapine	0.621754655
azacitidine	lamivudine	0.612424804
latanoprost	cetirizine	0.605206381
rofecoxib	clozapine	0.604447688
pimozide	icosapent	0.604418899
rofecoxib	lamivudine	0.602909313
paclitaxel	cetirizine	0.581967777
pimozide	formoterol	0.579295414
azelastine	clozapine	0.563245537
azelastine	lamivudine	0.562777784
pimozide	cetirizine	0.532827867
clozapine	naproxen	0.531300093
naproxen	lamivudine	0.524313001
icosapent	clozapine	0.495021813
icosapent	lamivudine	0.493761937
formoterol	clozapine	0.474445565
formoterol	lamivudine	0.474051557
nifedipine	paclitaxel	0.46152181
clozapine	cetirizine	0.436388434
cetirizine	lamivudine	0.436026031
latanoprost	paclitaxel	0.34257261
pimozide	paclitaxel	0.322294559
paclitaxel	lamivudine	0.275860477
clozapine	paclitaxel	0.263960702
nifedipine	lamivudine	0.220861803
nifedipine	pimozide	0.220181107
nifedipine	clozapine	0.180329323
nifedipine	latanoprost	0.171196464
pimozide	lamivudine	0.154006502
latanoprost	lamivudine	0.136502798
clozapine	lamivudine	0.126132022
pimozide	clozapine	0.125626972
latanoprost	pimozide	0.11898828
latanoprost	clozapine	0.097451939

Supplemental Table S3. Top centrality genes in BRAF melanoma signaling networks. The top ten genes in the BRAF melanoma signaling network ranked by centrality score (Left) and top ten genes interacting with either vemurafenib or tretinoin ranked by centrality score (R) are shown below. The centrality score (average of closeness, betweeness and PageRank centrality metrics) is reported for each gene and the log2-fold change for all significantly differentially expressed genes in A375 melanoma cells treated with the vemurafenib+tretinoin combination relative to vehicle control (adjusted P<0.05, Benjamini-Hochberg correction); ns = not statistically significant.

Network Gene (Overall Centrality)	Centrality Score	log2-Fold Change (p.adj<0.05)	Network Gene (Drug Interaction)	Centrality Score	log2-Fold Change (p.adj<0.05)
NTRK1	0.685	-1.361	AKT1	0.670	-0.536
AKT1	0.670	-0.536	FN1	0.499	-0.681
YWHAZ	0.630	-0.502	JUP	0.464	ns
VIM	0.584	ns	CDK2	0.456	ns
NPM1	0.565	-0.366	CDH1	0.445	ns
CTNNB1	0.564	-0.430	PARP1	0.418	-0.282
ELAVL1	0.544	-0.543	BRAF	0.380	ns
YBX1	0.525	ns	ICAM1	0.372	ns
KRT10	0.504	ns	LMNA	0.366	-0.801
FN1	0.499	-0.681	RAC1	0.365	-0.406

Supplemental Results

Evaluation of key assumptions underlying signaling network construction and drug combination predictions

The first assumption of our network models that we examine is that the network structure is dependent, in part, on the collection of root genes and is not biased to an individual gene. To evaluate the stability of the network structures using the belief propagation algorithm, we performed a leave-one-out analysis for each root gene in two examples of generalized melanoma networks constructed using root genes from two sources: melanoma-associated genes in the DisGeNET (DGN) database (n=39 root genes) and significantly mutated genes (SMG) across the TCGA SKCM melanoma patient cohort (n=28 root genes). Both root gene lists were applied the same gene expression signature defined by primary melanoma tumors vs. normal skin samples from the GEO GSE15605 dataset. As is shown in **Figure S4**, the removal of individual root genes had a minimal effect on the original networks, where 98.5% and 98.8% of the network interactions are retained in the DGN (n=140 interactions) and SMG (n=165 interactions) networks, respectively.

Next, we sought to compare different algorithms used to cluster drugs into communities based on similarity matrices of drug-induced gene expression profiles. Therefore, we selected several clustering algorithms designed for analysis of similarity matrices: affinity propagation (AP), partitioning around medoids, hierarchical clustering and density-based clustering of applications with noise (DBSCAN). To compare the clustering results of each algorithm, we employed three internal measures to assess the optimal network structures to be maximized: average between cluster distance/within cluster distance, Dunn 2 index (minimum average dissimilarity between two clusters / maximum average within cluster similarity), average silhouette width (estimates average distance between clusters); as well as one internal measure to be minimized: within cluster sum of squares (**Figure S5**). We observed that the affinity

propagation algorithm exhibited the smallest cluster sum of squares and largest average between cluster distance/within cluster distance measure, while also demonstrating a comparably high Dunn 2 index and positive average silhouette width. These results support the use of the affinity propagation algorithm in our analysis to cluster drug communities. We provide two example visualizations of drug community clustering by the affinity propagation algorithm in **Figure S6** for drugs predicted for two generalized melanoma networks using the DGN and SMG root gene sets as described above. To determine if the drug communities clustered by the AP algorithm revealed functional similarities, we mapped each drug to mechanism classes from the KEGG DRUG database. We found that the majority of drugs clustered in the same community shared a common drug class, where drugs within each community exhibited an average of 54% and 52% drugs sharing a common class for the SMG and DGN networks, respectively.

We then examined the effects of several parameters on drug predictions. We found for signaling networks generated for each melanoma genomic subtype that, as expected, increasing lambda increased the signaling network size. In general, increasing lambda also increased the number of predicted drug combinations (**Figure S7**). However, for the *NF1* and TWT networks, the number of drug combinations plateaued at lambda = 0.025, and therefore we selected this parameter value to apply to all signaling networks for this study for consistency of comparisons. We then assessed the specificity of drug combination predictions for each major genomic subtype. We compared the results for each of the original subtype-specific melanoma signaling networks against those generated for two generalized melanoma networks (DGN, SMG) and random drug pairs. Of note, random drug combinations were selected from a set corresponding to the intersection of the validation dataset testing drug combinations across all four genomic backgrounds as well as the same set of all FDA approved therapies modeled in our method (**Figure S8A**). For each melanoma genomic subtype, we observed a higher positive

predictive value (PPV) as compared to either generalized model (SMG, DGN) or randomized drug pairs.

We next sought to determine if either the significantly mutated root genes or differentially expressed genes alone could account for the drug combination prediction results. We compared the number of validated drug combinations from the Bliss synergy matrix of melanoma cell lines representing each of the genomic backgrounds (48) using the original signaling networks, root genes only and differently expressed (DE) genes only (**Figure S8B**). Overall, our approach integrating differentially expressed genes and root genes performed better than using either data type alone. In fact, for *NRAS*, *NF1* and TWT networks, none of the validated drug combinations were recovered when using the differentially expressed genes alone. With the exception of the TWT network, the integrated network performed better than using root genes alone to predict the validated drug combinations.

Finally, we investigated the impact of re-wiring the signaling networks on predicting validated drug combinations to determine whether the results could be explained solely by the collection of genes involved in the network or if the specific interactions between the genes were more influential. Therefore, we generated random interaction pairs among the original genes for the *BRAF* signaling network (n= 100 permutations) fixing the same number of genes as in the original network. We found that the original interaction pairings for the *BRAF* signaling network resulted in the highest number of true positives, and that only 6/100 other random networks returned as few false positives (**Figure S9**).

Supplemental Methods

Cell line information

A375 melanoma cells were obtained from American Type Cell Culture Collection and maintained in DMEM with 10% FBS, penicillin/streptomycin and plasmocin.

Drug reagents

Vemurafenib 10mM/1mL in DMSO (S1267), tretinoin 10mM/1mL in DMSO and powder formulation (S1653) dissolved in DMSO were obtained from Selleckchem (Houston, TX, USA). Powder formulation of vemurafenib (202271) was purchased from MedKoo (Morrisville, NC, USA).

In vitro cell proliferation and viability assays

Cells were seeded in 96 well plates at a density of 5,000 cells/well. Proliferation of melanoma cells was measured in triplicate in 96 well plates via CellTiter 96 Aqueous MTS Cell Proliferation Assay kit (Promega) using 20µl/well as absorbance at 490 nm (Biotek Epoch instrument, Gen 5 software). The number of viable cells in culture was determined by quantitation of ATP present via CellTiter-Glo® Luminescent Cell Viability Assay kit (Promega) according to manufacturer's instructions using Vertias™ microplate luminometer (Turner BioSysetms, Inc.).

In vitro cell death assays

Cell cytotoxicity was measured in triplicate by CellTox™ Green Cytotoxicity Assay kit (Promega) using the manufacturer's Endpoint Assay Protocol and SpectraMax M2 fluorescence plate reader (Molecular Devices) with recommended excitation and emission wavelengths (490/520). Apoptosis-induced phosphatidylserine exposure and cell membrane integrity following drug treatment were measured in triplicate in melanoma cells by flow cytometric analysis using APC-Annexin V and PE-PI (BD Pharmigen). The corresponding gating strategy and representative flow cytometry scatterplots are visualized in **Supplemental Figure S14**

Enzymatic activity of caspase-3/7 was measured in duplicate using the Apo-ONE® Homogenous Caspase-3/7 Assay kit (Promega) according manufacturer's instructions and SpectraMax M2 fluorescence plate reader (Molecular Devices) with optimized excitation and emission wavelength settings (485/530).

In vivo studies

Female 11-week old athymic NCr-nu/nu mice (Charles River Laboratories, Inc., Strain #553) were used for tumor growth studies. A375 melanoma cells ($1.0x10^6$ cells) were suspended in PBS and were injected in the left and right flanks subcutaneously. Mice were weighed, and tumors were monitored for growth every other day. Subcutaneous tumor volumes were calculated via caliper measurement as follows: tumor volume = 0.5 x ((large diameter) x (small diameter)^2). Mice were randomized to drug treatment groups (n=8 mice/group) while maintaining proportional initial tumor size distributions. Drug treatments began on day 10 when tumors had grown 1,000 mm^3 in size. Powder formulations of vemurafenib and tretinoin were dissolved in 20% PEG-400 (v/v) + 5% TPGS (v/v) + 75% ddH2O. Vemurafenib alone (50 mg/kg,), tretinoin alone (10 mg/kg), vemurafenib+tretinoin combination or vehicle (10 mg/kg) was administered daily by oral gavage 6 days/week. Treatments continued for 15 days, at which time mice were sacrificed and tumors were weighed. Of note, one mouse designated for the combination group was excluded because the original tumor grew rapidly and ulcerated, which violated the ULAR institutional ethical guidelines, and was sacrificed 1 week post-tumor injection prior to drug administration. Resected tumors were fixed for 24 hours in 10% buffered formalin at 4°C, rinsed in ddH2O and stored in histocasettes in 70% EtOH at 4°C. Formalin-fixed tumor sections were stained with hematoxylin and eosin, and immunohistochemical stains for Ki67 (1:100, Clone SP6, ThermoFisher Scientific) and cleaved caspase 3 (1:180 Clone D175, Cell Signaling) using a Dako Autostainer Universal Staining System. In brief, after deparaffinization, antigen retrieval was performed with heating to 125°C. Blocking was performed with 3%

hydrogen peroxide followed by a serum free protein block. Slides were incubated at room temperature for 30 minutes in primary antibody. Slides were then incubated with biotinylated goat anti-rabbit (1:200, Vector) for 30 minutes followed by Vector RTU ABC Elite complex for 30 minutes. Probes were then detected with chromogen (DAB), and counterstained with hematoxylin. Images of tumor tissue were taken at 400x magnification. Immunohistochemical staining was scored using an immunoreactive scoring system, defined by the product of two semi-quantitative scores assessing staining intensity and percentage of positive cells (1-3).

RNA-seq analysis

A375 cells were plated in 100mm3 dishes at a seeding density of 2.2 10^6 cells and incubated at 37deg for 24 h before drug treatment. Drug treatments were applied in triplicate per condition: DMSO (5uM), tretinoin (5uM), vemurafenib (5uM), vemurafenib+tretinoin combination (5uM). Cells were harvested 8 hours after drug treatment with cold PSB and pellets were kept frozen at -80 deg. Total RNA was extracted using the Norgen Biotek total RNA purification plus kit (Cat #48300). RNA purity was assessed using Qubit RNA assay and BioAnalyzer RNA Nanochip tracings. RNA libraries were prepared for sequencing using the New England Biolabs NEBNext Ultra II Directional RNA Library Prep Kit for Illumina with the protocol for use with NEBNext Poly(A) mRNA magnetic isolation module. We used 200ng of total RNA for the construction of sequencing libraries. Paired-end (150bp) sequence reads were generated using the Illumina HiSeq 4000. Illumina HCS 3.4.0 software was used for basecalling. Sequenced reads were mapped to the human genome GRCh38p.10 GENCODE primary assembly GTF v27 and FASTA files using the STAR program (2.5.3a). We used the following protocol from Love *et al* for the R programming language to count reads, generate visualizations of the data and determine differentially expressed genes: http://www.bioconductor.org/help/workflows/rnaseqGene/. Read counts were calculated from aligned bam files using the *summarizeOverlaps* function in the *GenomicAlignments* R package

with the following parameters: mode="Union", singleEnd=FALSE, ignore.strand=TRUE, fragments=TRUE. We used rlog-transformed count data for exploratory data analysis and generation visualizations, including heat maps, principal component analysis and multi-dimensional scaling plots using the *ggplot2* package. The *DESeq2* package was used for differential gene expression analysis, which uses an empirical Bayes shrinkage method for log fold-change estimation and the Wald test for significance testing. Finally, the Benjamini-Hochberg (False Discovery Rate) method was used to correct p-values for multiple hypothesis testing, where an adjusted $P < 0.05$ cutoff was used to prioritize differentially expressed genes. Raw and processed RNAseq data is available in the GEO database under accession number GSE109731.

Statistics

Statistical analyses were performed using GraphPad Prism (v 7.03) software. Statistical significance of mean group difference was determined by two-tailed Student’s t test, and $P \leq 0.05$ was determined as the threshold for statistical significance. Cell proliferation experiments drug combination synergy was assessed using the Chou-Talalay method using CompuSyn software (4). Survival analyses were conducted via the cBioPortal (5) and PROGgene2 (6) tools using the log-rank statistic.

Supplemental References

1. Fedchenko N, Reifenrath J. Different approaches for interpretation and reporting of immunohistochemistry analysis results in the bone tissue - a review. Diagnostic pathology. 2014;9:221.
2. Leng Y, Yi M, Fan J, Bai Y, Ge Q, Yao G. Effects of acute intra-abdominal hypertension on multiple intestinal barrier functions in rats. Scientific reports. 2016;6:22814.
3. Remmele W, Stegner HE. [Recommendation for uniform definition of an immunoreactive score (IRS) for immunohistochemical estrogen receptor detection (ER-ICA) in breast cancer tissue]. Der Pathologe. 1987;8(3):138-40.
4. Chou TC. Drug combination studies and their synergy quantification using the Chou-Talalay method. Cancer research. 2010;70(2):440-6.
5. Cerami E, Gao J, Dogrusoz U, Gross BE, Sumer SO, Aksoy BA, et al. The cBio cancer genomics portal: an open platform for exploring multidimensional cancer genomics data. Cancer discovery. 2012;2(5):401-4.
6. Goswami CP, Nakshatri H. PROGgeneV2: enhancements on the existing database. BMC cancer. 2014;14:970.
